# Mucosal-associated invariant T cells promote inflammation and intestinal dysbiosis leading to metabolic dysfunction during obesity

**DOI:** 10.1038/s41467-020-17307-0

**Published:** 2020-07-24

**Authors:** Amine Toubal, Badr Kiaf, Lucie Beaudoin, Lucie Cagninacci, Moez Rhimi, Blandine Fruchet, Jennifer da Silva, Alexandra J. Corbett, Yannick Simoni, Olivier Lantz, Jamie Rossjohn, James McCluskey, Philippe Lesnik, Emmanuelle Maguin, Agnès Lehuen

**Affiliations:** 1 Université de Paris, Institut Cochin INSERM, CNRS F-75014 Paris, France; 2 CNRS, UMR8104 Paris, France; 3 Laboratoire d’Excellence INFLAMEX, Sorbonne Paris Cité, Paris, France; 4 INRA Micalis Institute, Jouy-en-Josas, Paris, France; 5 Department of Microbiology and Immunology, Peter Doherty Institute for Infection and Immunity, University of Melbourne, Melbourne, VIC Australia; 6 INSERM U932, Institut Curie, PSL University, Paris, France; 7 ARC Centre of Excellence in Advanced Molecular Imaging, Monash University, Clayton, VIC 3800 Australia; 8 Infection and Immunity Program and Department of Biochemistry and Molecular Biology, Biomedicine Discovery Institute, Monash University, Clayton, VIC Australia; 9 Institute of Infection and Immunity, Cardiff University, School of Medicine, Heath Park, Cardiff, CF14 4XN UK; 10 Institute of Cardiometabolism and Nutrition, ICAN, INSERM, 1166 Paris, France

**Keywords:** Chronic inflammation, Monocytes and macrophages, Mucosal immunology, Type 2 diabetes, Obesity

## Abstract

Obesity is associated with low-grade chronic inflammation promoting insulin-resistance and diabetes. Gut microbiota dysbiosis is a consequence as well as a driver of obesity and diabetes. Mucosal-associated invariant T cells (MAIT) are innate-like T cells expressing a semi-invariant T cell receptor restricted to the non-classical MHC class I molecule MR1 presenting bacterial ligands. Here we show that during obesity MAIT cells promote inflammation in both adipose tissue and ileum, leading to insulin resistance and impaired glucose and lipid metabolism. MAIT cells act in adipose tissue by inducing M1 macrophage polarization in an MR1-dependent manner and in the gut by inducing microbiota dysbiosis and loss of gut integrity. Both MAIT cell-induced tissue alterations contribute to metabolic dysfunction. Treatment with MAIT cell inhibitory ligand demonstrates its potential as a strategy against inflammation, dysbiosis and metabolic disorders.

## Abstract (web summary)

Inflammation, immune cells and the host microbiota are intimately linked in the pathophysiology of obesity and diabetes. Here the authors show mucosal-associated invariant T cells fuel inflammation in the tissues and serve a function in promoting metabolic breakdown, polarising macrophage populations and inducing dysbiosis of the intestinal microbiota.

## Introduction

Obesity is characterized by a chronic low-grade inflammation of the visceral adipose tissue (VAT), and this inflammation is a major driver of insulin resistance associated with the development of type 2 diabetes (T2D)^1–5^. VAT inflammation in obesity is the result of tissue accumulation of pro-inflammatory immune cells that include M1 macrophages, CD8^+^ T cells, Th17 CD4^+^ T cells, NK cells, and neutrophils^6–11^. In contrast, there is a reduction in the frequency of anti-inflammatory immune cells such as M2 macrophages, Foxp3^+^-regulatory T cells (Treg), eosinophils, and type 2 innate lymphoid cells (ILC2) that are associated with protection against insulin resistance through local control of inflammation in VAT^8,12–16^.

Gut mucosa contains many immune cells, as it is continually exposed to microbial antigens and ingested antigens from the diet^17–20^. Obesity promotes a pro-inflammatory shift in gut immune cell populations, characterized by reduced Foxp3^+^ Treg cells in the lamina propria and an increase of IFN-γ-producing Th1 and CD8^+^ T cells and IL-17-producing γδ T cells^21–24^. Obesity is also associated with changes in gastrointestinal flora, and microbiota transfer from obese patients or mice can impact body fat expansion, systemic inflammation, and insulin resistance^25–28^.

Mucosal-associated invariant T (MAIT) cells are innate-like T cells that typically express an invariant TCR α-chain (Vα7.2-Jα33 in humans and Vα19-Jα33 in mice), with a limited number of β-chains^29–31^. MAIT cells recognize the major histocompatibility complex-related molecule 1 (MR1), presenting antigens from certain bacteria and yeast^31,32^. MAIT cell antigens derive from bacterial metabolites of vitamin B2 synthesis^33,34^. We and others have revealed major MAIT cell alterations in T2D and obese patients^35–37^. In these patients, MAIT cells are less abundant in blood and they produce high level of IL-17 in VAT. Moreover, bariatric surgery-induced weight loss and associated improvement of metabolic and inflammatory status were accompanied by a significant increase of blood MAIT cell frequency^36^. All these observations prompted us to investigate the role of MAIT cells during obesity and T2D in mouse models.

We first analyzed MAIT cell alteration occurring in obese C57BL/6J (B6) mice, which were either fed high-fat diet (HFD) or deficient for leptin (ob/ob mice), then we deciphered the impact of MAIT cells in obesity using Vα19 transgenic B6 mice expressing elevated frequency of MAIT cells and MR1^−/−^ B6 mice devoid of MAIT cells. We analyzed glucose and lipid metabolism, inflammation status of VAT and ileum, immune cell populations in these tissues, and gut microbiota composition. We also assessed the therapeutic potential of blocking MAIT cells function with a non-activating ligand. Together our results revealed a deleterious role of MAIT cells in the development of metabolic dysfunction, which involved macrophage M polarization in adipose tissue as well as dysbiosis and gut leakiness.

## Results

### Impaired MAIT cell accumulation in adipose tissue and ileum

**Fig. 1:**
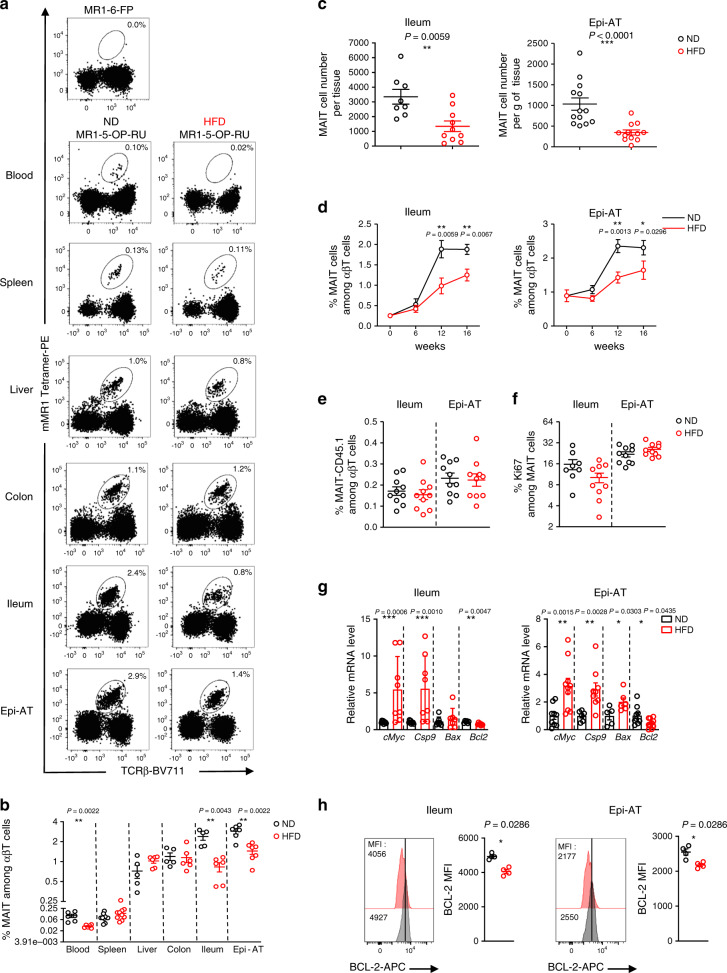
MAIT cell alterations during obesity. **a** Representative dot plots of MAIT cell and control MR1-Tet staining. The numbers represent MAIT cells frequency among αβT cells. **b** MAIT cell frequency among αβT lymphocytes in blood (ND *n* = 6; HFD *n* = 6), liver, colon, ileum (ND *n* = 5; HFD *n* = 6), Epi-AT (ND *n* = 6; HFD *n* = 7), and spleen (*n* = 10 per group). **c** MAIT cell absolute numbers in the ileum (ND *n* = 8; HFD *n* = 10) and MAIT cell number per gram of Epi-AT (ND *n* = 13; HFD *n* = 12). **d** MAIT cell frequency kinetic analysis of mice fed ND or HFD in the ileum (0 weeks *n* = 8; 6 weeks ND *n* = 11, HFD *n* = 14; 12 weeks ND *n* = 9, HFD *n* = 10; 16 weeks ND *n* = 10, HFD *n* = 13) and Epi-AT (0 weeks *n* = 8; 6 weeks ND *n* = 11, HFD *n* = 14; 12 weeks ND *n* = 15, HFD *n* = 15; 16 weeks ND *n* = 17, HFD *n* = 19). Pooled data from four independent experiments are represented. **e** Migration of MAIT cells into lean or obese recipient mice after 12 weeks of ND or HFD. Purified MAIT cells from CD45.1 Vα19 transgenic Cα^−/−^ mice were transferred into CD45.2 mice and analyzed 5 days later. Graphs represent CD45.1 MAIT cells frequency in the ileum (*n* = 10 per group) and Epi-AT (*n* = 10 per group). Pooled data from two independent experiments are represented. **f** Ki67 staining of MAIT cells from the ileum (ND *n* = 8; HFD *n* = 10) and Epi-AT (ND *n* = 10; HFD *n* = 10) of 12 weeks HFD or ND-fed mice. Pooled data from two independent experiments are represented. **g** qPCR analysis of apoptotic (*cMyc*, *Caspase9*, and *Bax*) and anti-apoptotic (*Bcl-2*) gene expression by FACS-sorted MAIT cells from mice fed ND (*n* = 8) or HFD (*n* = 9) for 12 weeks. Pooled data from two independent experiments are represented. **h** BCL-2 MFI in MAIT cells from the ileum and Epi-AT from mice fed ND or HFD for 12 weeks (*n* = 4 per group). For **b**, **c**, **e**, and **f**, each symbol represents an individual mouse (small horizontal lines indicate the mean ± S.E.M.). In **d**, **g**, and **h**, data are represented as mean ± S.E.M. Statistical analysis were performed by two-tailed Mann–Whitney test, *P < 0.05, **P < 0.01, ***P < 0.001 (see also Supplementary Fig. 1).

We first analyzed the frequency of MAIT cells in several tissues from B6 mice fed either HFD or normal diet (ND) for 12 weeks. Using mouse MR1 tetramers loaded with 5-OP-RU (TetMR1^+^)^38,39^, MAIT cells were identified as CD45^+^CD19^-^CD11b^-^TCRγδ^−^TCRαβ^+^TetMR1^+^ (Fig. 1a). As previously observed in obese and T2D patients, MAIT cell frequency was decreased in the blood of obese B6 mice compared with lean mice (Fig. 1a, b). MAIT cell frequency was also decreased in epididymal adipose tissue (Epi-AT) and ileum from obese mice compared with lean mice, whereas MAIT cell frequency remained unchanged in the spleen, liver, and colon. Of note, decreased frequency of MAIT cells in Epi-AT and ileum was also observed in terms of absolute numbers (Fig. 1c). In these tissues, most MAIT cells are CD4^−^CD8α^−^ (>80% of total MAIT cells), and this proportion was not modified under HFD (Supplementary Fig. 1a). Since obesity can induce early modification of immune cell homeostasis in adipose tissue and gut^24,40,41^, we performed a kinetic analysis of MAIT cell frequency in these tissues (Fig. 1d). No significant differences were observed at 6 weeks after beginning of HFD; however, differences observed at 12 weeks after HFD were sustained after 16 weeks of diet.

Interestingly, using another obesity mouse model, leptin-deficient (ob/ob) mice, we also observed a significant decreased of MAIT cell frequency in both ileum and Epi-AT when compared with (ob/+) littermate controls (Supplementary Fig. 3a, e).

Decreased frequency and number of MAIT cells in Epi-AT and ileum of obese mice could result from impaired recruitment and proliferation, and increased cell death. To analyze MAIT cell migration, CD45.1^+^ MAIT cells were injected into lean and obese B6 mice and analyzed in Epi-AT and ileum 5 days later (Supplementary Fig. 1b). Similar frequency of CD45.1^+^ MAIT cells, among αβT lymphocytes as well as among CD45^+^ cells, was observed in both lean and obese B6 mice (Fig. 1e). Analysis of MAIT cell proliferation based on Ki67 staining did not reveal any difference between MAIT cells from obese and lean mice (Fig. 1f; Supplementary Fig. 1c). In contrast, analysis of the expression of anti- and pro-apoptotic molecules in MAIT cells suggested increased apoptosis of MAIT cells from obese mice compared with lean mice. Transcript level of pro-apoptotic molecules such as *cMyc*, *Casp9* and *Bax* were increased, whereas the level of *Bcl-2* mRNA was decreased in MAIT cells during obesity (Fig. 1g). Difference in BCL-2 expression in the ileum and Epi-AT was confirmed at the protein level, and no such difference was observed in the spleen, liver, and colon (Fig. 1h; Supplementary Fig. 1d). Altogether these data suggest that MAIT cells in Epi-AT and ileum of obese mice are undergoing apoptosis leading to lower frequency.

### MAIT cells exhibit an inflammatory profile

**Fig. 2:**
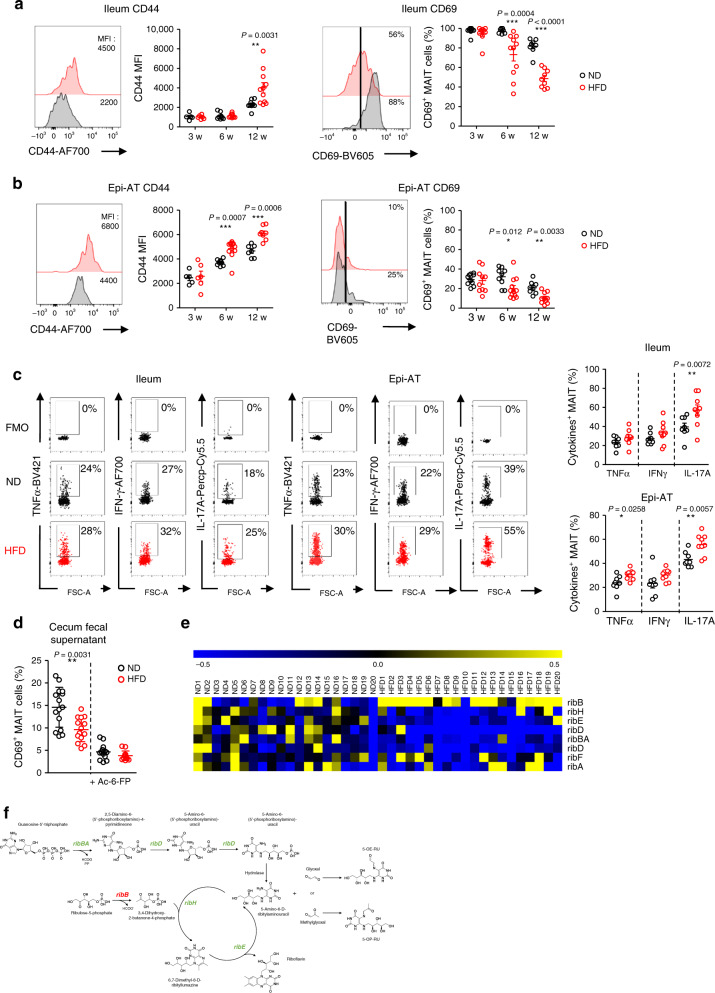
MAIT cell phenotype and function during obesity. **a**, **b** MAIT cell frequency kinetic analysis of B6 mice fed ND or HFD for 3, 6, and 12 weeks. **a** Graphs representing CD44 mean fluorescence intensity (MFI) (3 weeks ND *n* = 5, HFD = 6; 6 weeks ND *n* = 8, HFD *n* = 9; 12 weeks ND *n* = 9, HFD *n* = 11) and percentage of CD69^+^ MAIT cells (3 weeks ND *n* = 8, HFD = 9; 6 weeks ND *n* = 7, HFD *n* = 11; 12 weeks ND *n* = 8, HFD *n* = 9) among the total MAIT cells from the ileum. Pooled data from three independent experiments are represented. **b** Graphs representing CD44 mean fluorescence intensity (MFI) (3 weeks ND *n* = 5, HFD = 6; 6 weeks ND *n* = 9, HFD *n* = 12; 12 weeks ND *n* = 7, HFD *n* = 8) and percentage of CD69^+^ MAIT cells 3 weeks ND *n* = 10, HFD = 10; 6 weeks ND *n* = 9, HFD *n* = 12; 12 weeks ND *n* = 8, HFD *n* = 10) among total MAIT cells from Epi-AT. Pooled data from three independent experiments are represented. Representative histograms of staining on MAIT cells from the ileum and Epi-AT. **c** Intracellular staining of MAIT cells for TNFα, IFN-γ, and IL-17A. Frequency of positive MAIT cells from the ileum and Epi-AT of lean (*n* = 8) and obese (*n* = 9) mice are indicated. Pooled data from three independent experiments are represented. **d** Analysis of the abundance of MAIT cell-activating ligands using a bioassay based on the activation of purified MAIT cells by fecal supernatants from the cecum of HFD-fed (*n* = 14) mice or ND-fed (*n* = 15) mice in the presence of Ac-6-FP when indicated. Pooled data from four experiments are represented. **e** Heatmap representing microbial gene level abundance of key riboflavin biosynthesis enzymes from ND-fed mice and 12 weeks HFD-fed mice (*n* = 20 for per group). **f** The Riboflavin biosynthesis pathway in green decreased enzyme gene expression, and in red, increased enzyme gene expression. For **a**–**d**, each symbol represents an individual mouse (small horizontal lines indicate the mean ± S.E.M.). Statistical analysis was performed by two-tailed Mann–Whitney test, *P < 0.05, **P < 0.01, ***P < 0.001 (see also Supplementary Figs. 2–4).

Next, we analyzed the phenotype and cytokine production by MAIT cells from different tissues of mice fed ND or HFD. The expression of the maturation/effector marker CD44 was significantly increased on the surface of MAIT cells from Epi-AT and ileum of mice fed HFD compared with mice under ND (Fig. 2a, b). In parallel, a CD69 activation/retention marker was significantly decreased in both tissues from obese mice (Fig. 2a, b). Of note, there was no modification of CD44 and CD69 expression on MAIT cells from the spleen, and only slight modifications were seen in the liver and colon (Supplementary Fig. 2a, b).

Cytokine production analyses by qPCR and flow cytometry showed that ileum MAIT cells produced more IL-17A, and Epi-AT MAIT cells produced more TNF-α and IL-17A in obese mice (Fig. 2c; Supplementary Fig. 2c–e). Increased cytokine production was also observed in MAIT cells from the liver, but not colon (Supplementary Fig. 2f). Analysis of cytokine and chemokine receptor transcripts, usually associated with Th1 response (IL-18R) and Th17 response (CCR6), in MAIT cells from obese compared with lean mice showed overexpression of these receptors by MAIT cells isolated from ileum and Epi-AT, respectively (Supplementary Fig. 2c). According to overexpression of *Il18r* mRNA by MAIT cells from the ileum of obese mice, immunofluorescence staining showed an increased expression of *T-bet*, a key transcription factor of Th1 cytokines^42^ (Supplementary Fig. 2g, h). Together these data show that MAIT cells are activated and produce inflammatory cytokines in both Epi-AT and ileum from obese mice.

In ob/ob mice, similarly as in HFD-fed mice, there was an increased expression of CD44 on MAIT cells from Epi-AT, and a decreased expression of CD69 on MAIT cells from both ileum and Epi-AT when compared with (ob/+) littermate controls (Supplementary Fig. 3b, c, f, g). Moreover, a higher frequency of MAIT cells from the ileum and Epi-AT produced pro-inflammatory cytokines (IL-17A in ileum, TNF and IL-17A in Epi-AT) (Supplementary Fig. 3d, h).

Next, we investigated whether MAIT cell activation was associated with increased abundance of MAIT cell ligand produced by gut microbiota from obese mice. Using two bioassays, we assessed the ability of gut microbiota from lean and obese mice to activate MAIT cells (Fig. 2d; Supplementary Fig. 4a, b). There was a decreased activation of MAIT cells with cecum microbiota from obese mice compared with those from lean mice. Of note, this activation was MR1-specific, as it was inhibited in presence of non-activating ligand acetyl-6-formylpterin (Ac-6-FP)^43,44^ or blocking MR1 antibody^45^. To determine whether microbial community gene content contributes to lower level of MAIT cell agonist ligand upon HFD, we performed random shotgun sequencing of cecal microbial DNA from ND- or HFD-fed mice. The Kyoto Encyclopedia of Genes and Genomes (KEGG) orthology analysis showed that the riboflavin biosynthesis pathway was significantly downregulated in samples from HFD compared with ND-fed mice (Supplementary Fig. 4c). More precisely *ribBA*, *ribD*, *ribH*, and *ribE* genes were less abundant, whereas *ribB* gene was more abundant in microbiota from HFD-fed mice, and these differences could lead to decrease synthesis of MAIT cell agonist ligands (Fig. 2e, f; Supplementary Fig. 4d). Together bioassay and metagenomic data suggest that local activation of MAIT cells is not due to elevated presence of activating ligands, but rather to the pro-inflammatory milieu of Epi-AT and ileum of obese mice.

### MAIT cells promote metabolism dysfunction during obesity

**Fig. 3:**
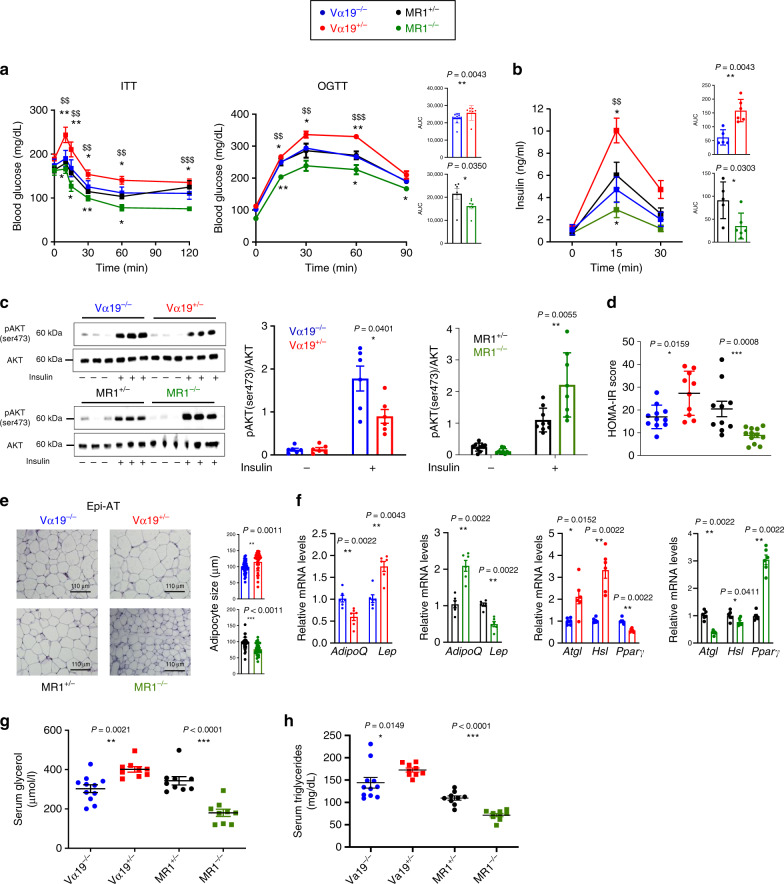
MAIT cells induce metabolic adipose tissue dysfunction during obesity. **a** ITT and OGTT in Vα19^+/−^ and MR1^−/−^ mice and their littermate controls fed with HFD during 12 weeks (Vα19^−/−^ *n* = 12, Vα19^+/−^ *n* = 7, MR1^+/−^ *n* = 7, MR1^−/−^ *n* = 7). $ represents statistics between Vα19^+/−^ and MR1^−/−^ mice. Pooled data from two independent experiments are represented. **b** Plasma insulin during OGTT (Vα19^−/−^ *n* = 5, Va19^+/−^ *n* = 6, MR1^+/−^ *n* = 5, MR1^−/−^ *n* = 6). $ represents statistics between Vα19^+/−^ and MR1^−/−^ mice. Pooled data from two independent experiments are represented. **c** Western blot of three individual mice (left) and quantification (right) of AKT phosphorylation (p-AKT-S473) in Epi-AT from HFD-fed Vα19^+/−^, MR1^−/−^ mice, and their littermate controls after insulin administration (2 UI/kg) in vivo (quantification *n* = 6 mice per group). **d** HOMA-IR index in Vα19^+/−^ (*n* = 10) and MR1^−/−^ (*n* = 12) mice, and their littermate controls (Vα19^−/−^ (*n* = 11) and MR1^+/−^ (*n* = 10)) fed with HFD during 12 weeks. Pooled data from two independent experiments are represented. **e** Representative hematoxylin & eosin staining of Epi-AT (Vα19^−/−^ *n* = 5, Va19^+/−^ *n* = 6, MR1^+/−^ *n* = 5, MR1^−/−^ *n* = 5). Scale bars, 110 μm. Quantification of adipocyte size in Epi-AT of Vα19^+/−^ and MR1^−/−^ HFD-fed mice, and their littermate controls. **f** Graphs showing the relative quantity of *adiponectin*, *leptin, Atgl*, *Hsl*, and *Pparγ* transcripts in Epi-AT from Vα19^+/−^ and MR1^−/−^ mice and their littermate controls fed with HFD during 12 weeks (*n* = 6 mice per group). **g**, **h** Serum glycerol and triglyceride concentrations in Vα19^+/−^ (*n* = 9) and MR1^−/−^ (*n* = 9) mice and their littermate controls (Vα19^−/−^ (*n* = 11) and MR1^+/−^ (*n* = 9) fed with HFD during 12 weeks. Pooled data from two independent experiments are represented. For **d**, **g**, and **h**, each symbol represents an individual mouse. All data are represented as mean ± S.E.M. All statistical analyses were performed by two-tailed Mann–Whitney test. * or ^$^*P* < 0.05 ** or ^$$^*P* < 0.01, *** or ^$$$^*P* < 0.001 (see also Supplementary Figs. 5, 6).

To determine the role of MAIT cells in the pathogenesis of T2D and obesity, we analyzed MR1^−/−^ B6 mice that lack MAIT cells, since the MR1 molecule is required for thymic development of MAIT cells^29,46–48^. Conversely, Vα19^+/−^ transgenic B6 mice that exhibit a tenfold increased frequency of MAIT cells were also analyzed (Supplementary Fig. 5a). To induce obesity, these mice and their respective littermates controls, MR1^+/−^ and Vα19^−/−^ mice were fed with HFD for 12 weeks. We first investigated glucose homeostasis in MR1^−/−^ and Vα19^+/−^ mice and performed insulin tolerance test (ITT) and oral glucose tolerance test (OGTT) after 12–16 weeks of HFD (Fig. 3a). Vα19^+/−^ mice had decreased insulin sensitivity than their littermate controls, whereas MR1^−/−^ mice presented an enhanced insulin tolerance when compared with their littermate controls. Similarly, while Vα19^+/−^ mice were more glucose intolerant, MR1^−/−^ mice had improved glucose tolerance. Glucose metabolism dysfunction was not due to impaired insulin secretion (Fig. 3b). The impact of MAIT cells on insulin resistance was confirmed at the tissue level by analysis of Akt phosphorylation, which is a readout of intracellular insulin signaling (Fig. 3c; Supplementary Fig. 5b, c). Relative amount of phosphorylated Akt in Epi-AT was increased in MR1^−/−^ mice and reduced in Vα19^+/−^ mice compared with their littermate controls, and similar data were observed in the liver and muscle from Vα19^+/−^ mice. In both fasted and fed MR1^−/−^ mice, basal blood glucose level was significantly decreased when compared with control littermates (Supplementary Fig. 5d). Conversely, in fasted and fed Vα19^+/−^ mice, basal blood glucose level was significantly increased. Moreover, basal serum insulin concentration and homeostatic model assessment of insulin resistance (HOMA-IR) index were decreased in MR1^−/−^ mice and increased in Vα19^+/−^ mice, under HFD (Fig. 3d; Supplementary Fig. 5e). Of note during the course of high-fat feeding period, there was no difference in weight gain, and at the end of the regime there was no differences in body weight, percentage of lean or fat mass and Epi-AT weight in both Vα19^+/−^ and MR1^−/−^ mice compared with their littermate controls (Supplementary Fig. 6a–e). No differences were observed regarding food intake, respiratory exchange ratio (RER) (Supplementary Fig. 6f, g). However, histology analysis showed that under HFD adipocyte size was increased in Epi-AT of Vα19^+/−^ mice compared with their littermate controls. Inversely, under HFD, adipocyte size in Epi-AT was smaller in MR1^−/−^ mice than in littermate controls (Fig. 3e; Supplementary Fig. 6h).

To further evaluate the impact of MAIT cells on Epi-AT function, expression of two key adipokines^49^, *adiponectin* and *leptin*, was measured by qPCR (Fig. 3f). *Adiponectin* expression was decreased, whereas *leptin* expression was increased in Vα19^+/−^ mice compared with their littermate controls. Opposite results were obtained in MR1^−/−^ mice. These results indicate that MAIT cells are involved in the deregulation of Epi-AT homeostasis. We next analyzed expression of two lipolytic enzymes, adipose triglyceride lipase (*Atgl)* and hormone sensitive lipase *(Hsl)*, involved in intracellular degradation of triglycerides in adipose tissue. Under HFD, while expression of both genes was increased in Vα19^+/−^ mice, compared with their control littermates, in MR1^−/−^ mice, expression of *Atgl* and *Hsl* (not significantly) was decreased (Fig. 3f). According to increased expression of these genes in Vα19^+/−^ mice, there was an elevated concentration of circulating glycerol and triglycerides in these mice fed HFD, and conversely there was a lower level of these lipids in the serum of MR1^−/−^ mice fed HFD (Fig. 3g, h). The expression of an adipogenic regulator, transcription factor peroxisome proliferator-activated receptor gamma (*Pparγ)*^50,51^, was significantly decreased in Epi-AT of Vα19^+/−^ mice and increased in Epi-AT of MR1^−/−^ mice (Fig. 3f). Altogether these data revealed the deleterious role of MAIT cells on glucose and lipid metabolism.

### MAIT cells exacerbate inflammation in adipose tissue

**Fig. 4:**
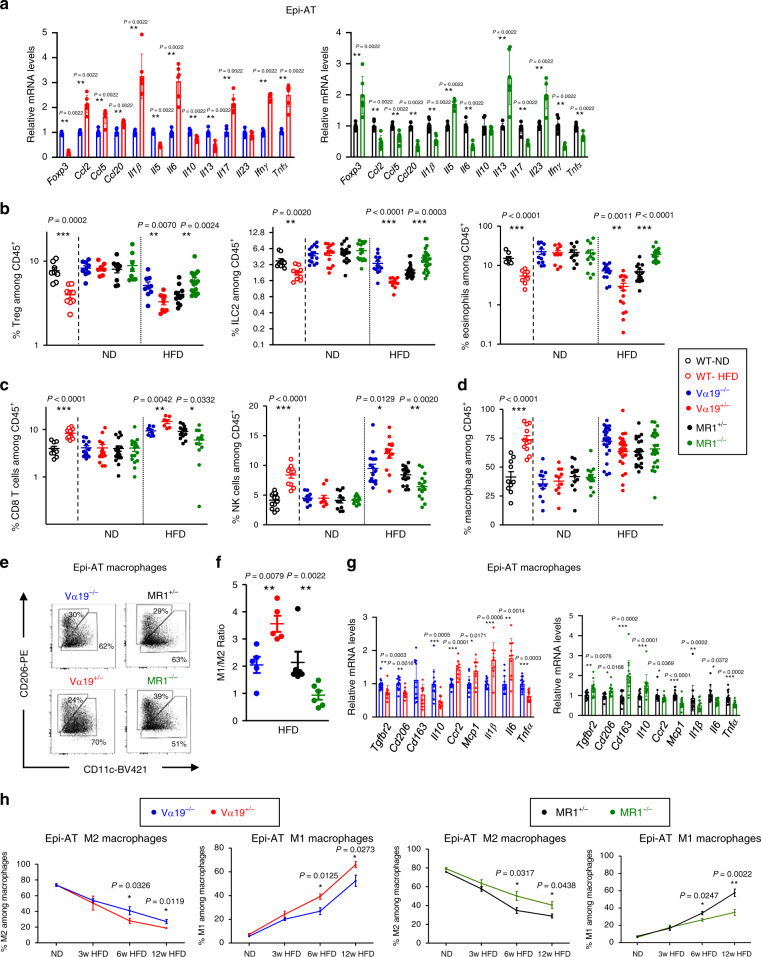
MAIT cells impact other immune cell populations in Epi-AT of obese mice and induce inflammation. **a** Graph showing the relative quantity of pro- and anti-inflammatory cytokine transcripts in Epi-AT of Vα19^+/−^ and MR1^−/−^ mice and their littermate controls fed with HFD during 12 weeks (*n* = 6 mice per group). **b**–**d** Graphs showing the frequency of Foxp3^+^ Treg cells, ILC2, eosinophils (**b**), CD8 αβT cells, NK cells (**c**), and macrophages (**d**) among CD45^+^ cells in the Epi-AT stroma-vascular fraction (SVF) of B6 mice fed with ND (*n* = 10) or HFD (*n* = 10), and Vα19^+/−^ (ND *n* = 12, HFD *n* = 13) and MR1^−/−^ (ND *n* = 10, HFD *n* = 16) mice and their respective littermate controls (Vα19^−/−^ (ND *n* = 12, HFD *n* = 11) and MR1^+/−^ (ND *n* = 12, HFD *n* = 17)) after 12–16 weeks of HFD or ND feeding. Pooled data from two to four experiments are represented. **e** Representative dot plot of Epi-AT macrophages sub-populations in Vα19^+/−^ and MR1^−/−^ mice and their respective littermate controls after 12–16 weeks of HFD or ND feeding. Data show the frequency of M2 (CD206^+^CD11c) and M1 (CD206^−^CD11c^+^) among CD11b^+^F4/80^+^ total macrophages. **f** M1 (CD206^−^CD11c^+^) and M2 (CD206^+^CD11c^+^) ratio in the Epi-AT of Vα19^+/−^ (*n* = 5), MR1^−/−^ (*n* = 6) and their littermate controls (Vα19^−/−^ *n* = 5 and MR1^+/−^ *n* = 6). **g** Graphs showing the relative quantity of pro-inflammatory M1 markers (*Ccr2, Mcp1, Il1β, Il6*, and *Tnfα*) and anti-inflammatory M2 markers (*Tgfbr2, Cd206, Cd163*, and *Il10*) transcripts in Epi-AT macrophages from Vα19^+/−^ (*n* = 10) and MR1^−/−^ (*n* = 14) mice and their respective littermate controls (Vα19^−/−^ *n* = 13 and MR1^+/−^ *n* = 16) fed on HFD during 12 weeks. **h** Kinetic analysis of M2 macrophages and M1-macrophages frequency of Vα19^+/−^ and MR1^−/−^ mice and their respective littermate controls fed with ND or HFD during 3, 6, and 12 weeks (*n* = 6 per group for each time point). In **a**, **e**, and **h**, all data are represented as mean ± S.E.M. For **b**–**d** and **g**, each symbol represents an individual mouse (small horizontal lines indicate the mean ± S.E.M.). All statistical analyses were performed by two-tailed Mann–Whitney test. *P < 0.05, **P < 0.01, ***P < 0.001 (see also Supplementary Figs. 7, 8, 9 and 10).

Since MAIT cells from Epi-AT of obese B6 mice produced high level of pro-inflammatory cytokines, we investigated whether MAIT cells had a general impact on the inflammatory status of these tissues. In Epi-AT, transcript level of cytokines known to be involved in inflammation (*Ccl2*, *Ccl5*, *Il1β*, *Il6*, *Il17a*, *Ifnγ*, and *Tnfα*) were significantly increased in Vα19^+/−^ mice compared with their littermate controls, whereas in MR1^−/−^ mice expression of these genes was decreased (Fig. 4a). Opposite results were observed for molecules associated with regulatory function in Epi-AT (*Foxp3*, *Il5*, and *Il13*). The inflammatory status of tissue was confirmed at the immune cell level and corroborated by the frequency of conventional T cells expressing RORγt a key transcription factor for IL-17 production^52^ (Supplementary Fig. 7a, b). Of note, under ND only slight differences were observed in Vα19^+/−^ mice compared with their controls (Supplementary Fig. 7c).

Since MAIT cells played a key role in the inflammatory status and function of Epi-AT during obesity, we investigated whether MAIT cells influenced frequency of other immune cells in this tissue from the different mouse lines fed ND or HFD (Supplementary Figs. S8–S10). In agreement with *Foxp3* mRNA level in HFD-fed mice, there was a decreased frequency of Foxp3^+^ Treg cells in Vα19^+/−^ mice and an increased frequency of Foxp3^+^ Treg cells in MR1^−/−^, compared with their respective littermate controls (Fig. 4b; Supplementary Fig. 10a). Similar modification was observed for ILC2 and eosinophils, two other regulatory immune cells in Epi-AT (Fig. 4b; Supplementary Fig. 10b, c). In contrast, frequency of inflammatory cells (conventional CD8^+^ T lymphocytes and NK cells) was increased in obese Vα19^+/−^ mice, whereas these cells were less frequent in obese MR1^−/−^ mice, compared with their respective littermate controls (Fig. 4c; Supplementary Fig. 10d). As controls, analyses of Epi-AT from WT B6 mice under ND and HFD confirmed previous studies^8,10,12^ showing decreased frequency of Treg cells, ILC2, and eosinophils and increased frequency of conventional CD8^+^ T cells and NK cells. While macrophage frequency from the different mouse lines under HFD was similar, MAIT cells had an impact on macrophage subsets (Fig. 4d–h; Supplementary Fig. 10e, f). There was a lower frequency of CD206^+^CD11c^−^ M2 and a higher frequency of CD206^−^CD11c^+^ M1 macrophages in obese Vα19^+/−^ mice, compared with their littermate controls. Opposite results were observed in MR1^−/−^ mice compared with their littermates (Fig. 4e, f). Phenotype of M1 and M2 macrophages were corroborated by their cytokine profiles (Fig. 4g). Kinetics analysis of immune cells in Vα19^+/−^ and MR1^−/−^ mice revealed that MAIT cells impact macrophage phenotype as early as 6 weeks of HFD, whereas changes in other immune cell populations were mainly observed at 12 weeks of HFD (Fig. 4h; Supplementary Fig. 10a–d). Taken together, these analyses of immune cell populations in Epi-AT strengthened the pro-inflammatory role of MAIT cells in obesity.

### Inflammatory crosstalk between MAIT cells and macrophages

**Fig. 5:**
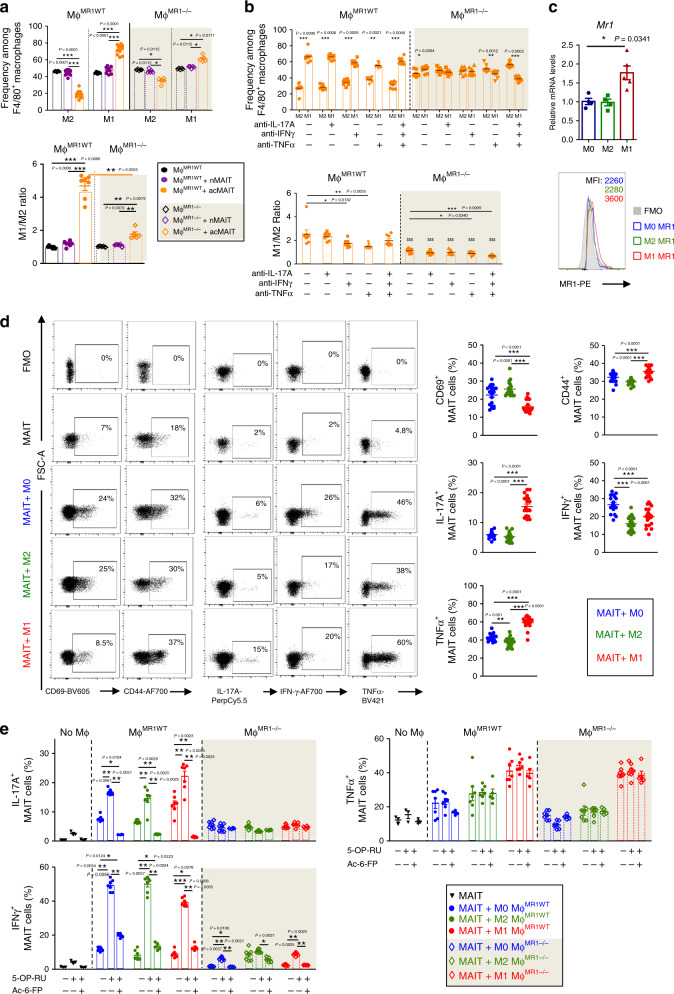
MAIT cells and macrophages crosstalk induces an inflammatory response in vitro. **a**, **b** Wild-type (clear background) or MR1^−/−^ (gray background) M0 macrophages were co-cultured for 72 h with spleen MAIT cells, either naive (nMAIT) or pre-activated (acMAIT). **a** Data showing the frequency of M1 macrophages (CD206^-^CD11c^+^) and M2 macrophages (CD206^+^CD11c^−^) among CD11b^+^F4/80^+^ total macrophages and M1/M2 ratio (*n* = 12 samples over two independent experiments). **b** Wild-type or MR1^−/−^ M0 macrophages were co-cultured for 72 h with spleen pre-activated MAIT cells (acMAIT) in presence of cytokine-neutralizing antibodies (anti-IL-17, anti-IFNγ, and/or anti-TNF). Data show the frequency of M1 macrophages (CD206^−^CD11c^+^) and M2 macrophages (CD206^+^CD11c^−^) among CD11b^+^F4/80^+^ total macrophages and M1/M2 ratio (*n* = 8 samples over two independent experiments). **c** Relative expression of *Mr1* in M0, M2, or M1 macrophages and surface staining for MR1 in in vitro-differentiated macrophages. Culture media contained 100 μM of Ac-6-FP. **d**–**e** Spleen nMAIT cells were co-cultured with M0-, M2-, or M1-differentiated macrophages for 48 h in presence of 5-OP-RU agonist molecule. **c** Data showing the frequency of M1 macrophages (CD206^−^CD11c^+^) and M2 macrophages (CD206^+^CD11c^−^) among CD11b^+^F4/80^+^ total macrophages co-cultured with acMAIT cells in medium with or without anti-IL-17A, anti-IFNγ and/or anti-TNFα neutralizing antibodies (*n* = 4 samples). **d** CD44 and CD69 surface marker expression on nMAIT cells and intracellular staining of nMAIT cells for TNFα, IFNγ, and IL-17A (*n* = 20 samples from four independent experiments). **e** Intracellular staining of MAIT cells co-cultured either with WT M0, M2, or M1 macrophages (round symbol, clear background) or MR1^−/−^ M0, M2, or M1 macrophages (diamond symbol, gray background) for TNF-α, IFN-γ, and IL-17A (*n* = 7 samples). Data are representative of at least three independent experiments. For **a** and **a**–**e**, each symbol represents an individual mouse (small horizontal lines indicate the mean ± S.E.M.). In **b**, in M1/M2 ratio graph, $ symbol represents statistics between WT macrophages vs. MR1^−/−^ macrophages cultured in the same conditions. All statistical analyses were performed by two-tailed Mann–Whitney test. *P < 0.05, **P < 0.01, ***P < 0.001 (see also Supplementary Fig. 11).

Since macrophages represent the more abundant immune cell population in Epi-AT^53,54^ and that MAIT cells impacted their phenotype, we further investigated in vitro MAIT cell and macrophage crosstalk. M0-macrophages differentiated from bone marrow of B6 mice were cultured in presence of MAIT cells either naive (nMAIT) or pre-activated with CD3 and CD28 mAbs (acMAIT). Interestingly, acMAIT cells promoted differentiation of M0 into M1 macrophages, as indicated by their phenotype (CD206^−^CD11c^+^) and expression of *Il1β*, *Ccl2*, and *Il10* (Fig. 5a; Supplementary Fig. 11a, b). However, co-culturing acMAIT cells with M0 generated from MR1^−/−^ mice (MΦ^MR1−/−^) had only a moderate impact on their differentiation into M1 macrophages, as illustrated by M1/M2 ratio (Fig. 5a). In contrast to major requirement for MR1, blocking cytokines (IL-17, IFNγ, and TNFα) had only a limited impact on macrophage polarization in presence of acMAIT cells (Fig. 5b).

Next we analyzed reciprocal impact of macrophages on MAIT cell phenotype and function after co-cultures of nMAIT cells with M0, M1, or M2. Interestingly, M1 macrophages expressed higher levels of *Mr1/*MR1 than M0 and M2 macrophages (Fig. 5c). nMAIT cells cultured in presence of M1 macrophages expressed lower level of CD69 and higher level of CD44, IL-17A, and TNFα, as compared with cultures with M0 or M2 macrophages (Fig. 5d). These phenotype and cytokine profile recapitulate those observed in Epi-AT of obese mice. To assess the role of TCR–MR1 interaction in MAIT cell activation by macrophages, agonist and/or inhibitory ligands were added to cultures. Addition of 5-(2-oxopropylideneamino)-6-D-ribitylaminouracil (5-OP-RU) agonist^33,43^ induced IL-17A and IFN-γ production that was inhibited in presence of Ac-6-FP^43^. In contrast, TNFα production was independent of TCR triggering. Requirement for TCR–MR1 interaction for IL-17A and IFN-γ production was confirmed in co-cultures with MR1-deficient macrophages (Fig. 5e). Together these data demonstrated that MAIT cell and M1 macrophage crosstalk promotes their inflammatory function.

### MAIT cells induce gut alteration and dysbiosis

**Fig. 6:**
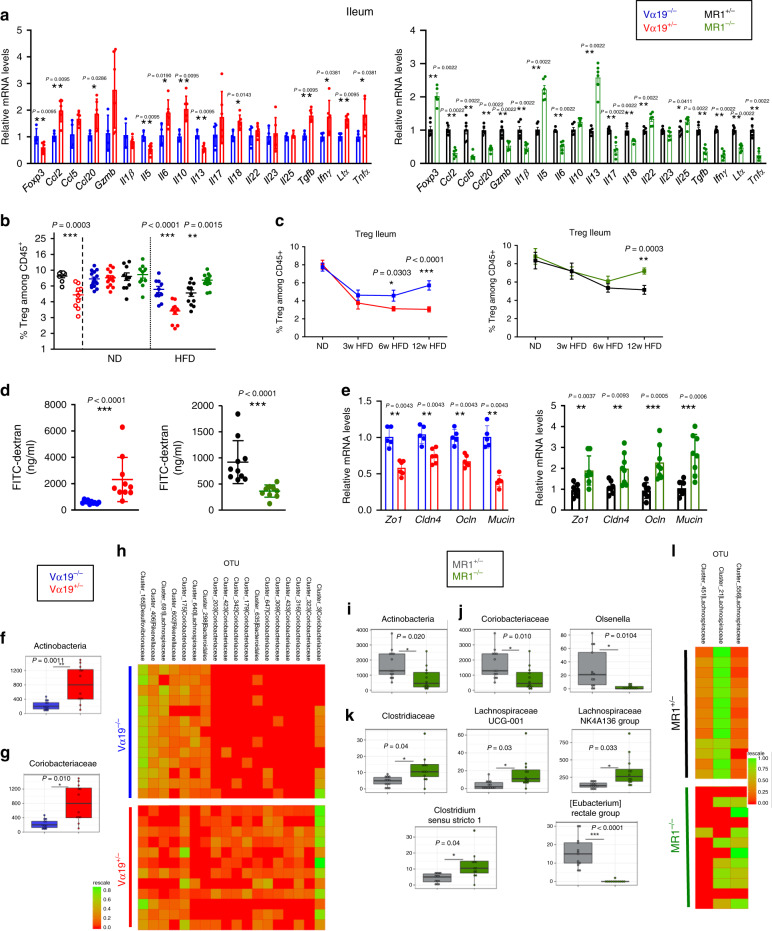
MAIT cells impact on ileum homeostasis and inflammation in obese mice. **a** Graph showing the relative quantity of cytokine transcripts in the ileum of Vα19^+/−^ and MR1^−/−^ mice and their littermate controls fed with HFD during 12 weeks (*n* = 6 mice per group). **b** Graphs showing the frequency of Foxp3^+^ Treg cells, among CD45^+^ cells in the ileum lamina propria of B6 mice fed with ND (*n* = 9) or HFD (*n* = 8), and Vα19^+/−^ (ND/HFD *n* = 14/10) and MR1^−/−^ (ND/HFD *n* = 10/14) mice and their respective littermate controls (Vα19^−/−^ ND/HFD *n* = 16/11 and MR1^+/−^ ND/HFD *n* = 10/11) after 12 weeks of HFD or ND feeding. Pooled data from two or three experiments are represented. **c** Kinetic analysis of Foxp3^+^ Treg cells among CD45^+^ cells of Vα19^+/−^ and MR1^−/−^ mice and their respective littermate controls (Vα19^−/−^ and MR1^+/−^) fed ND or HFD during 3, 6, and 12 weeks (*n* = 8 per group for each time). **d** FITC-dextran assay in Vα19^+/−^ and MR1^−/−^ mice and their littermate controls (*n* = 10 per group) fed HFD for 12 weeks. **e** Relative quantity of *ZO-1*, *Cldn4*, *Ocln*, and *mucin* transcripts in ileum epithelial cells of Vα19^+/−^, MR1^−/−^, and their respective littermate controls fed on HFD for 12 weeks (*n* = 6 per group). **f**, **g** Significant differences at the (**f**) phyla, (**g**) family levels between gut microbium of Vα19^−/−^ (*n* = 13) and Vα19^+/−^ (*n* = 12) HFD-fed mice. **h** Heatmap showing the significant differences between the OTU clusters of the Vα19^−/−^ (*n* = 13) and Vα19^+/−^ (*n* = 12) mice microbiota. Significant differences in (**i**) Actinobacteria phyla, (**j**) Coriobacteriaceae family and related *Olsenella* (genus), and (**k**) in the Clostridiaceae family and related genus between the MR1^+/−^ (*n* = 13) and MR1^−/−^ (*n* = 12) mice microbiota. **l** Heatmap showing OTU clusters abundance between MR1^+/−^ (*n* = 13) and MR1^−/−^ (*n* = 12) mice gut microbiota. Data in **f**, **g**, **i**, **j**, and **k** are presented as boxplots where the middle line is the median, the lower and upper hinges correspond to the first and third quartiles, the upper whisker extends from the hinge to the largest value no further than 1.5 × IQR from the hinge (where IQR is the inter-quartile range), and the lower whisker extends from the hinge to the smallest value at most 1.5 × IQR of the hinge. The fold change and the *P*-value of a Mann–Whitney test are indicated as mean ± S.E.M. For **b** and **d**, each symbol represents an individual mouse (small horizontal lines indicate the mean ± S.E.M.). In **a**, **c** and **e**, data are represented as mean ± S.E.M.. All statistical analyses were performed by two-tailed Mann–Whitney test. *P < 0.05, **P < 0.01, ***P < 0.001 (see also Supplementary Figs. 12, 13).

Similarly, as in Epi-AT, obesity induced major changes on MAIT cell frequency, phenotype, and cytokine production in ileum. Therefore, we investigated whether MAIT cells had impact in ileum inflammation and function. As in Epi-AT, MAIT cells promote inflammation in the ileum of Vα19^+/−^ mice on HFD, whereas the ileum of obese MR1^−/−^ mice was less inflamed (Fig. 6a). This observation was confirmed at immune cell transcript level and corroborated by frequency of Treg cells, ILC2, ILC3, and conventional αβT cells expressing RORγt (Fig. 6b, c; Supplementary Fig. 12a–e). In ND conditions, only small differences were observed in Vα19^+/−^ mice (Supplementary Fig. 12c).

To assess whether inflammatory status induced by MAIT cells during obesity had an impact on gut integrity, we analyzed FITC-dextran translocation to blood after oral gavage of HFD-fed Vα19^+/−^ and MR1^−/−^ mice and their respective controls (Fig. 6d). MAIT cells aggravated gut leakiness since translocation of FITC-dextran was increased in Vα19^+/−^ mice and reduced in MR1^−/−^ mice. In agreement with these data, there was higher mRNA level of tight junction proteins such as *zonulin-1*, *claudin-4* and *occludin*, and of *mucin*, major component of mucus layer, in epithelial cells from ileum of HFD-fed MR1^−/−^ mice and opposite results in HFD-fed Vα19^+/−^ mice (Fig. 6e). Since MAIT cells increased gut inflammation and leakiness in obese mice, we investigated whether MAIT cells had also an impact on gut microbiota composition. 16S-rRNA sequencing was performed on feces to evaluate relative abundance of different bacterial phyla in various mouse lines (Supplementary Table 1). Upon HFD, there was a significant increase of Actinobacteria phylum (Fig. 6f), Coriobacteriaceae family, and 11 bacterial OTU clusters assigned to this family (Fig. 6g, h; Supplementary Fig. 13a, c) in Vα19^+/−^ mice compared with their littermate controls. In HFD-fed MR1^−/−^ mice, there was a significant decrease of Actinobacteria phylum when compared with their littermate controls, whereas other phyla (Bacteroidetes, Firmicutes, Protobacteria, and Deferribacteres) remained unchanged (Fig. 6i; Supplementary Fig. 13b). In agreement, Coribacteriaceae family (Actinobacteria phylum) and *Olsenella* genus (a member of Coribacteriaceae family) decreased in MR1^−/−^ mice when compared with their littermates (Fig. 6j; Supplementary Fig. 13c). Conversely, an increase of Clostridiaceae (belonging to Firmicutes phylum) existed in MR1^−/−^ mice compared with their control group (Fig. 6k). Consistently at the genus level, *Clostridium* sensu stricto and two *Lachnospiraceae* groups also increased while *Eubacterium rectale* group displayed lower abundance in MR1^−/−^ mice than in control littermates (Fig. 6k), and three OTU clusters (556, 21, and 451) assigned to *Lachnospiraceae* varied between the two mice groups (Fig. 6l; Supplementary Fig. 13c). We also investigated whether changes in microbiota composition observed in HFD-fed Vα19^+/−^ and MR1^−/−^ mice impacted MAIT cell ligands, using bioassay. MAIT cells were less activated with cecum microbiota from Vα19^+/−^ mice when compared to their littermate controls. Conversely cecum microbiota from MR1^−/−^ mice increased MAIT cell activation when compared to their littermate controls (Supplementary Fig. 13d). Together these results showed that during obesity MAIT cells aggravate gut microbiota dysbiosis.

### Dysbiosis induced by MAIT cells impact ileum function

**Fig. 7:**
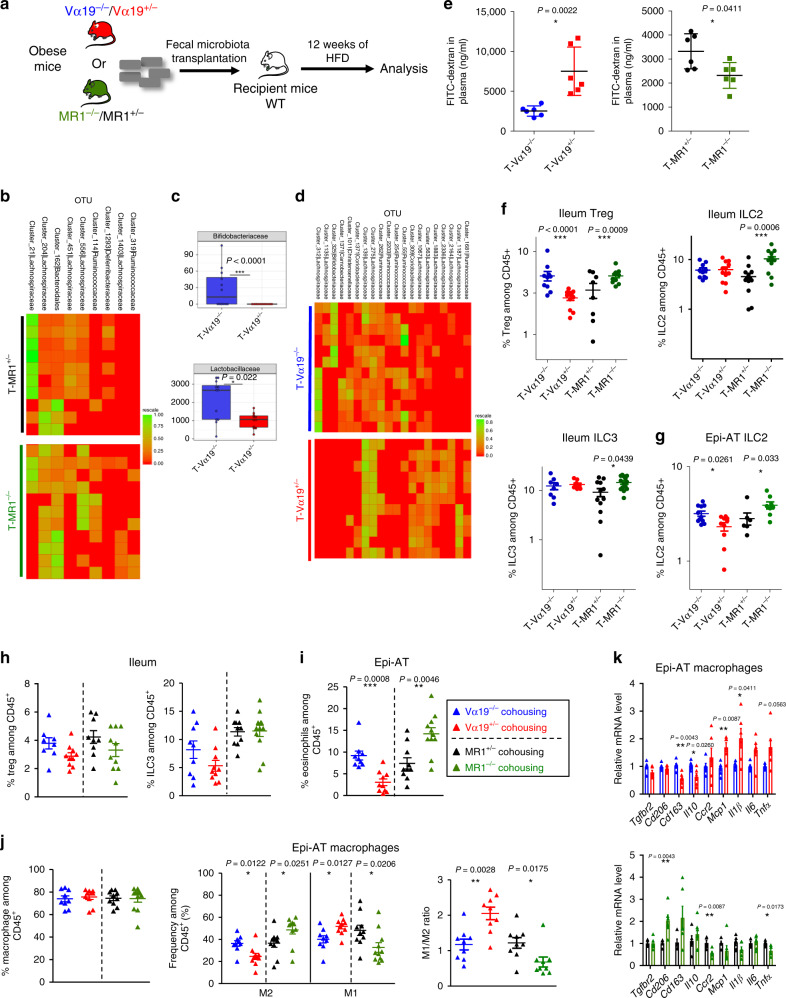
MAIT cells impact on microbiota, gut mucosa and adipose tissue fecal transplanted or co-housed mice. **a** Flowchart illustrating feces transfer procedure and data-analysis workflow. **b** Heatmap showing the significant differences in OTU clusters abundance between recipient mice T-MR1^+/−^ that received microbiota from obese MR1^+/−^ mice and the T-MR1^−/−^ mice that received microbiota from obese MR1^−/−^. **c** Significant differences at the genus level between the microbiota of recipient mice T-Vα19^−/−^ (*n* = 12) (microbiota from obese Vα19^−/−^ mice) and the T-Vα19^+/−^ (*n* = 11) (microbiota from obese Vα19^+/−^). **d** Heatmap showing the significant differences between the OTU clusters of the T-Vα19^−/−^ and T-Vα19^+/−^ mice microbiota. **e** Intestinal permeability measured by FITC-dextran assay (*n* = 6/group). **f**, **g** Graphs showing the frequency of immune cells in the ileum (**f**) and Epi-AT (**g**) of T-Vα19^−/−^ (*n* = 10), T-Vα19^+/−^ (*n* = 10), T-MR1^+/−^ (*n* = 10) and T-MR1^−/−^ (*n* = 10). **h** Graphs showing the frequency of Foxp3^+^ Treg cells and ILC3 among CD45^+^ cells in the ileum lamina propria (*n* = 9 mice/group). **i** Graphs showing the frequency of eosinophils among CD45^+^ cells in the Epi-AT stroma-vascular fraction (SVF) (*n* = 9 mice/group). **j** Graphs showing the frequency of total Epi-AT macrophages among CD45^+^, of M2 and M1 subsets among total Epi-AT macrophages and M1/M2 macrophage ratio (*n* = 9 mice/group. **k** Graphs showing the relative quantity of pro-inflammatory M1 markers (*Ccr2, Mcp1, Il1β, Il6*, and *Tnfα*) and anti-inflammatory M2 markers (*Tgfbr2, Cd206, Cd163*, and *Il10*) transcripts in Epi-AT macrophages from Vα19^+/−^ (*n* = 6) and MR1^−/−^ (*n* = 6) mice co-housed with their respective littermate controls (Vα19^−/−^ *n* = 6 and MR1^+/−^ *n* = 5) and fed on HFD during 12 weeks. Data in **d** are presented as boxplots, as mean ± S.E.M.. For **e**, **f**, and **h**–**j**, each symbol represents an individual mouse (small horizontal lines indicate the mean ± S.E.M.). In **k**, data are represented as mean ± S.E.M.. All statistical analyses were performed by two-tailed Mann–Whitney test. *P < 0.05, **P < 0.01, ***P < 0.001 (see also Supplementary Fig. 14).

To determine whether gut microbiota differences induced by MAIT cells play a role in metabolism dysfunction, microbiota transfer experiments were performed. Feces from HFD-fed Vα19^+/−^ mice, MR1^−/−^ mice, or their littermate controls were transferred into B6 recipient mice (Fig. 7a). 16S-rRNA sequencing analysis showed efficacy of gut microbiota transfer (Fig. 7b–d, Supplementary Fig. 14a, b). Bifidobacteriaceae (Actinobacteria phylum) and Lactobacillaceae (Firmicutes phylum) families were significantly lower in B6 mice transplanted with Vα19^+/−^ than littermate microbiota (Fig. 7c) and analysis at OTU level confirmed a differential distribution between various groups of recipient mice (Fig. 7d). Regarding gut mucosa function, as observed in donor mice, recipient B6 mice that had received feces from HFD-fed Vα19^+/−^ or MR1^−/−^ mice exhibited increased or decreased intestinal permeability, respectively, as compared with recipient B6 mice transferred with gut microbiota from their littermate controls (Fig. 7e). These modifications of gut permeability in recipient mice were associated with marked changes in Treg cell frequency in the ileum of mice transferred with feces from Vα19 and MR1^−/−^ mice and to increased ILC2 and ILC3 frequency in ileum from mice transferred with microbiota from MR1^−/−^ donors (Fig. 7f). These immune cell compositions were similar to the one observed in donor mice under HFD (Fig. 6b; Supplementary Fig. 12d). In contrast, gut microbiota transfer had only a moderate impact on Epi-AT immune cell populations (Fig. 7g). These data showed that during obesity, MAIT cells promote gut microbiota dysbiosis thereby increasing gut permeability, which is not sufficient to exacerbate metabolic dysfunction.

### M1-macrophage polarization is independent of microbiota

The role of microbiota was also evaluated by co-housing Vα19^+/−^ and MR1^−/−^ mice with their respective littermates for 12 weeks under HFD and metabolic tests (OGTT and ITT) were performed. Co-housing abolished metabolic differences previously observed when mice were bred in separate cages (Supplementary Fig. 14c, d). Analysis of immune cells showed that Foxp3^+^ Treg and ILC3 cell frequency was similar in the ileum of Vα19^+/−^ and MR1^−/−^ mice co-housed with their respective littermate controls (Fig. 7h). However, eosinophils, M2 and M1-macrophages frequencies remained significantly different in Epi-AT of Vα19^+/−^ and MR1^−/−^ mice when compared with their littermate controls (Fig. 7i, j). Gene expression analyses of macrophages corroborated flow-cytometry data (Fig. 7k). Together these results highlight the direct pro-inflammatory role of MAIT cells in Epi-AT, independently from gut microbiota dysbiosis.

### MAIT cell TCR-blocking ligand improves metabolic parameters

**Fig. 8:**
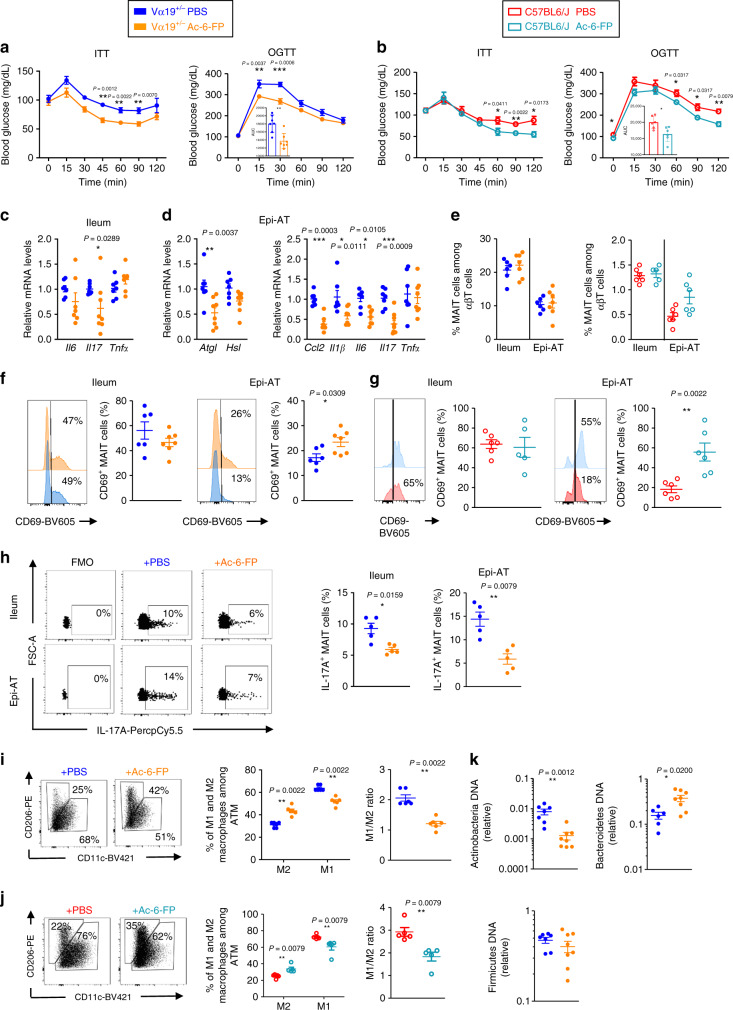
Ac-6-FP treatment during HFD improved metabolic parameters. **a**, **b** ITT and OGTT at 12 weeks of HFD in Vα19^+/−^ (*n* = 7) (**a**) or B6 WT (*n* = 6) (**b**) Ac-6-FP-treated mice and their respective controls. **c** Graph showing the relative quantity of *Il6, Il17,* and *Tnfα* transcripts in the ileum of Ac-6-FP-treated Vα19^+/−^ (*n* = 8) mice and Vα19^+/−^ (*n* = 7) control mice. **d** Graph showing the relative quantity of *Atgl*, *Hsl*, *Ccl2, Il1β, Il6, Il17*, and *Tnfα* in Epi-AT of Ac-6-FP-treated Vα19^+/−^ (*n* = 8) mice and Vα19^+/−^ (*n* = 7) control mice. **e** MAIT cells frequency among αβT cells in Vα19^+/−^ (*n* = 6) or B6 WT (*n* = 6) Ac-6-FP-treated mice and their respective controls. **f**, **g** show representative histograms of CD69 surface marker staining on MAIT cells from the ileum and Epi-AT of in Vα19^+/−^ (*n* = 6) (**f**) or B6 WT (*n* = 6) (**g**) Ac-6-FP-treated mice. **h** Intracellular staining of MAIT cells for IL-17A. Frequency of positive MAIT cells from the ileum and Epi-AT of Vα19^+/−^ control (PBS) (*n* = 5) and Vα19^+/−^ Ac-6-FP-treated (*n* = 5) mice are indicated. **I**, **j** The frequency of Epi-At macrophages among CD45+ cells. Data showing the frequency of M1 macrophages (CD206^−^CD11c^+^) and M2 macrophages (CD206^+^CD11c^−^) among total Epi-AT macrophages and M1/M2 ratio in Vα19^+/−^ (*n* = 6) (**i**) or B6 WT (*n* = 5) (**j**) Ac-6-FP-treated mice and their respective controls. **k** Actinobacteria, Bacteroidetes, and Firmicutes relative DNA expression compared with UTB. Bacterial DNA was extracted from cecum fecal content of Ac-6-FP-treated Vα19^+/−^ (*n* = 8) mice and Vα19^+/−^ (*n* = 7) control mice. In **a**–**d**, all data are represented as mean ± S.E.M.. For **e**–**k**, each symbol represents an individual mouse (small horizontal lines indicate the mean ± S.E.M.). All statistical analyses were performed by two-tailed Mann–Whitney test. *P < 0.05, **P < 0.01, ***P < 0.001. (See also Supplementary Fig. 15).

Since MAIT cell and macrophage crosstalk involved TCR–MR1 interaction, we performed in vivo treatment with the inhibitory ligand Ac-6-FP to assess whether it could improve metabolic parameters. Obese mice (B6 WT and Vα19^+/−^) were treated for 8 weeks, and such treatment ameliorated insulin sensitivity and glucose tolerance as compared with untreated mice (Fig. 8a, b). Of note, Ac-6-FP treatment of obese MR1^−/−^ mice did not have any effect on their glucose tolerance or insulin sensitivity (Supplementary Fig. 15a). Blocking MAIT cell activation decreased inflammation in the ileum (*Il17*) and Epi-AT (*Ccl2*, *Il1β*, *Il6*, and *Il17)* and improved Epi-AT function (*Atgl* and *Hsl* enzymes) mimicking Epi-AT status in MR1^−/−^ mice (Fig. 8c, d). Ac-6-FP treatment had no effect on MAIT cell frequency and their CD44 expression in both tissues, whereas it increased CD69 MAIT cell expression in Epi-AT (Fig. 8e–g; Supplementary Fig. 15b, c). Interestingly, MAIT cell production of IL-17A was significantly decreased in the ileum and Epi-AT of Ac-6-FP-treated mice (Fig. 8h), and M1/M2 ratio was decreased in Epi-AT (Fig. 8i, j; Supplementary Fig. 15d, e). Finally, Ac-6-FP treatment also impacted gut microbiota composition, as it significantly decreased Actinobaceria and increased Bacteroïdetes abundance (Fig. 8k). All these data suggest that blocking MAIT cell activation during obesity could be an efficient strategy to lower chronic inflammation, prevent dysbiosis, and improve metabolic parameters.

## Discussion

This study shows that obesity has an impact on MAIT cell frequency and function in Epi-AT and ileum and conversely MAIT cells promote chronic inflammation and gut dysbiosis, which together induced lipid and glucose metabolism deregulation (Supplementary Fig. 16). In obese mice, MAIT cell frequency and number decreased specifically in blood, ileum, and Epi-AT. These results in mouse models corroborated previous results in obese and/or T2D patients showing decreased blood MAIT cell frequency and number as well as decreased MAIT cell frequency in adipose tissue^35–37^. MAIT cells overexpressed pro-apoptotic genes (*Bax*, *cMyc*, and *Casp9*) and downregulated the anti-apoptotic Bcl-2/BCL-2 gene and protein in Epi-AT and ileum of obese mice compared with lean controls. Our previous data in humans showed that co-culture of MAIT cells with visceral adipose tissues from obese patients induced the downregulation of BCL-2^36^. Together these observations in humans and mice suggest that obesity chronic inflammation in VAT and ileum could promote MAIT cell apoptosis. Even though we have recently proposed that hyperglycemia could induce MAIT cell apoptosis^55^, since MAIT cell frequency is mainly reduced in adipose tissue and ileum, we favor the role of local inflammation. Indeed, in many inflammatory and autoimmune diseases, MAIT cells are activated and prone to activation-induced cell death by apoptosis^20,56,57^.

Another striking observation regarding MAIT cells in obesity is their inflammatory status and their propensity to promote inflammation in adipose tissue and ileum. In these tissues, MAIT cells upregulated expression of several molecules involved in inflammation, such as transcription factor (T-bet), chemokine/cytokine receptors (CCR6, IL-18R), and cytokines (TNFα and IL-17). Human studies also show overexpression of IL-17 by MAIT cells from adipose tissue of obese patients^35,36^. Studies of mouse models with high frequency of MAIT cells or MAIT cell deficiency demonstrate the role of these cells in inflammation of both adipose tissue and ileum. This was evidenced by gene expression analysis of whole tissues and purified total immune cells, as well as by characterization of various immune cell populations by flow cytometry. Kinetic analysis revealed that the impact of MAIT cells on adipose tissue eosinophils and macrophage phenotype could be observed after 6 weeks of HFD, before detectable alteration of glucose metabolism. However at 12 weeks of HFD, enhanced inflammation induced by MAIT cells was associated with exacerbated metabolic dysfunction in adipose tissue. In vitro mechanistic studies highlighted a bidirectional crosstalk between macrophages and MAIT cells. M1 macrophages activated IL-17 production by MAIT cells, in a MR1-dependent manner and even in absence of exogenous ligand. Conversely, activated MAIT cells favored differentiation of M1 macrophages. While such macrophage–MAIT cell crosstalk was also observed in vivo, it is not clear whether one of both cell types act as an initiator during obesity since we did not detect alteration of both cell types at 3 weeks of HFD. In vitro experiments demonstrated the role of TCR–MR1 recognition for IL-17 production by MAIT cells in presence of macrophages, whereas TNFα production was MR1-independent. The key role of MR1–TCR interaction in inflammation and metabolism dysregulation induced by MAIT cells was further evidenced by in vivo treatment with antagonist ligand (Ac-6-FP) blocking MR1 recognition by MAIT cells. These results highlighting the role of TCR–MR1 interaction in MAIT cell function is reminiscent of our recent studies showing MAIT cell activation of myofibroblast in the context of liver fibrosis^58^.

In parallel to the impact on tissue inflammation during obesity, MAIT cells also induced gut microbiota alteration. This was shown by bacteria 16S-rRNA sequencing, metagenomic as well as by two bioassays evaluating MAIT cell ligands abundance. There was an opposite effect on Actinobacteria abundance in MR1^−/−^ and Vα19^+/−^ mice compared with their control groups indicative of MAIT cell effect on gut microbiota composition. This impact of MAIT cells on Actinobacteria was confirmed by Ac-6-FP treatment. Of note, Coriobacteriaceae, which are altered according to MAIT cell status, have been associated to metabolic diseases and host lipid metabolism^59^. Other modifications of microbiota observed at family, order, genus, and OTU levels presented a strong coherence, and targeted microorganisms have already been associated to metabolic diseases and immunomodulation^60–62^. Gut microbiota alteration could be related to increased ileum inflammation. Gut microbiota transfer from HFD-fed mice harboring high frequency of MAIT cells induced ileum inflammation and gut mucosa dysfunction in recipient mice. Moreover, co-housing abolished difference in ileum inflammatory status of mice expressing various frequencies of MAIT cells. Together these data strengthened the role of gut microbiota in ileum inflammation induced by MAIT cells during obesity. Ileum inflammation is associated with loss of gut integrity that can promote endotoxemia, which favors systemic insulin resistance and T2D^63,64^.

Interestingly, our study reveals a decreased production of MAIT cell-activating ligand in gut microbiota from HFD mice. This was suggested by MAIT cell activation in bioassays and confirmed by gene abundance of enzymes *(RibBA, RibD*, and *RibE)* necessary for the biosynthesis of MAIT cell agonist ligands^33,65^. Only *RibB* gene leading to consumption of MAIT cell ligand precursor was more abundant upon HFD. Our data are reminiscent to metagenomic analyses in obese/T2D patients and mouse models showing impaired riboflavin synthesis in feces microbiota^27,66^. Therefore decreased abundance of MAIT cell agonist ligand could play a role in MAIT cell behavior during obesity. It is possible that lower activation ability of gut microbiota from obese mice might contribute to MAIT cell apoptosis in the ileum and Epi-AT, in addition to other local factors produced by these inflamed tissues. Decreased bacterial-derived agonist ligand availability might represent a way to dampen MAIT cell activation in inflammatory conditions, since our data showed that MAIT cell activation in vitro and in vivo in the context of obesity is MR1-dependant.

This study in obesity revealed the deleterious role of MAIT cells in promoting ileum inflammation, gut mucosa dysfunction, and gut microbiota alteration, whereas we had previously shown in mouse models of T1D that MAIT cells were protective by maintaining gut integrity^56^. This discrepancy prompts further analysis of intestinal MAIT cells in both pathologies to particularly determine their production of effector molecules that could impact other immune cells, epithelial cells, as well as gut microbiota composition. Recent studies have described an ambivalent role of MAIT cells^20,67,68^.

In summary, our data in mouse models of obesity not only strengthened our previous results showing MAIT cell alterations in blood and VAT from obese patients but also it revealed the mechanisms by which MAIT cells contribute to metabolic dysfunction. Mouse models, which allowed analysis of several tissues, revealed the pro-inflammatory profile of MAIT cells in both adipose tissue and ileum that is associated with gut microbiota and mucosa alteration. MAIT cells induced sustained adipose tissue inflammation and gut microbiota dysbiosis that are both required to induce insulin resistance and metabolic dysfunctions. Moreover, this study might open therapeutic strategies based on MAIT cell triggering to prevent insulin resistance and T2D.

## Methods

### Mice

All the mice used in this study were on C57BL/6J (B6) background. Vα19^+/−^ transgenic mice that contain a high frequency of MAIT cells (Vα19 Tg) and V*α*19^−/−^ littermates controls, MR1^−/−^ mice lacking MAIT cells and their MR1^+/−^ littermate controls, Vα19^+/−^ Cα^−/−^ B6 mice for transfer experiments. These mice have been previously described^69^. To better analyze MAIT cell phenotype, mouse lines were backcrossed with Rorc(γt)-GFP Tg C57BL/6J mice^70^. Leptin-deficient (ob/ob) males were purchased from Charles River at 11 weeks of age, and fed regular chow diet ad libitum until sacrifice.

All mice used in the studies were males, and in all experiments mice of different genotypes were separated at weaning, except when mentioned co-housing. At 8 weeks of age, mice from the different mouse lines (*n* = 5–7 per experiment) were fed with a 60 kJ% fat diet (SSNIFF ref# E15742-347) or with a 10% fat diet (ND) (SAFE, ref# A03 SP10) for 12 weeks. All animal experiments were approved by the ethical committee CEEA34 (APAFIS # 4838-2015111715473538) and conducted in accordance with the guidelines stated in the International Guiding Principles for Biomedical Research Involving Animals, developed by the Council for International Organizations of Medical Sciences (CIOMS). All mouse strains were bred and maintained in under specific pathogen-free conditions in the mouse facility of Cochin Institute, and at the “Centre exploration fonctionnelle (CEF)” at Paris Sorbonne University.

### Cell preparations

The fat pad adipose tissue was isolated from mice and digested with 5–10 mL of collagenase H solution (1 mg/mL, Roche ref# 11074059001) at 37 °C for 30 min with shaking (150 rpm). After digestion, adipocytes were removed by filtering through a 100-μm nylon mesh, and cell suspension was centrifuged for 5 min at 300 *g* to pellet the stroma-vascular fraction (SVF). SVF was washed with FACS buffer containing 5% fetal calf serum (FCS, Dominique Dutscher, S1810-500) and 0.1% sodium azide in PBS. Macrophages were directly analyzed from SVF, whereas other immune cells were further enriched on Percoll density gradients of 40 and 80% (GE Healthcare ref# 17–0891). The interface between the layers was collected and suspended in PBS containing 5% FCS and 0.1% sodium azide, to retrieve immune cells. After removal of fat tissue, feces and Payer’s patches, the intestine was extensively rinsed with HBSS without Ca^2+^ and Mg^2+^ containing 10 mM HEPES. Lamina propria cells were isolated using Lamina Propria Dissociation Kit (Miltenyi, 130-097-410) according to the manufacturer’s instructions, and the cells suspension were enriched by Percoll as for adipose tissue. Liver was perfused with RPMI 1640 medium (Gibco, 6187-010) supplemented with 5% FCS to remove circulating blood cells and then harvested. Liver was passed through 70-μm mesh, and cells suspension was collected after 2 min centrifugation at 48 *g* to avoid parenchymal cells. The supernatant was centrifuge again at 440 *g*, and the pellet was then resuspended in 40% Percoll layered onto 80% Percoll and centrifuged for 15 min at 780 *g* at room temperature. The immune cell fraction was collected at the interface and suspended in PBS containing 5% FCS and 0.1% sodium azide, to retrieve immune cells.

### Flow cytometry

Cell suspensions prepared from various tissues were stained at 4 °C in PBS containing 5% FCS and 0.1% sodium azide. Surface staining was performed with the following antibodies: anti-CD45 (30-F11), anti-TCRβ (H57), and anti-CD103 (M290) mAbs from BD Biosciences; anti-CD45 (30-F11), anti-CD8α (53-6.7), anti-CD45.1 (A20), anti-CD45.2 (104), anti-CD11c (N418), anti-CD206 (C068L2), anti-Sca-1 (D7), anti-NK1.1 (PK136), anti-CD19 (6D5), anti-CD4 (GK1.5), anti-CD11b (M1/70), anti-CD127 (A7R34), anti-CD25 (PC61), and anti-CD69 (HI-2F3) mAbs from Biolegend; anti-CD8α (53-6.7), anti-CD44 (IM7), anti-Siglec-F (1RNM44N), anti-TCRγδ (GL-3), and anti-F4/80 (BM8) mAbs from eBioscience.

Alpha-galactosylceramide-CD1d tetramer was prepared by the laboratory and coupled to streptavidin-BV421 (Biolegend). Biotinylated mouse MR1 tetramers loaded with the active ligand (5-OP-RU) were used to specifically identify MAIT cells; biotinylated MR1 tetramers loaded with the non-activating ligand acetyl-6-formylpterin (Ac-6-FP) were used as a negative control. MR1 tetramers were generated by A.C. and J.M. and the NIH facility. MR1 tetramers were coupled to streptavidin-PE (BD Bioscience). For intracellular staining, the cells were fixated and permeabilized using the Foxp3 staining kit from eBioscience according to the manufacturer’s instructions, cells were then stained with anti-Ki67 (SolA15), anti–Foxp3 (FJK-16s), anti-Ror(γ)t (B2D) from eBioscience; anti-BCL-2 (BCL/10C4), anti-PLZF (9E12 bioL), anti-T-bet (4B10) from Biolegend for 30 min at 4 °C. Intra-cytoplasmic staining for cytokine analysis were performed after PMA (25 ng/ml) and ionomycin (1 μg/ml) stimulation for 4 h at 37 °C in presence of Brefeldin A (10 μg/ml), all reagents from Sigma-Aldrich. After surface staining, cells were fixed and permeabilized with Cytofix/Cytoperm kit (BD Biosciences), washed, and incubated at 4 °C with antibodies to cytokines TNFα/ml), all reagents from eBioscience; IFNγ (XMG1.2), IL-17A (TC11-18H10) from Becton Dickinson. Data acquisition was performed using a BD Biosciences LSRFortessa cytometer, and cell sorting was performed using BD Biosciences FACSAria III, then the results were analyzed using FlowJo analysis software (Tree Star). Antibodies are listed in Table 1.

**Table 1:** List of antibodies and reagents.

Antibodies		
PerCP-Cy5.5 rat anti-mouse CD45 (30-F11) (1/100)	BD Biosciences	550994
APC/Cy7 anti-mouse CD45 (30-F11) (1/100)	Biolegend	103116
BD Horizon™ BV711 anti-mouse TCR β chain (H57) (1/100)	BD Biosciences	563135
BD Horizon™ BV421 anti-mouse TCR β chain (H57) (1/100)	BD Biosciences	562839
Brilliant Violet 605 anti-mouse CD8a (53-6.7) (1/100)	Biolegend	100743
Alexa Fluor 700 anti-mouse CD8α (53-6.7) (1/100)	eBioscience	56-0081-82
PE-Cyanine7 anti-mouse CD8α (53-6.7) (1/100)	eBioscience	25-0081-82
BD Horizon™ BV421 anti-mouse CD103 (M290) (1/100)	BD Biosciences	562771
Alexa Fluor 700 anti-mouse CD44 (IM7) (1/100)	eBioscience	56-0441-82
Brilliant Violet 785 anti-mouse CD45.1 (A20) (1/100)	Biolegend	110743
APC anti-mouse CD45.2 (104) (1/100)	Biolegend	109814
Brilliant Violet 711 anti-mouse Ly-6A/E (Sca-1) (D7) (1/100)	Biolegend	108131
Brilliant Violet 510 anti-mouse NK1.1 (PK136) (1/100)	Biolegend	108737
Brilliant Violet 650 anti-mouse CD19 (6D5) (1/100)	Biolegend	115541
Brilliant Violet 510 anti-mouse CD4 (GK1.5) (1/100)	Biolegend	100449
Brilliant Violet 785 anti-mouse/human CD11b (M1/70) (1/100)	Biolegend	101243
PE/Cy5 anti-mouse CD127 (IL-7Ralpha) (A7R34) (1/100)	Biolegend	135016
PerCP/Cy5.5 anti-mouse CD25 (PC61) (1/100)	Biolegend	102029
Brilliant Violet 605 anti-mouse CD69 (HI-2F3) (1/100)	Biolegend	104530
PE anti-mouse CD11c (N418) (1/100)	Biolegend	117308
PerCP-eFluor 710 anti-mouse CD170 (Siglec-F) (IRNM44N) (1/100)	eBioscience	46-1702-80
PE-Cyanine 7anti-mouse F4/80 (BM8) (1/100)	eBioscience	25-4801-82
APC anti-mouse TCRγδ (GL-3) (1/100)	eBioscience	17-5711-82
PE anti-mouse FOXP3 (FJK-16s) (1/100)	eBioscience	12-5773-82
PE-eFluor 610 anti-mouse ROR γE-e (Β2Δ) (1/100)	eBioscience	61-6981-80
PerCP/Cy5.5 anti-mouse PLZF (9E12) (1/100)	Biolegend	145807
Brilliant Violet 60 anti-mouse T-bet (4B10) (1/100)	Biolegend	644817
Brillant Viollet 450 anti-mouse Ki67 (SolA15) (1/100)	eBioscience	48569882
PE anti-mouse CD206 (C068C2) (1/100)	Biolegend	141706
PE-CF594 anti-mouse CX3CR1 (SA011F11) (1/100)	Biolegend	149014
Alexa Fluor 700 anti-mouse CD11c (N418) (1/100)	eBioscience	56-0114-82
Alexa Fluor 647 anti-mouse Bcl-2 (BCL/10C4) (1/100)	Biolegend	633509
eFluor450 anti-mouse TNFα (MP6-XT22) (1/100)	eBioscience	48-7321-82
Alexa Fluor 700 anti-mouse IFN-γ (XMG1.2) (1/100)	BD Biosciences	557998
PerCP-Cy5.5 anti-mouse IL-17A (TC11-18H10) (1/100)	BD Biosciences	560666
anti-Akt (polyclonal) (1/2000)	Cell signaling	9272S
anti-pAkt (polyclonal) (1/2000)	Cell signaling	9331S
BD Via-Probe™ cell viability solution (1/500)	BD Biosciences	555815
*Critical commercial assays*		
TRIGS	RANDOX	TR210
GLY	RANDOX	GY 105
Ultra-sensitive mouse insulin ELISA kit	Crystal Chem	90080
Lamina propria dissociation kit	Miltenyi	130-097-410

### MAIT cell transfer

MAIT cells were purified from Rorc(γt)-GFP Vα19 Tg Cα^−/−^ CD45.1 congenic B6 mice. These mice are highly enriched on MAIT cells. Cells were isolated and purified from the spleen and mesenteric lymph nodes using Dynabeads™ untouched™ mouse T cells kit according to the manufacturer’s instructions. After purification, 5 million cells in 100 μl of PBS 2% FCS were intravenously injected into CD45.2 B6 mice (lean or obese). Five days later, mice were sacrificed, and CD45.1 MAIT cells were analyzed by flow cytometry.

### RT-qPCR

RNA was extracted from tissues or purified cells using the RNeasy RNA Mini and Micro Kit (Qiagen RNeasy Plus micro kit ref# 74034 and Qiagen RNeasy mini Kit ref# 74104). Complementary DNAs were synthesized using Superscript III reverse-transcriptase kit (Invitrogen ref# 18080-044). Quantitative PCR analysis was performed with SYBR Green Master Mix (Roche ref# 001,04,352,887) using a LightCycler 480 (Roche). *18S*, *Hprt*, and *Gapdh* were used for normalization to quantify relative mRNA expression levels. Relative changes in mRNA expression were calculated using the comparative cycle method (2^−ΔΔCt^). Primers are listed in Table 2.

**Table 2:** qPCR primers.

Name	Forward	Reverse
*Atgl*	aggtcgacatgttcccgagggagaccaagtggaa	aggtcgactcagcaaggcgggaggccaggtggat
*Hsl*	tggcacaccattttgacctg	ttgcggttagaagccacatag
*Leptin*	gagacccctgtgtcggtc	ctgcgtgtgtgaaatgtcattg
*Adiponectin*	tgttcctcttaatcctgccca	cca-acc-tgc-aca-agt-tcc-ctt
*Ccl2*	gggcctgctgttcacagtt	ccagcctactcattgggat
*Ccl5*	ccctcaccatcatcctcact	ccttcgagtgacaaacacga
*Ccl20*	aagacagatggccgatgaag	accccagctgtgatcatttc
*Ccr2*	atccacggcatactatcaacatc	caaggctcaccatcatcgtag
*Ccr6*	tgggccatgctccctagaa	ggtgaggacaaagagtatgtctg
*Ccr9*	agg-cca-aga-agt-cat-cca-agc	cct-tcg-gaa-tct-ctc-gcc-aa
*Cd163*	ccttggaaacagagacaggc	tccacacgtccagaacagtc
*Cd206*	tgtggtgagctgaaaggtga	caggtgtgggctcaggtagt
*Cxcr6*	tctttggactgctaggaaactcc	agagtacagacaaacaccaggt
*Gzmb*	ggactgcaaagactggcttc	ataacattctcggggcactg
*Il1β*	cagcaggttatcatcatcatcc	atctcacagcagcacatcaac
*Il5*	tcaggggctagacatactgaag	ccaaggaactcttgcaggtaat
*Il6*	agttgccttcttgggactga	tccacgatttcccagagaac
*Il10*	ggttgccaagccttatcgga	acctgctccactgccttgct
*Il13*	agaccagactcccctgtgca	tgggtcctgtagatggcattg
*Il17a*	gctccagaaggccctcaga	agctttccctccgcattga
*Il18*	gaaaatggagacctggaatc	tgtcaacgaagagaacttgg
*Il18r*	cgtgacaagcagagatgttg	atgttgtcgtctccttcctg
*Il22*	caacttccagcagccataca	gttgagcacctgcttcatca
*Il23p19*	ataatgtgccccgtatccag	ctggaggagttggctgagtc
*Il25*	acagggacttgaatcgggtc	tggtaaagtgggacggagttg
*Ifnγ*	actggcaaaaggatggtgac	tgagctcattgaatgcttgg
*Ltα*	ccacctcttgagggtgcttg	catgtcggagaaaggcacgat
*Tgfβr2*	acattactctggagacggtttgc	agcggcatcttccagagtga
*Tnfα*	agcccccagtctgtatcctt	ctccctttgcagaactcagg
*Zonulin-1*	acccgaaactgatgctgtggatag	aaatggccgggcagaacttgtgta
*Claudin4*	cgctactcttgccattacg	actcagcacaccatgacttg
*Occludin*	atgtccggccgatgctctc	tttggctgctcttgggtctgtat
*Mucin2*	cccagaagggactgtgtatg	tgcagacacactgctcaca
*Bcl-2*	gtcgctaccgtcgtgacttc	cagacatgcacctacccagc
*cMyc*	atgcccctcaaggtgaacttc	cgcaacataggatggagagca
*Bax*	ccggcgaattggagatgaact	ccagcccatgatggttctgat
*Casp9*	gacgctctgctgagtcgag	ggtctaggggtttaacagcctc
*Gapdh*	aacgaccccttcattgac	tccacgacatactcagcac
*Hprt*	aagcttgctggtgaaaagga	ttgcgctcatcttaggcttt
*18S*	gtaacccgttgaaccccatt	ccatccaatcggtagtagcg
*Actinobacteria*	ccgtactccccaggcgggg	cgcggcctatcagcttgttg
*Bacteroidetes*	ggcgaccggcgcacggg	grcttcctctcagaaccc
*Firmicutes*	gcagtagggaatcttccg	attaccgcggctgctgg
*UTB*	actcctacgggaggcagcag	attaccgcggctgctgg

### Metabolic analysis

Glucose tolerance test: Oral glucose tolerance tests were performed after 10–12 weeks of diet as follows: overnight fasted mice were injected with glucose by gavage (2 g/kg body weight of glucose, 20% glucose solution; G8270, Sigma). Blood glucose concentration was determined at 0, 15, 30, 60, and 90 min following the glucose load with a glucometer (Accu-Chek^®^ Performa, Roche) on blood from the tip of the tail vein. Insulin tolerance test: intraperitoneal (i.p.) insulin tolerance tests were performed after 10–12 weeks of diet as follows: 6-h fasted mice were injected with 0.00075 U of insulin/g of mouse (0.075 U/mL in PBS 1% BSA solution; Humalog 100 UI/mL, Lispro, Lilly). Blood glucose concentration was determined at 0, 10, 15, 30, 60, and 120 min following the glucose load with a glucometer (Accu-Chek^®^ Performa, Roche) on blood from the tip of the tail vein. The area under the curve (AUC, 0-90 or 120 min) was calculated for each group of mice.

Plasma triglycerides and glycerol release were measured using the colorimetric diagnostic kit, according to the manufacturer’s instructions (Randox Laboratories).

For insulin-signaling assays, mice were fasted for 6 h and then i.p. injected with 1.00 U/kg insulin. Mice were euthanized after 15 min, and liver, Epi-AT, and muscle were collected for western blot analysis.

Lean tissue and fat mass were measured using an Echo Medical systems’ EchoMRI 100 (Whole Body Composition Analyzers, EchoMRI, Houston, USA), according to the manufacturer’s instructions.

### In vivo analysis of intestinal permeability

In vivo intestinal permeability was evaluated by the intestinal permeability of FITC-dextran 4 kDa. Briefly, 6-h water-fasted mice were gavaged with FITC-dextran 4 kDa by gavage (600 mg/kg body weight, 120 mg/ml; Sigma-Aldrich, St Louis, MO). After 4 h, 120 μl of blood were collected from each mouse from the retro-orbital vein. The blood was centrifuged at 4 °C, 10,000 rpm, for 5 min. Plasma was analyzed for FITC-dextran 4 kDa concentration with a fluorescence spectrophotometer (SPARK 10M, TECAN) at excitation and emission wavelengths of 485 nm and 535 nm, respectively. Standard curves for calculating the FITC-dextran 4 kDa concentration in the samples were obtained by diluting FITC-dextran 4 kDa in PBS.

### Western blot

Samples were lysed in RIPA buffer (Sigma ref# R0278-50ML) supplemented with protease (Sigma ref# S8820-2TAB) and phosphatase (Roche ref# 001,04,845,906) inhibitors, and were diluted to a concentration of 20 μg of protein and heated at 98 °C for 10 min. Proteins were separated by SDS–PAGE electrophoresis and transferred to nitrocellulose membranes (Bio-Rad ref# 1620168). Blocking reagent (TBS with 0.5% Tween 20 and 3% BSA, pH 7.4) were incubated for 1 h, and primary antibody was incubated overnight at 4 °C in the blocking solution. The antibodies and their concentrations are the following: anti-phospho-AKT (Cell signaling, 9331S; 1:2000), anti-AKT (Cell signaling, 9272S; 1:2000). After several washes in PBS with 0.5% Tween 20, horseradish peroxidase (HRP)-labeled secondary antibodies (1:5000) were incubated for 1 h at room temperature in the blocking solution. Membranes were incubated with ECL western-blotting substrate (Bio-Rad ref# 170–5060) and imaged by the myECL Imager (ThermoFisher, 62236). Blots were semi-quantified using ImageJ software.

### MAIT cell and macrophage co-culture

MAIT cells were isolated from the spleen of Vα19 Tg Cα^−/−^ mice as described above. In some experiments, MAIT cells were pre-activated O.N. using Dynabeads Mouse T-Activator CD3/CD28 (Gibco, 11453D) following the manufacturer instructions. BMDMs were isolated from WT or Mr1^−/−^ C57BL6/J male mice. Mice were euthanized, and the bone marrow from their femur and tibias was collected. BMDMs (2 × 10^5^ cells/well) were cultured and differentiated for 7–10 days, in RPMI 1640 medium (Gibco, 6187-010) supplemented with 10% FCS, 1% penicillin/streptomycin, and 50 ng/ml MCSF (Miltenyi Biotec, 130-101-706). M0 macrophages were co-cultured with activated or non-activated MAIT cells (2 × 10^5^ cells/well) for 72 h prior to FACS analysis. In some experiments, 10 μg/ml of cytokine-neutralizing antibodies (anti-IL-17, anti-IFNγ, or anti-TNFα) were added to the culture media. In another set of experiments, in vitro-differentiated macrophages were either kept in their M0 state or further differentiated for 48 h into M1 macrophages by replacing MCSF by 25 ng/ml of recombinant IFNγ (Miltenyi Biotec, 130-105-785) or M2 macrophages by replacing MCSF by 20 ng/ml of recombinant IL-4 (Miltenyi Biotec, 130-097-757). Differentiation of BMDM into M0, M1, or M2 macrophages was similar in WT and Mr1^−/−^ mice as validated by qPCR of characteristic genes. M0, M1, and M2 macrophages were co-cultured with purified MAIT cells for 48 h prior to FACS analysis.

### MAIT cell ligands in cecum contents

Cecum contents were recovered, weighted, and half of each sample was resuspended in PBS (the other half was kept for the metagenomic analyses). After homogenization, supernatants were centrifuged at 14,000 rpm for 5 min at 4 °C and were passed through a 0.22-μm filter. Supernatants were tested at final concentration of 1.5 mg intestinal content per ml, and serial dilutions of 5-OP-RU were used as a positive control. Two bioassays were performed in Cochin Institute and Melbourne University, respectively. In Cochin Institute, WT3-MR1 cell line (mycoplasma free) was used to detect MAIT cell ligand from intestinal contents^56^. Two hours before the experiment, WT3-MR1 cells were coated onto a 96-flat-well plate at final concentration of 0.5 × 10^6^ per ml in medium containing DMEM Glutamax 10% FCS, 1 M HEPES; 100 mM sodium pyruvate, 1% nonessential amino acids and 1% penicillin–streptomycin. MAIT cells were isolated from the spleen of Vα19 Tg Cα^−/−^ mice as described above and were co-cultured with WT3-MR1 cells at final concentration of 5 ×  10^5^ MAIT cells per ml. Acetyl-6-FP (10 μM) was added to determine MR1-specific activation. One day later, MAIT cells were stained with the appropriate antibodies, and activation was analyzed by flow cytometry.

The second bioassay is based on the activation of Jurkat.MAIT cells. MAIT cell reporter activation and MR1 surface expression upregulation assays were performed essentially as previously reported^34,71^. Jurkat cells overexpressing the MAIT TCR clone AF-7 (Jurkat.MAIT), or an HLA-B8-EBV peptide-specific non-MAIT control TCR (Jurkat.LC13) were tested for activation by co-incubation with 10 μl colonic extracts from individual mice, or 0.1 nM 5-OP-RU (positive control) and C1R cells overexpressing MR1 (CIR.MR1) for ~16 h. Cells were subsequently stained with PE-Cy7-conjugated anti-CD3 (UCHT1, eBioscience, 1:300), APC-conjugated anti-CD69 (BD, 1:25), and 7-aminoactinomycin D (7AAD) before analysis by on a FACS CantoII (BD) flow cytometer. Activation of Jurkat.MAIT cells (defined as CD3^+^, 7AAD^−^, and separated from C1R.MR1 cells based on GFP expression) was measured by an increase in surface CD69 expression. Jurkat.LC13 cells co-cultured with C1R cells expressing HLA-B8 in the presence of the Epstein–Barr viral peptide FLRGRAYGL (FLR) was included as a positive control for their activation. For blocking, C1R.MR1 cells were first incubated with anti-MR1 mAb 26.5^56^ or isotype control mAb W6/32^72^, both purified in house, at 20 μg/ml for 1 h, prior to addition of Jurkat cells and test samples.

### Neonatal transfer of microbiota

Weaned B6 males (aged 21–26 days) were gavage with diluted fecal contents from adult 11–20-week-old males, according to a previous study^73^ with slight modifications. Briefly, the fresh feces from adult donor mice were collected in a sterile tube, and the contents were mixed to 50 volumes of sterile water, then 250 μL of this suspension was given to each recipient mouse by oral gavage using a 24-G round-tip gavage needle. Recipients were rested for 24 h, and this procedure was repeated once. Moreover, to strengthen transfer of microbiota efficacy, we gave “fecal water”, from the same donor groups to the same recipient groups, twice a week during 8–10 weeks.

### Ac-6-FP treatment

Vα19^+/−^ mice fed during 6 weeks of HFD were then giving water ad libitum containing 50 nM/ml of Ac-6-FP (Acetyl-6-formylpterin) or PBS for the following 8 weeks of HFD and intraperitoneally injected with a total volume of 200 μl containing 50 nM of Ac-6-FP or 200 μl of PBS, twice a week. ITT and OGTT were performed 6 weeks after the beginning of the treatment. At 8 weeks of treatment, mice were scarified prior to analysis.

### Statistical analysis

All statistical tests were performed using GraphPad Prism 8.3.0 (GraphPad Software, Inc., La Jolla, CA), and all data were represented as mean ± S.E.M. Statistical tests were assessed after confirming that the data met appropriate assumptions (normality, homogenous variance, and independent sampling). Gaussian distribution was tested using the Kolmogorov–Smirnov test. Group comparisons were assessed with Mann–Whitney, Student’s *t* or ANOVA tests to compare groups. All statistical tests were two-tailed, and *P* < 0.05 was defined as significant.

### Histology

Epi-AT and ileum samples were fixed in 3% formaldehyde solution overnight and embedded in paraffin. Epi-AT slides were stained with hematoxylin and eosin for the evaluation of the tissue morphology, following standardized protocols. Adipocyte size was measured by the diameters of the adipocytes in light-microscopy images (×20) of Epi-AT sections ( = 50 adipocytes per section, three sections per mouse, six mice per group), and analyzed using ImageJ software.

### Bacteria 16S sequencing and bioinformatics analysis

16S sequencing analyses were performed on feces. Read pairs were quality filtered, adapter-trimmed, and merged using Flash1.6.2^74^. PCR primers were then removed, and sequences with sequencing errors were excluded (Mothur)^75^. In total, 15000 reads were randomly selected for each sample. Merged read pairs were demultiplexed using QIIME, and clustered into operational taxonomic units (OTUs). Chimera were removed using UCHIME^76^ and Mothur^75^ softwares. A sequence similarity threshold of 97% was used to assign reads to OTUs against the Greengenes database (release 13-5)^77^ and RDP classifier^78^.

Following rarefaction of all communities to even sampling depths, the abundances of all families were computed by agglomerating the OTUs assigned to those families. For each family, Mann–Whitney test with BH correction^79^ were carried out to identify the combinations (treatment) that were significantly different in terms of abundance. The same method was used for each genus. All analyses were done using R package (R Core Team, 2015, R Foundation for Statistical Computing, Vienna, Austria). Statistical analyses were performed using non-parametric Mann–Whitney *U* test. The results were considered statistically significant when *P*-values were lower than 0.05.

### Metagenomic analysis

Metagenomic analyses were performed on cecum content from the same samples used for the bioassays. A set of 18 KEGG modules was identified within the “Cofactor and vitamin biosynthesis” category. Abundance of each KEGG module in the sample was calculated as the number of read pairs mapping to all genes annotated to a given KEGG module, divided by the total number of mapped read pairs. Further, KEGG Orthologs (KOs) involved in menaquinone, NAD, tetrahydrofolate, and riboflavin biosynthesis were extracted. KEGG Ortholog abundance is defined as the number of read pairs mapping to all genes annotated to a given KEGG ortholog, divided by the total number of mapped read pairs. Statistical comparisons between groups were performed using two-sided Wilcoxon rank-sum test. Where multiple hypotheses were evaluated in parallel, the Benjamini–Hochberg method was used to control false discovery rate.

### Reporting summary

Further information on research design is available in the Nature Research Life Sciences Reporting Summary linked to this article.

## Supplementary information

Supplementary Information

Reporting Summary

## Data Availability

The data that support the findings of this study are available in figshare with the identifier doi: 10.6084/m9.figshare.12490271 and in the Source Data file. All data are available from the corresponding authors upon reasonable request. Source data are provided with this paper.

## Source data

Source Data
