# Supplementary material for: Mucosal-associated invariant T cells promote inflammation and intestinal dysbiosis leading to metabolic dysfunction during obesity
Source: Nat Commun. 2020 Jul 24;11:3755. doi: 10.1038/s41467-020-17307-0 (PMC7381641; doi:10.1038/s41467-020-17307-0)
Supplement: Supplementary file 1 — Supplementary Information [file 41467_2020_17307_MOESM1_ESM.pdf]

Supplementary Information

Mucosal-Associated Invariant T cells promote inflammation and intestinal dysbiosis leading to metabolic dysfunction during obesity

Toubal et al.

This file contains:

Supplementary Figures 1-16

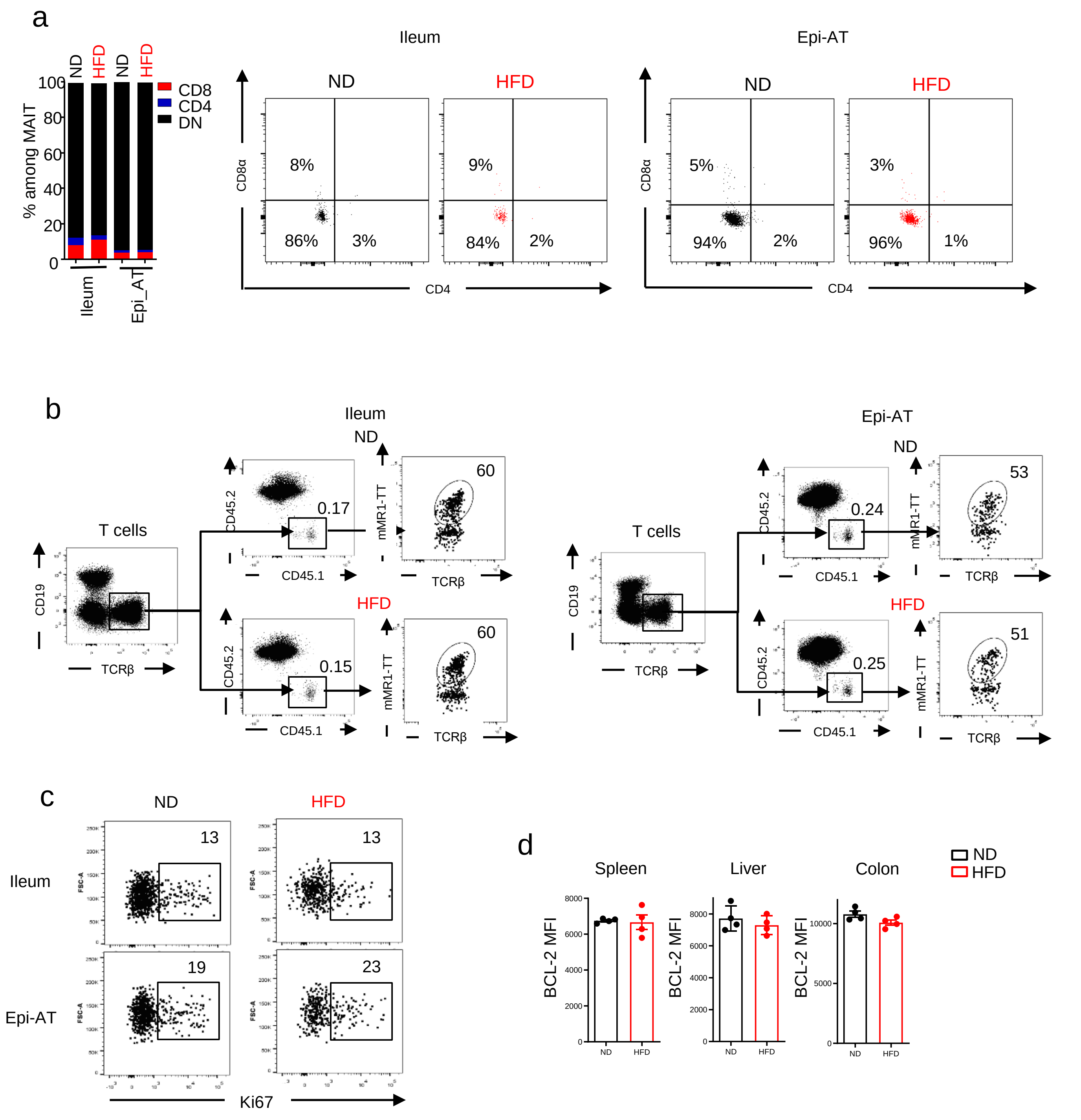

Supplementary Figure 1 related to Figure 1: Impact of obesity on tissue MAIT cells

(a) CD4 and CD8expression by MAIT cells in the ileum and Epi-AT, from ND and HFD fed mice and representative dot plots. (b) Migration of MAIT cells into lean or obese (12 weeks of ND or HFD) recipient B6 mice. Purified MAIT cells from CD45.1 Vα19 transgenic $C\alpha^{-/-}$ B6 mice were transferred into CD45.2 B6 mice, and analyzed 5 days later. Representative dot plots of MAIT cell staining and the numbers represent the frequency of CD45.1 T cells cells and MAIT cells among αβT cells. (c) Representative dot plot of intracellular staining of Ki67 in MAIT cells. (d) BCL-2 mean fluorescence intensity (MFI) in MAIT cells from spleen, liver and colon from mice fed ND (n=4) or HFD (n=4) for 12 weeks. In d data are represented as mean ± SEM..

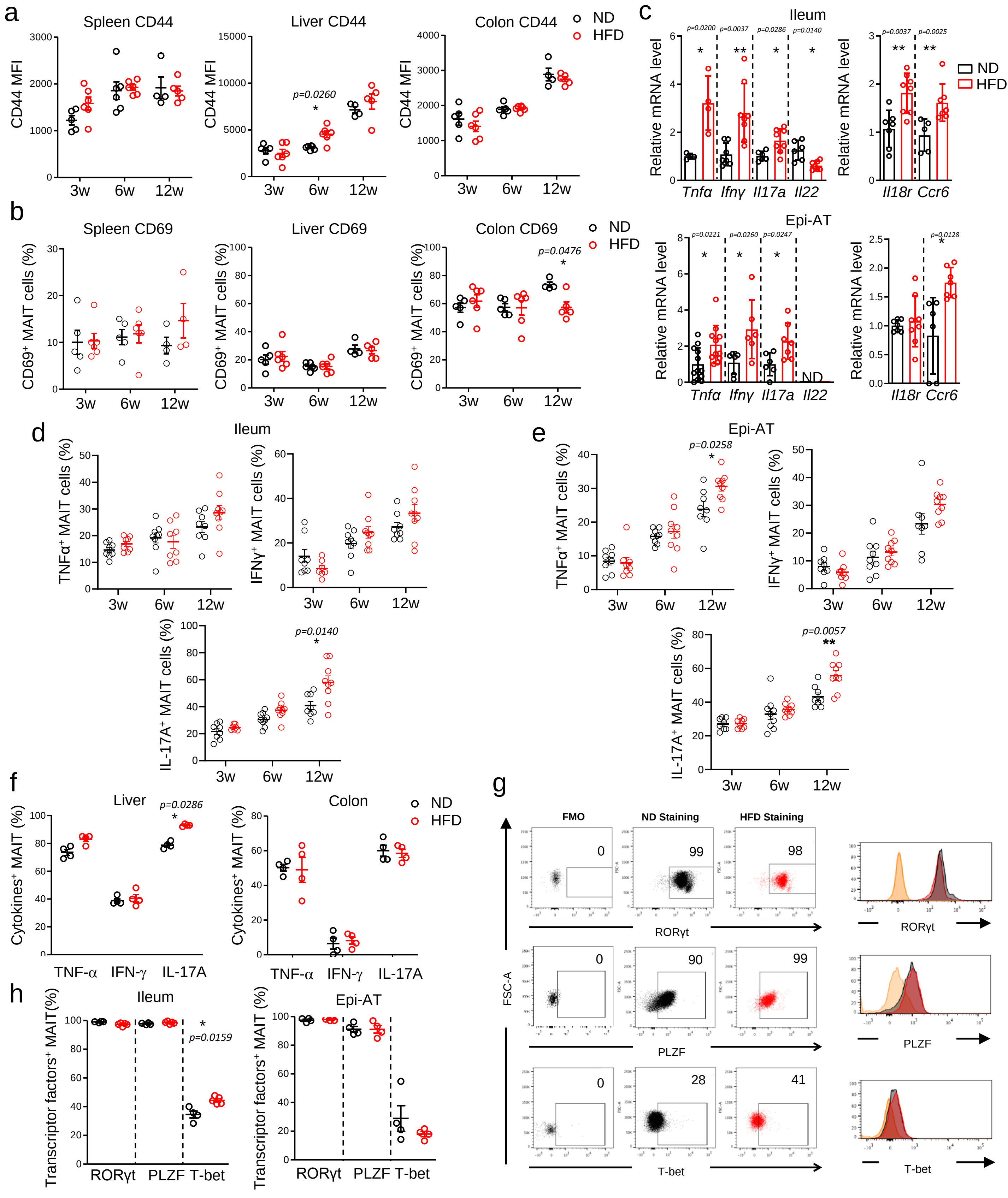

Supplementary Figure 2 related to Figure 2 : Impact of obesity on tissue MAIT cell phenotype and cytokine production

(a-b) Graphs representing CD44 mean fluorescence intensity (MFI) (a) and % of CD69+ MAIT cells among total MAIT cells from spleen, liver, colon (b) in mice fed with HFD for 3, 6 and 12 weeks (n=5 per group). (c) FACS-sorted MAIT cells from ileum and Epi-AT of obese (n=8) and lean (n=7) mice were analyzed, by qPCR, for their expression of genes coding for cytokines (*Tnfα, Ifnγ, Il17a and Il22*) and cytokine/chemokine receptors (*Il18r* and *Ccr6*) (d and e) Graphs representing intra-cellular staining of MAIT cells for TNFα, IFNγ, and IL-17A. Frequency of positive MAIT cells from ileum (n=8) (d) and Epi-AT (n=8) (e) in mice fed with HFD for 3, 6, 12 and 16 weeks. (f) Intra-cellular staining for TNF-α, IFN-γ and IL-17A in MAIT cells from colon and liver of lean (n=4) and obese (n=4) mice fed during 12 weeks. (g) Intracellular staining of PLZF, RORγt and T-bet in MAIT cells of obese (12 weeks HFD) or lean mice. FMO controls are used to assess the specificity of the staining.(h) Intra-cellular staining of MAIT cells, for RORγt, PLZF and T-bet transcription factors. Frequency of positive MAIT cells from the ileum and Epi-AT of lean (n=4) and obese (n=4) mice are shown For a, b, d, e, f and h each symbol represents an individual mouse (small horizontal lines indicate the mean ± S.E.M.). In c, data are represented as mean ± S.E.M.. All statistical analysis were performed by two-tailed Mann-Withney test. *P<0,05 **P<0,01 ***P<0,001.

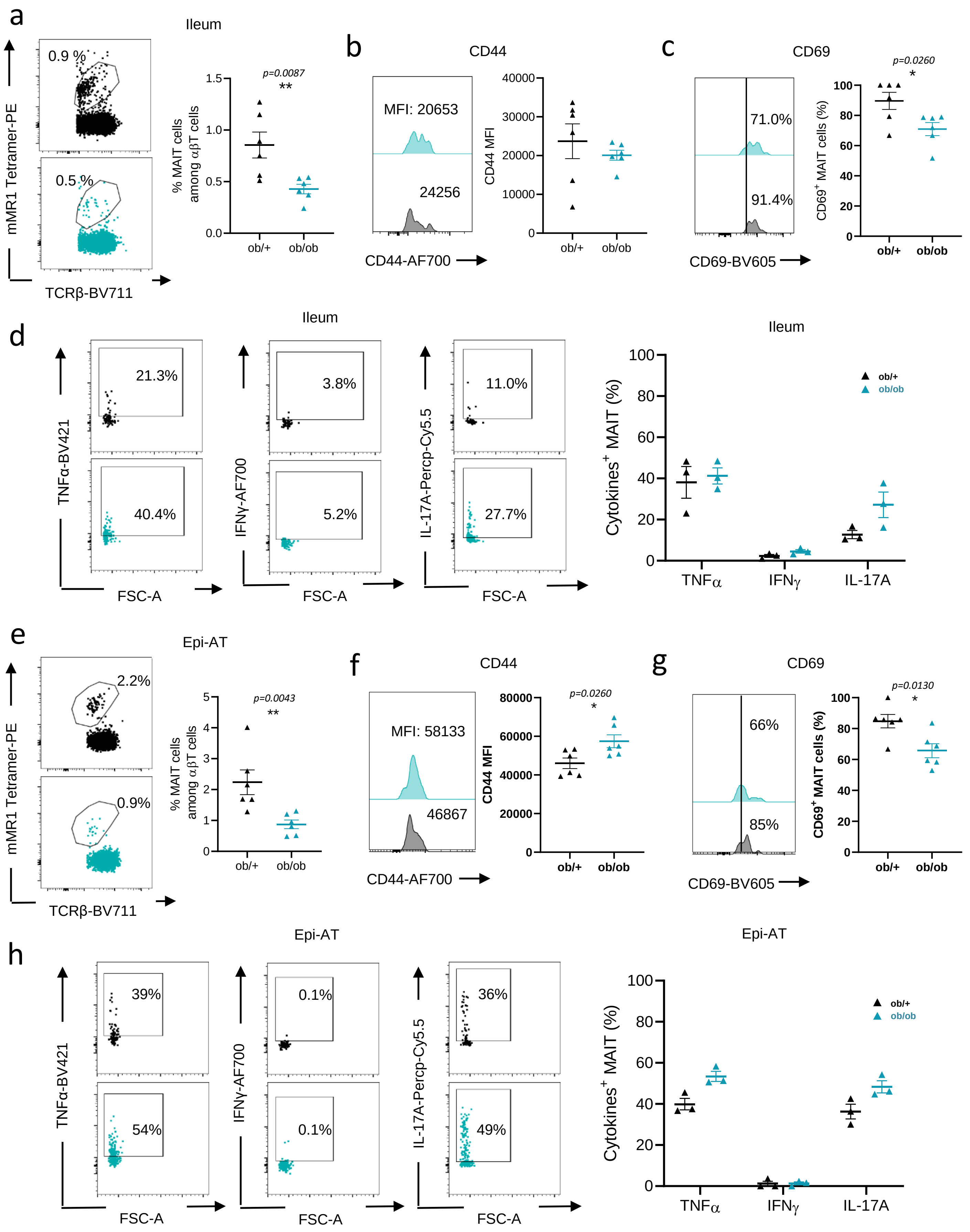

Supplementary Figure 3 related to figure 1 and 2: MAIT cell alterations in obese leptin deficient (ob/ob) mice

MAIT cells frequency and phenotype was analyzed by flow cytometry. MAIT cells in ileum and Epi-AT from leptin deficient ob/ob mice and their lean ob/+ controls were identified as CD45+CD19-CD11b-TCRγδ- and mouse-MR1 tetramers (loaded with 5–OP-RU) positive with low TCRβ staining. (a) Representative dot plot of MAIT cells in ileum (n=6 mice per group). The numbers represent the frequency of MAIT cells among αβT cells. (b-c) Graphs representing CD44 mean fluorescence intensity (MFI) (b) and % of CD69+ MAIT cells (c) among total MAIT cells from ileum of ob/ob (n=6) and ob/+ (n=6) mice. (d) Intra-cellular staining of ileum MAIT cells (n=3 mice per group) for TNFα, IFNγ and IL-17A. Frequency of positive cells are indicated. (e) Representative dot plot of MAIT cells from Epi-AT (n=6 mice per group). The numbers represent the frequency of MAIT cells among αβT cells. (f-g) Graphs representing CD44 mean fluorescence intensity (MFI) (f) and % of CD69+MAIT cells (g) among total MAIT cells from Epi-AT of ob/ob (n=6) and ob/+ (n=6) mice. (h) Intra-cellular staining of Epi-AT MAIT cells (n=3 mice per group) for TNFα, IFNγ and IL-17A. Frequency of positive cells are indicated. Each symbol represents an individual mouse (a, b, c, e, f, g) or a pool of two mice (d, h) and small horizontal lines indicate the mean ± SEM. Statistical analysis were performed by two-tailed Mann-Whitney test, *P<0.05 **P<0.01.

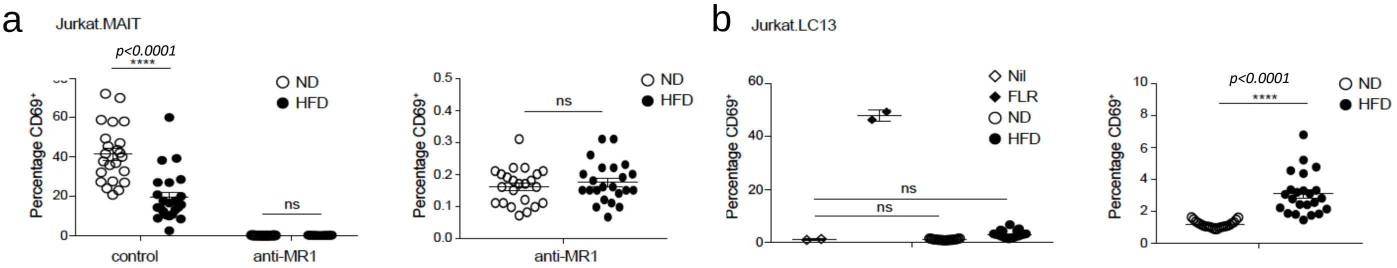

c

Description	Mean (Normal diet)	Mean High fat diet)	p-value	FDR
C1-unit interconversion	37.42	60.04	1.45×10-11	1.31×10-10
Pyridoxal biosynthesis	0.27	0.03	1.45×10-11	1.31×10-10
Tetrahydrofolate biosynthesis	58.00	35.07	9.92×10-9	5.95×10-8
Heme biosynthesis	17.43	27.68	2.32×10-8	1.04×10-7
Menaquinone biosynthesis	27.97	11.08	1.06×10-7	3.82×10-7
Biotin biosynthesis	10.19	18.26	3.29×10-7	8.14×10-7
Biotin biosynthesis	10.08	18.07	3.29×10-7	8.14×10-7
Biotin biosynthesis	10.79	18.44	4.07×10-7	8.14×10-7
NAD biosynthesis	56.24	36.04	4.07×10-7	8.14×10-7
Cobalamin biosynthesis	27.61	35.90	7.57×10-7	1.36×10-6
Glutathione biosynthesis	0.21	0.05	9.28×10-7	1.52×10-6
**Riboflavin biosynthesis**	**53.37**	**44.18**	**0.0002**	**0.0003**
Pantothenate biosynthesis	3.15	5.92	0.0004	0.0005
Thiamine Biosynthesis	36.66	31.5	0.0009	0.001
Ascorbate degradation	1.84	1.00	0.001	0.002
CoenzymeA synthesis	63.50	58.94	0.03	0.03

d

Gene	Function	Mean (Normal diet)	Mean (High fat diet)	Fold change	Mean difference	p-value	prevalence (Normal diet)	prevalence (High fat diet)
rib B	3,4 DHBP synthase	8,14E-07	**6,13E-06**	7,54E+00	5,32E-06	9,72E-10	2,00E+01	2,00E+01
ribH	Lumazine synthase	1,12E-04	**7,41E-05**	6,62E-01	-3,78E-05	1,41E-09	2,00E+01	2,00E+01
rib C / *rib* E	Riboflavine synthase	1,33E-04	**8,98E-05**	6,77E-01	-4,29E-05	2,32E-08	2,00E+01	2,00E+01
ribD2	Pyrimidine reductase	4,98E-05	**2,23E-05**	4,48E-01	-2,75E-05	3,29E-07	2,00E+01	2,00E+01
ribBA	GTP cyclohydrolase 2	2,32E-04	**1,72E-04**	7,44E-01	-5,94E-05	2,00E-06	2,00E+01	2,00E+01
ribD	Pyrimidine deaminase/reductase	2,05E-04	**1,68E-04**	8,17E-01	-3,75E-05	2,28E-04	2,00E+01	2,00E+01
ribF	iboflavin kinase/FMNadenylyltransferas	3,10E-04	2,96E-04	9,56E-01	-1,35E-05	5,96E-02	2,00E+01	2,00E+01
ribA	GTP cyclohydrolase	1,14E-06	9,31E-07	8,16E-01	-2,10E-07	2,42E-01	2,00E+01	1,90E+01

Supplementary Figure 4 related to Figure 2 Impact of obesity on MAIT cell ligands

(a-b) Activation of Jurkat.MAIT reporter cell (a), and JURKAT.LC13 control cells (b), detected by CD69 expression, by colonic extracts from mice fed on normal diet (ND, n=23) or high fat diet (HFD, n=23), for 16h of co-culture with CR.MR1 cells as antigen presenting cells. Data are represented as mean ± SEM from individual mice (pooled from 5 independent experiments) or for controls (Nil, 0,1nM 5-OP-RU or 60ug/ml FLR; Epstein-Barr viral peptide FLRGRAYGL in co-culture with CR.B8 cells) two duplicate wells. In a-b ****; p<0.0001, 2-way ANOVA with Sidak's multiple comparisons. ns; non-significant, Mann Whitney test. ****; p<0.0001, one-way ANOVA with Tukey's multiple comparisons. ****;p<0.0001 Mann Whitney test. (c) Gene enrichment analysis of the top up-regulated and down-regulated pathways after metagenomics analysis of cecal content of HFD (n=20) or ND (n=20) fed mice.(d) KEGG Orthologs involved in riboflavin biosynthesis.

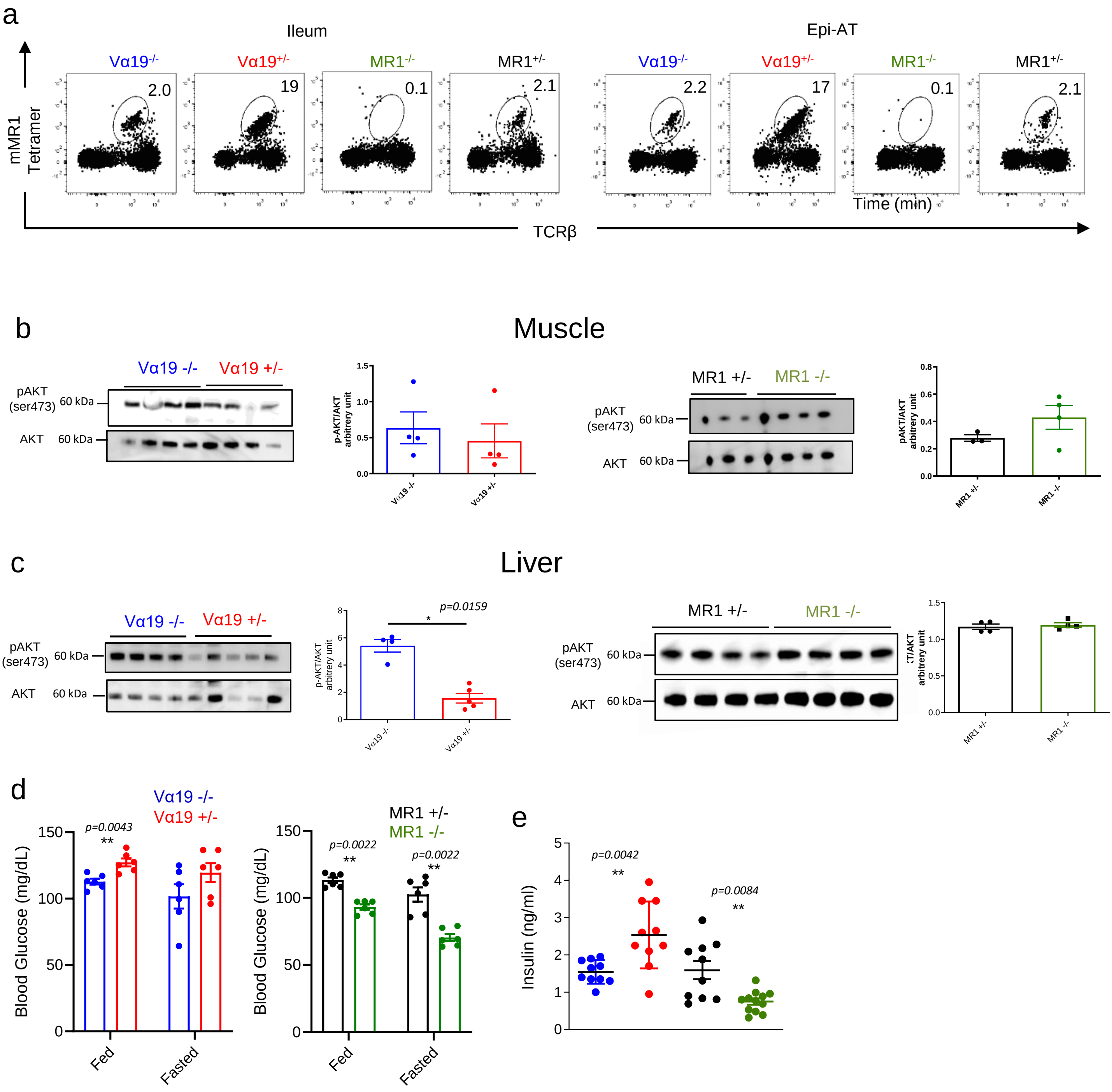

Supplementary Figure 5 related to Figure 3: MAIT cells induce metabolic dysfunction during obesity

(a) Representative dot plot showing MAIT cell frequency in ileum and Epi-AT of Vα19$^{+/-}$, MR1$^{-/-}$ mice and their respective littermate controls Vα19$^{-/-}$ (n=4) and MR1$^{+/-}$ (n=4) mice. (b-c) Western blot and quantification of basal AKT phosphorylation (p-AKT-S473) in muscle (b) and liver (c) of HFD-fed Vα19$^{+/-}$, MR1$^{-/-}$ mice and their littermate (n= 4 mice per group). (d) Blood glucose in fed and fasted mice (n=6 per group) (e) Basal insulin in Vα19$^{+/-}$ (n=10) and MR1$^{-/-}$ (n=12) mice and their littermate controls (Vα19$^{-/-}$ (n=10) and MR1$^{+/-}$ (n=10)) fed with HFD during 12 weeks. For e each symbol represents an individual mouse (small horizontal lines indicate the mean ± S.E.M.). In b-d, data are represented as mean ± S.E.M.. All statistical analysis were performed by two-tailed Mann-Withney test. *P<0,05 **P<0,01 ***P<0,001. (See also Supplementary Figure 2)

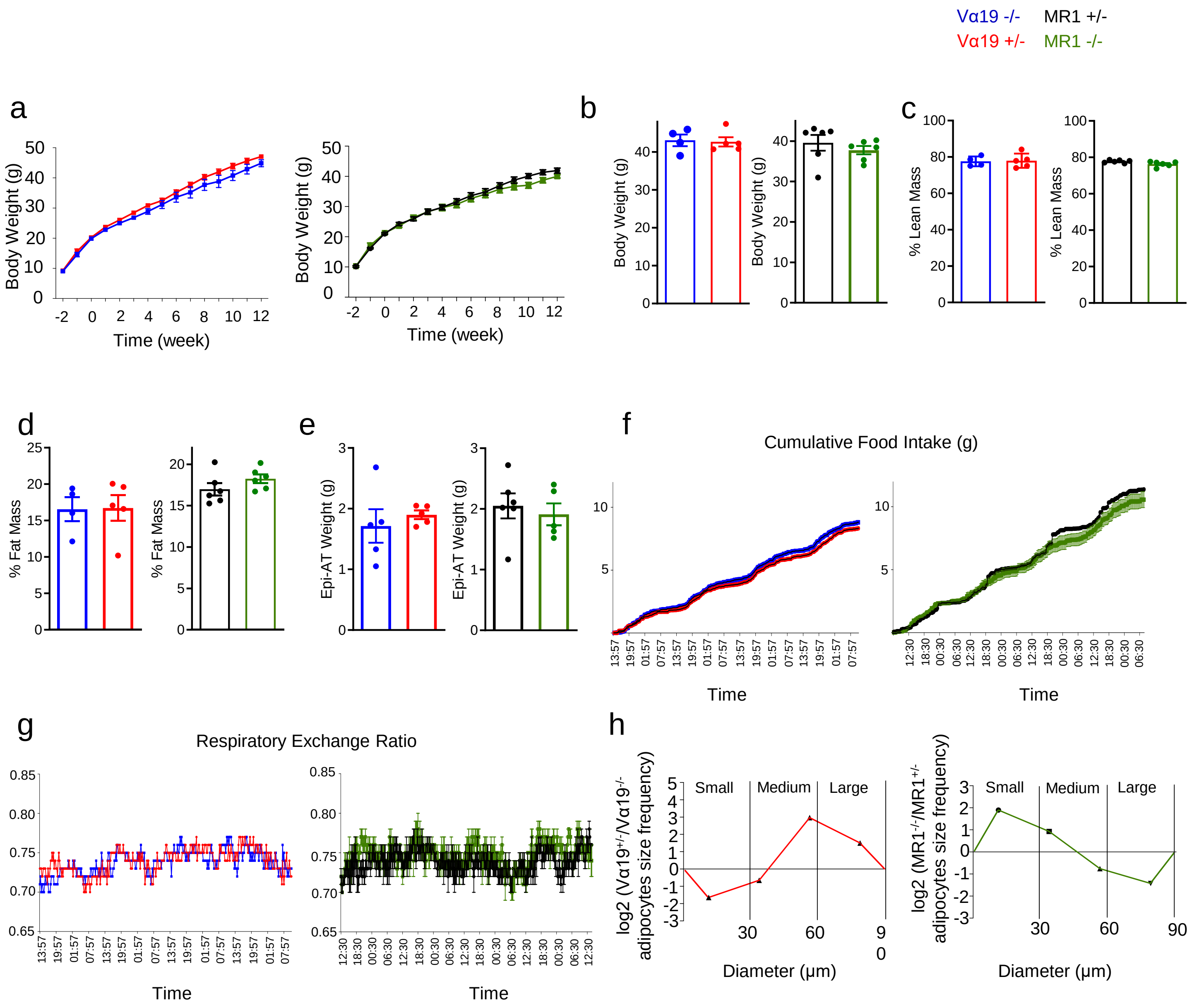

Supplementary Figure 6 related to Figure 3: MAIT cells induce adipose tissue modulation and metabolic disorder independently of food intake or activity

(a-b) Weight gain and body weight of Vα19$^{+/-}$ (n=5) and MR1$^{-/-}$ (n=6) HFD-fed mice and their littermate controls (Vα19$^{-/-}$ (n=4) and MR1$^{+/-}$ (n=6)) during 12 weeks of HFD. (c-d) Percentage of lean and fat mass in Vα19$^{+/-}$ (n=5) and MR1$^{-/-}$ (n=6) HFD-fed mice and their littermate controls (Vα19$^{-/-}$ (n=4) and MR1$^{+/-}$ (n=6)). (e) Epi-AT weight of Vα19$^{+/-}$ and MR1$^{-/-}$ HFD-fed mice and their littermate controls (n=6 per group). (f-g) Comparison of food intake and the respiratory exchange ratio (RER) between Vα19$^{+/-}$ (n=5) and MR1$^{-/-}$ (n=6) HFD-fed mice and their littermate controls (Vα19$^{-/-}$ (n=4) and MR1$^{+/-}$ (n=6)). (h) Quantification of adipocyte size in Epi-AT of Vα19$^{+/-}$ and MR1$^{-/-}$ HFD-fed mice and their littermate controls. Data are represented as mean ± S.E.M.. All statistical analysis were performed by two-tailed Mann-Withney test.

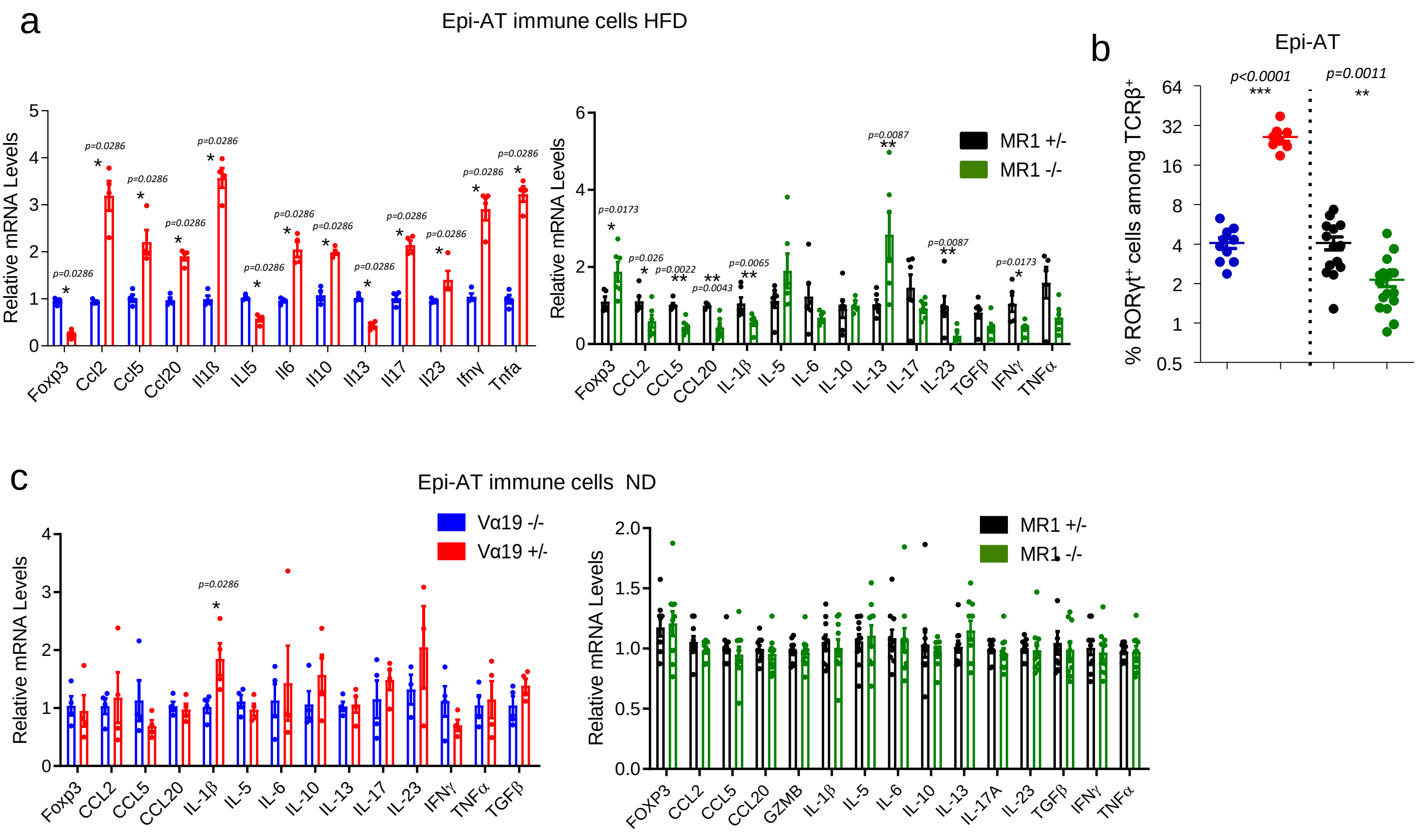

Supplementary Figure 7 related to Figure 4: MAIT cells induce Epi-AT immune system inflammation during obesity.
(a) Graph showing the relative expression of pro- and anti-inflammatory cytokine-transcripts in Epi-AT immune cells of Vα19$^{+/-}$ (n=4) and MR1$^{-/-}$ (n=6) mice and their littermate controls (Vα19$^{-/-}$ (n=4) and MR1$^{+/-}$ (n=6)) fed with HFD. (b) Frequency of RORγt^{+} cells among αβT cells (Th17) in Epi-AT immune cells of Vα19$^{+/-}$ (n=8), Vα19$^{-/-}$ (n=10), MR1$^{-/-}$ (n=17) and MR1$^{+/-}$ (n=14). (c) Graph showing the relative quantity of pro- and anti-inflammatory cytokine transcripts in Epi-AT immune cells of Vα19$^{+/-}$ (n=4) and MR1$^{-/-}$ (n=10) mice and their littermate controls (Vα19$^{-/-}$ (n=4) and MR1$^{+/-}$ (n=10)) fed with ND. For b each symbol represents an individual mouse (small horizontal lines indicate the mean ± S.E.M.). In a and c, data are represented as mean ± S.E.M.. All statistical analysis were performed by two-tailed Mann-Withney test. *P<0,05 **P<0,01 ***P<0,001.

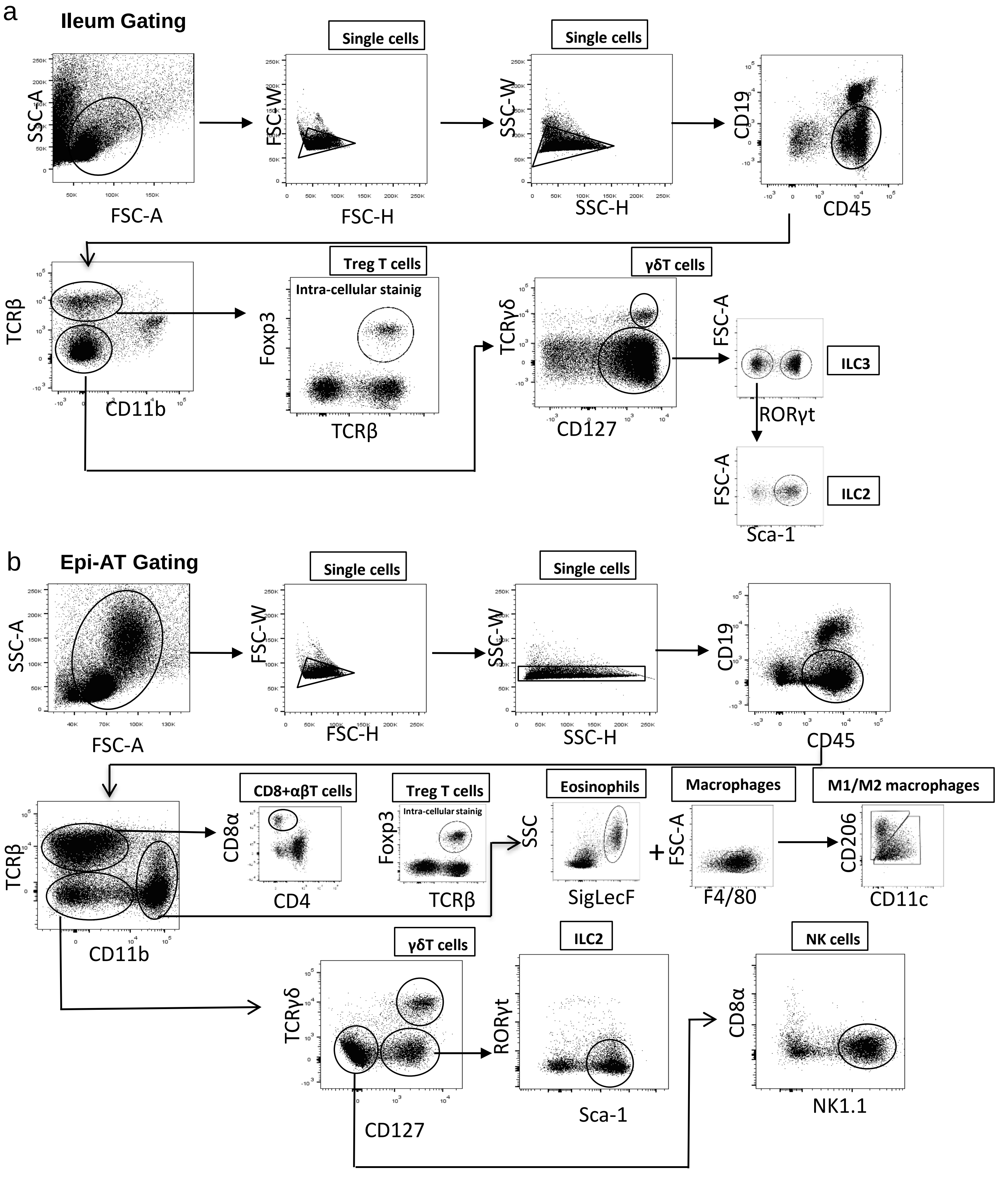

Supplementary Figure 8 related to Figure 4: MAIT cells impact other immune cells in ileum and Epi-AT of obese mice
(a) Representative Gating strategy in the ileum. (b) Representative Gating strategy in Epi-AT.

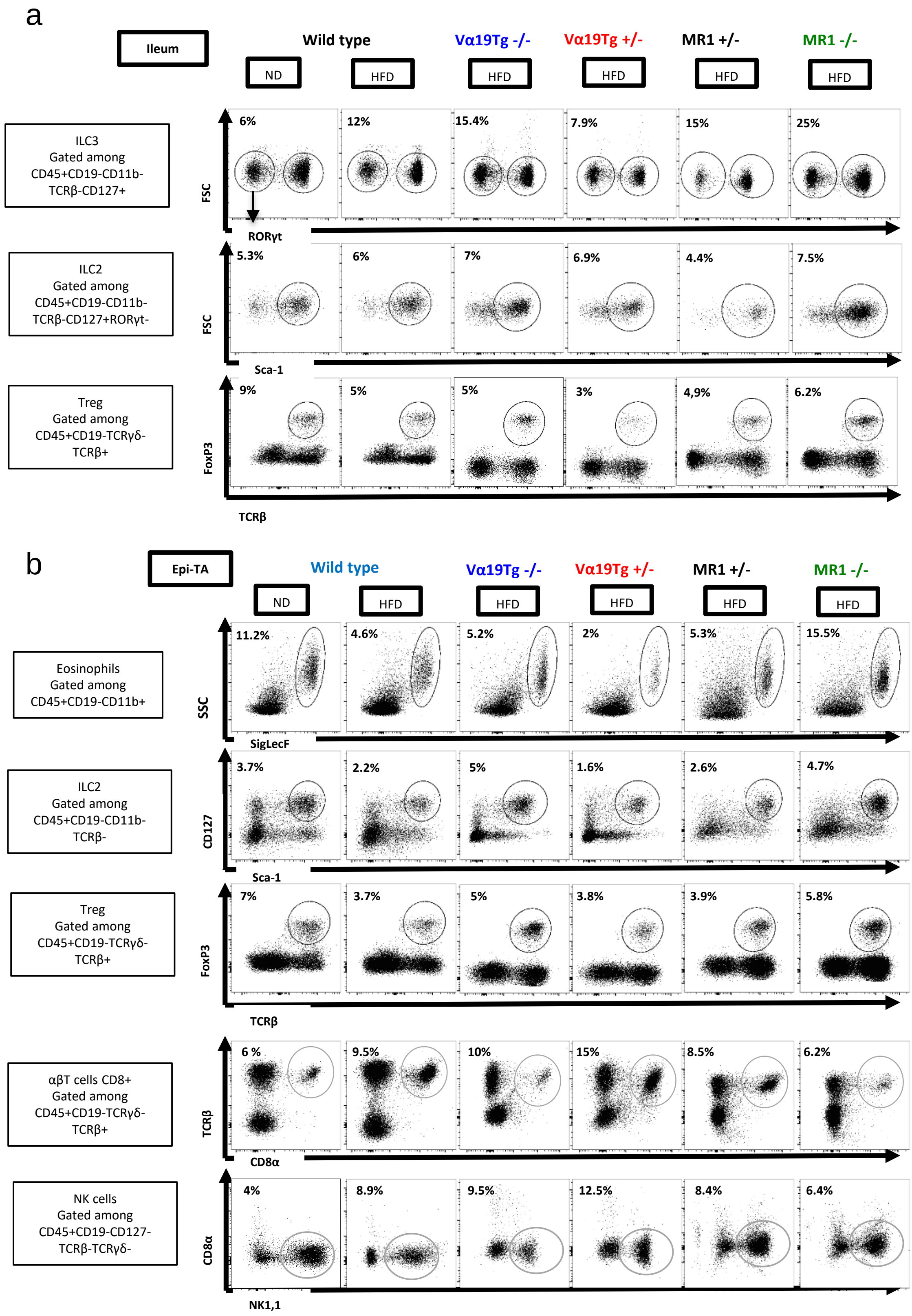

Supplementary Figure 9 related to Figure 4: MAIT cells impact other immune cells in the ileum and Epi-AT of obese mice

(a) Dot plots representation showing the frequency of Foxp3+ Treg cells, ILC2 and ILC3 among CD45^{+} cells in the ileum lamina propria of B6 mice fed with HFD or ND, Vα19$^{+/-}$ and MR1$^{-/-}$ mice and their littermate controls after 12 weeks of HFD or ND feeding. (b) Dot plots representation showing the frequency of Foxp3+ Treg cells, ILC2, eosinophils, CD8 αβT cells, NK cells and macrophages among CD45^{+} cells in the Epi-AT stroma-vascular fraction (SVF) of B6 mice fed with HFD or ND, Vα19$^{+/-}$ and MR1$^{-/-}$ mice and their littermate controls after 12 weeks of HFD or ND feeding.

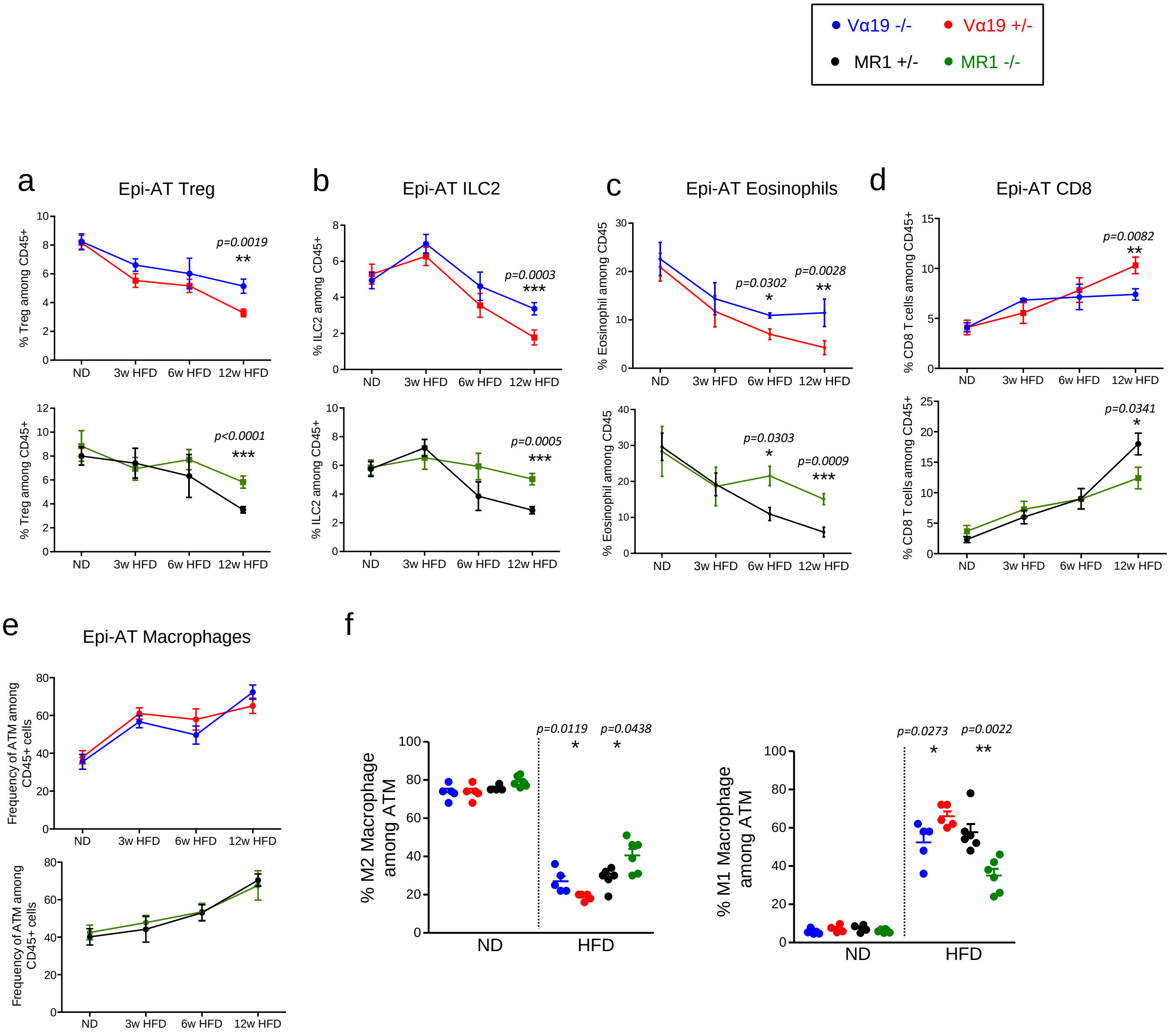

Supplementary Figure 10 related to Figure 4: MAIT cells impact on Epi-AT immune cell populations during progression of obesity. (a-e) Kinetic analysis of the frequency of Foxp3+ Treg cells, CD8, ILC2, eosinophils and macrophages among $CD45^+$ cells in the Epi-AT stroma-vascular fraction (SVF) of $V\alpha19^{+/-}$ and $MR1^{-/-}$ mice and their respective littermate controls ($V\alpha19^{-/-}$ and MR1+/-) fed with ND or HFD during 3, 6 or 12 weeks (n=4 mice per group). (f) Epi-AT macrophage sub-populations in $V\alpha19^{+/-}$ (n=5) and $MR1^{-/-}$ (n=6) mice and their respective littermate controls ($V\alpha19^{-/-}$ (n=5) and MR1+/- (n=6)) after 12-16 weeks of HFD or ND feeding. Data show the frequency of M2 ($CD206^+CD11c$) and M1 ($CD206^-CD11c^+$) among $CD11b^+F4/80^+$ total macrophages . For f each symbol represents an individual mouse (small horizontal lines indicate the mean ± S.E.M.). In A-E, data are represented as mean ± S.E.M.. All statistical analysis were performed by two-tailed Mann-Withney test. *P<0,05 **P<0,01 ***P<0,001

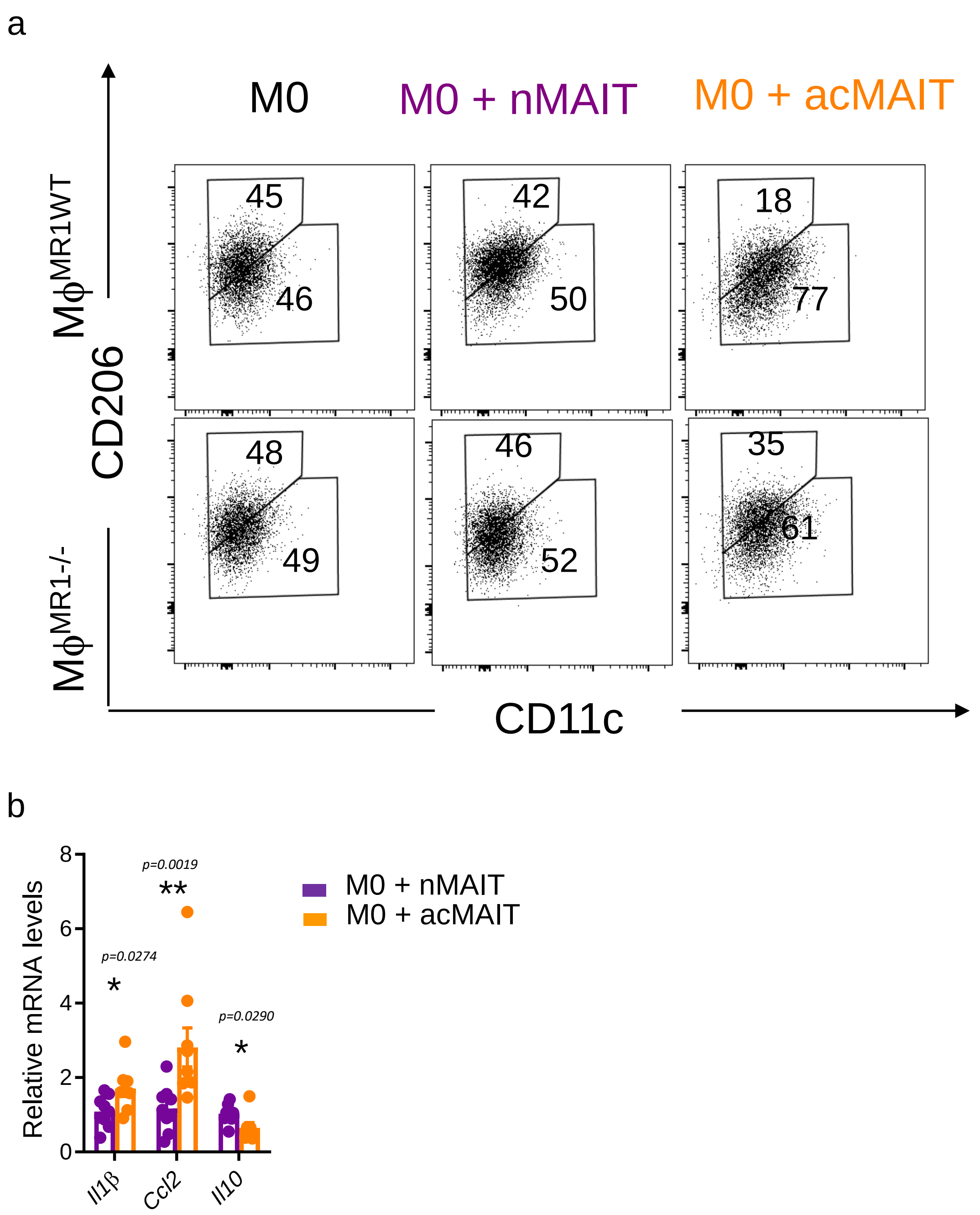

Supplementary Figure 11 related to Figure 5: M1 polarized macrophages increase *Mr1* expression at gene and protein level
(a) Representative dot plots showing M1-macrophages ($CD206^{-}CD11c^{+}$) and M2-macrophages ($CD206^{+}CD11c^{-}$) among $CD11b^{+}F4/80^{+}$ total macrophages. (b) Graphs showing the relative quantity of *Il1β*, *Ccl2* and *Il10* transcript in macrophages co-cultured with nMAIT cells or acMAIT cells (n=9). For A each symbol represents an individual well (small horizontal lines indicate the mean ± S.E.M.). All statistical analyses were performed by two-tailed Mann-Whitney test. *P<0.05 ** P<0.01, *** P<0.001.

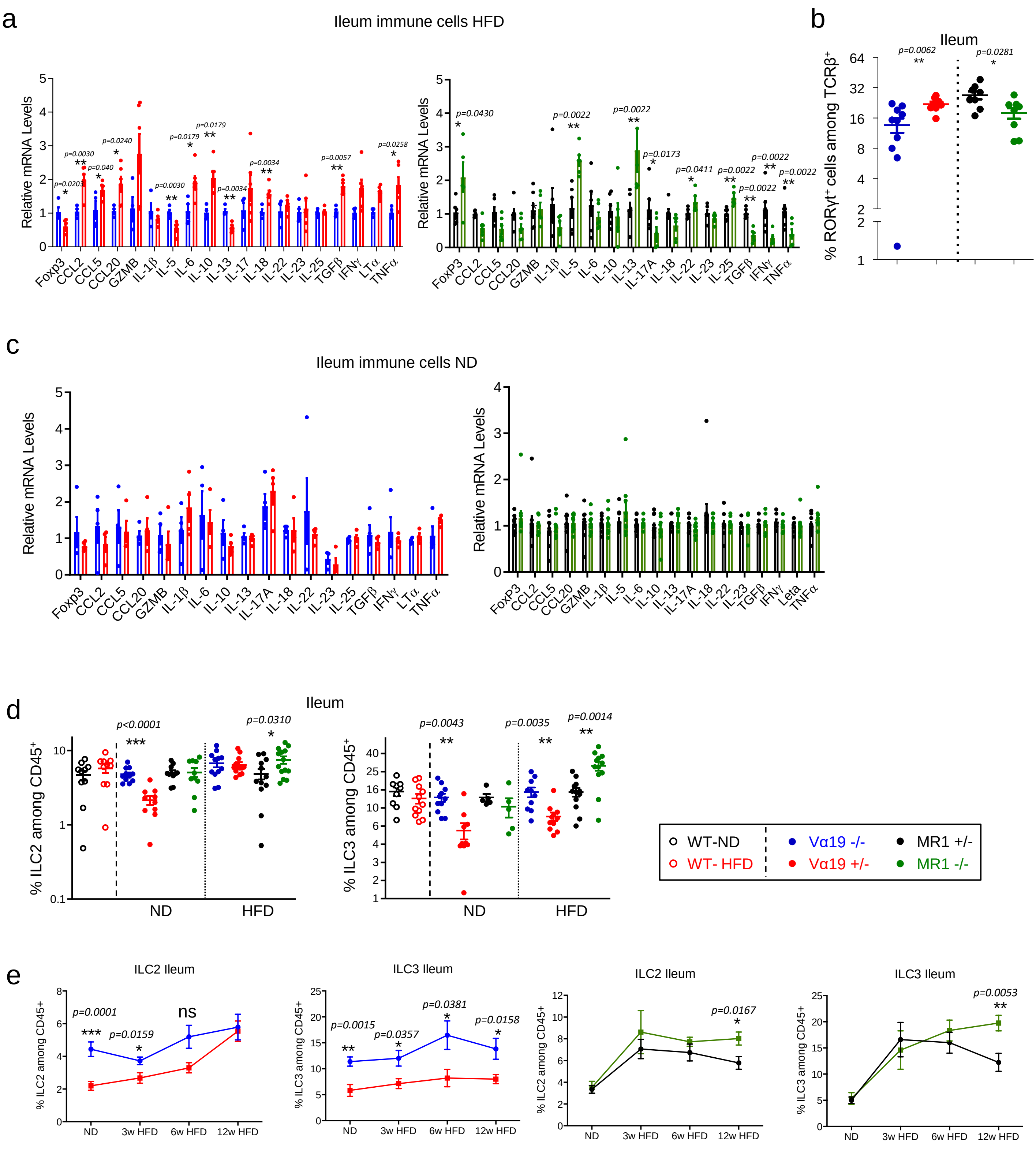

Supplementary Figure 12 related to Figure 6 : MAIT cells induce ileal immune system inflammation during obesity.
(a) Graph showing the relative expression of pro- and anti-inflammatory cytokine-transcripts in the ileal lamina propria of Vα19$^{+/-}$ and MR1$^{-/-}$ mice and their littermate controls fed with HFD (n=14). (b) Frequency of RORγt^{+} cells among αβT cells (Th17) in the and ileal lamina propria (n=9). (c) Graph showing the relative quantity of pro- and anti-inflammatory cytokine transcripts in the ileal lamina propria of Vα19$^{+/-}$ and MR1$^{-/-}$ mice and their littermate controls fed with ND (n=10 per group). (d) Graphs showing the frequency of ILC2 and ILC3 among CD45^{+} cells in the ileum lamina propria of B6 mice fed with ND (n=10) or HFD (n=10), and Vα19$^{+/-}$ (ND n=10, HFD n=13) and MR1$^{-/-}$ (ND n=10, HFD n=14) mice and their respective littermate controls (Vα19$^{-/-}$ (ND n=11, HFD n=12) and MR1$^{+/-}$ (ND n=11, HFD n=12)) after 12-16 weeks of HFD or ND feeding. Pooled data from two or three experiments are represented. (e) Kinetic analysis of the frequency of ILC2 and ILC3 in the ileum lamina propria of Vα19$^{+/-}$ and MR1$^{-/-}$ mice and their respective littermate controls (Vα19$^{-/-}$ and MR1$^{+/-}$) fed with ND or HFD during 3, 6 or 12 weeks (n=6 mice per group) For a, c and e data are represented as mean ± SEM. In b and d each symbol represents an individual mouse (small horizontal lines indicate the mean ± S.E.M.). All statistical analyses were performed by two-tailed Mann-Whitney test. *P<0.05, ** P<0.01, *** P<0.001.

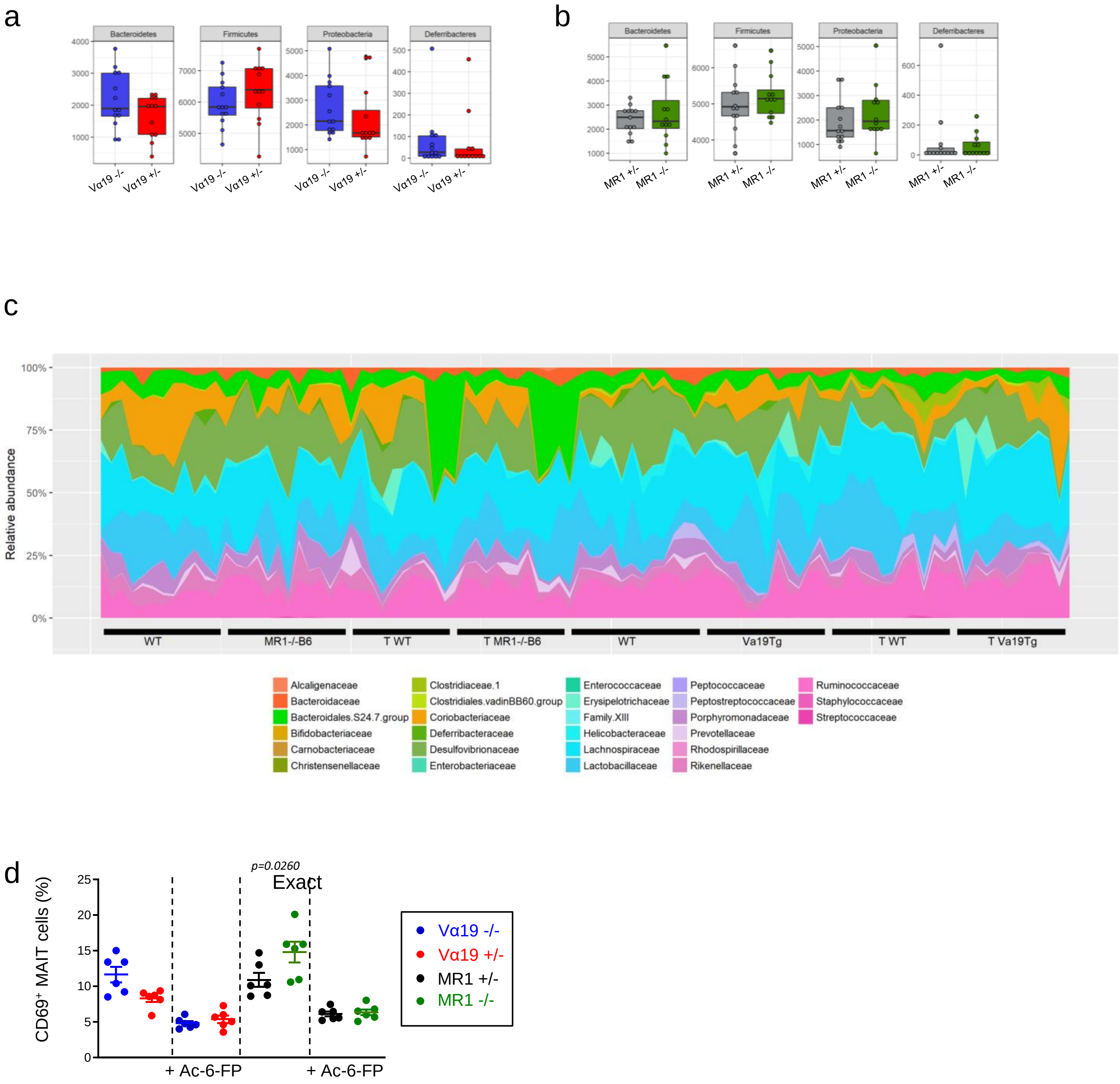

Supplementary Figure 13 related to Figure 6: MAIT cells impact gut microbiota composition

(a) Graphs showing relative abundance of (percentage of 16S rRNA gene sequences) Bacteroidetes, Firmicutes, Proteobacteria, Deferribacteres among the Vα19$^{-/-}$ (n=13) and Vα19$^{+/-}$ (n=12) mice. (b) Graphs showing relative abundance of (percentage of 16S rRNA gene sequences) Bacteroidetes, Firmicutes, Proteobacteria, Deferribacteres among the MR1$^{-/+}$ (n=13) and MR1$^{-/-}$ (n=12) mice after 12 weeks of HFD (c) Overview of the microbiota composition in term of bacteria families among all mouse groups. (d) Analysis of the abundance of MAIT cell activating ligands using a bioassay based on the activation of purified MAIT cells by fecal supernatants from the caecum of Vα19$^{+/-}$ and MR1$^{-/-}$ mice and their respective littermates controls Vα19$^{-/-}$ and MR1$^{+/-}$ fed with HFD (12 weeks) (n=6 mice per group) with or without the antagonist ligand Ac-6-FP. For a and b data are presented as boxplots where the middle line is the median, the lower and upper hinges correspond to the first and third quartiles, the upper whisker extends from the hinge to the largest value no further than 1.5 × IQR from the hinge (where IQR is the inter-quartile range) and the lower whisker extends from the hinge to the smallest value at most 1.5 × IQR of the hinge. The Fold Change and the p-value of a Mann–Whitney test are indicated as mean ± S.E.M. . In d each symbol represents an individual mouse (small horizontal lines indicate the mean ± S.E.M.). All statistical analyses were performed by two-tailed Mann-Whitney test. *P<0.05

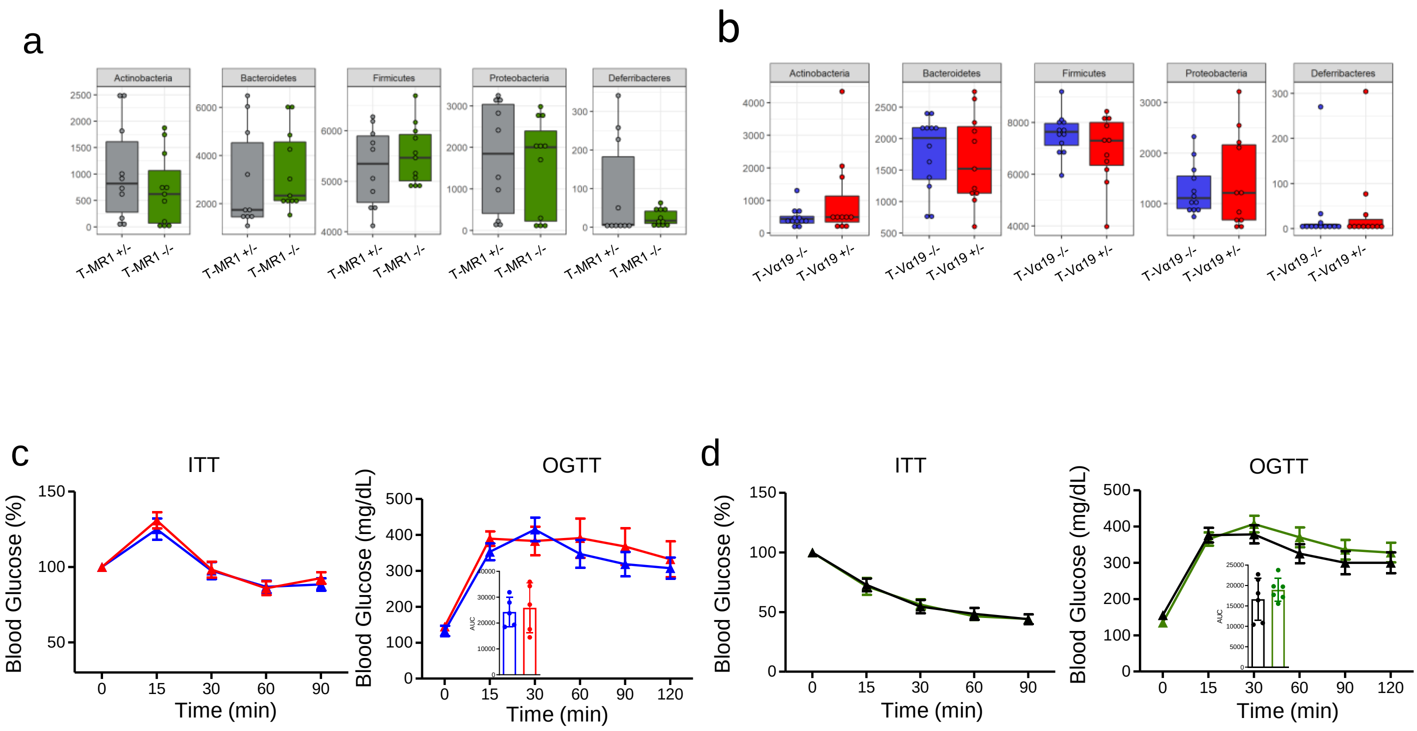

Supplementary Figure 14 related to Figure 7: Analyses of B6 mice transferred with feces from MR1$^{-/-}$ and Vα19$^{+/-}$ mice or co-housed

(a) Graphs showing relative abundance of (percentage of 16S rRNA gene sequences) Actinobacteria, Bacteroidetes, Firmicutes, Proteobacteria, Deferribacteres among the T-MR1$^{-/+}$ (n=10) and T-MR1$^{-/-}$ (n=11) mice (b) Graphs showing relative abundance of (percentage of 16S rRNA gene sequences) Actinobacteria, Bacteroidetes, Firmicutes, Proteobacteria, Deferribacteres among the T-Vα19$^{-/-}$ (n=12) and T-Vα19$^{+/-}$ (n=11) mice. (c) and (d) ITT and OGTT of co-housed Vα19$^{+/-}$ and Vα19$^{-/-}$ littermate controls or co-housed MR1$^{-/-}$ and MR1$^{+/-}$ (n=9 mice per group). For a and b data are presented as boxplots where the middle line is the median, the lower and upper hinges correspond to the first and third quartiles, the upper whisker extends from the hinge to the largest value no further than 1.5 × IQR from the hinge (where IQR is the inter-quartile range) and the lower whisker extends from the hinge to the smallest value at most 1.5 × IQR of the hinge. The Fold Change and the p-value of a Mann–Whitney test are indicated as mean ± S.E.M..In c and d, data are represented as mean ± S.E.M..

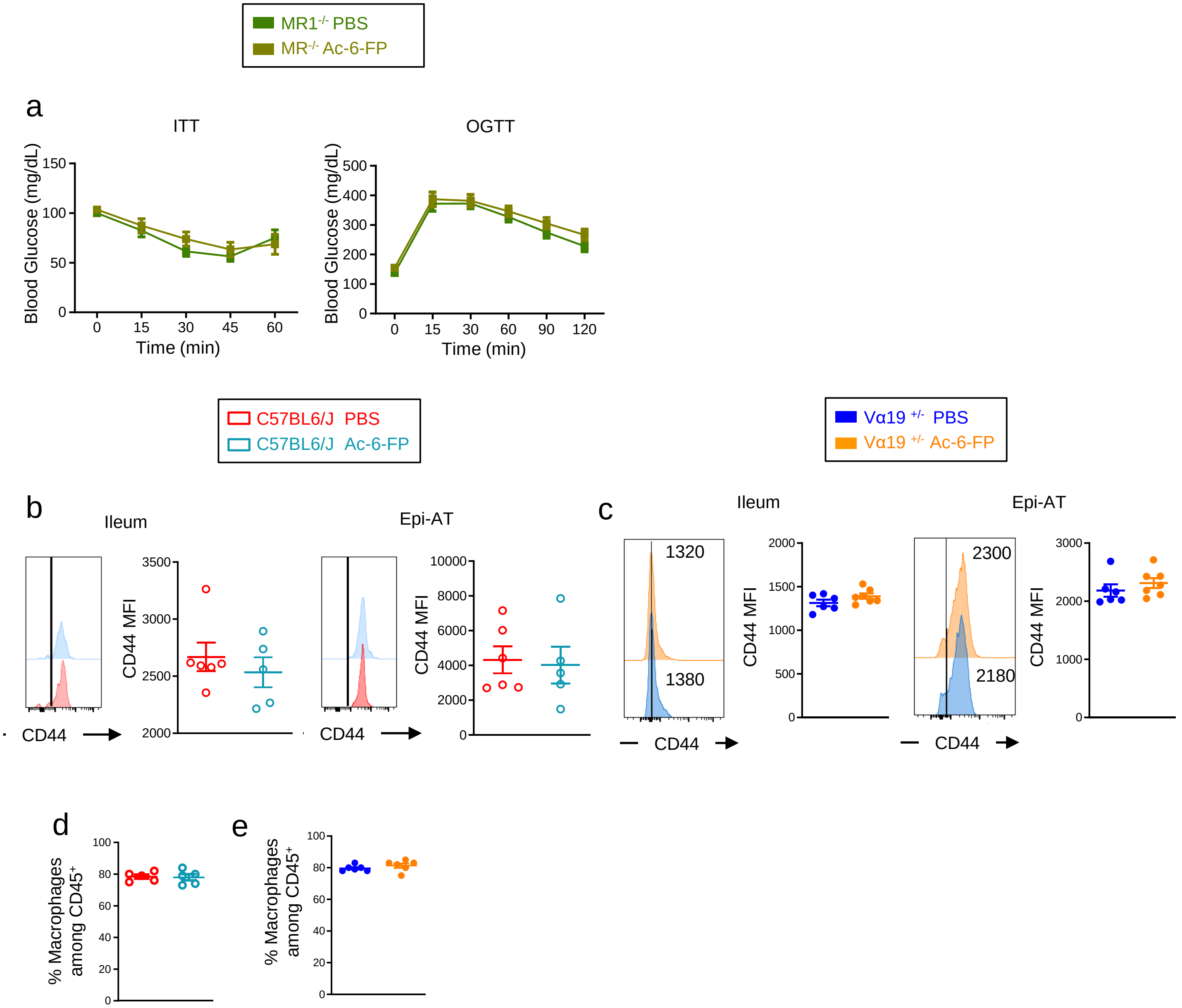

Supplementary Figure 15 related to Figure 8: Effects of Ac-6-FP treatment in C57BL/6J, Vα19$^{+/-}$ or MR1$^{-/-}$ mice during HFD

B6 WT, Vα19$^{+/-}$ or MR1$^{-/-}$ mice were fed during 6 weeks of HFD were then given water containing 50 nM/ml of AC-6-FP (Acetyl-6-formylpterin) (n=6) or PBS (n=6) for the following 8 weeks of HFD and were i.p. injected, twice a week, with 200 µl of Ac-6-FP (50nM) or PBS. (a) ITT and OGTT at 12-weeks of HFD in MR1$^{-/--}$ Ac-6-FP treated mice and MR1$^{-/--}$ control mice (b-c) Representative histograms of CD44 surface marker staining on MAIT cells from ileum and Epi-AT in B6 WT (B) and Vα19$^{+/-}$ (c) mice . (d-e) Frequency of total Epi-AT macrophages among CD45+ cells in B6 WT (d) and Vα19$^{+/-}$ (e) mice . (f) Representative dot plots of M2 (CD206+CD11c-) or M1 (CD206-CD11c+) macrophages staining in Epi-AT of MR1$^{-/--}$ Ac-6-FP treated mice and MR1$^{-/--}$ control mice. For b-e each symbol represents an individual mouse (small horizontal lines indicate the mean ± S.E.M.). In a, data are represented as mean ± S.E.M..

During Obesity

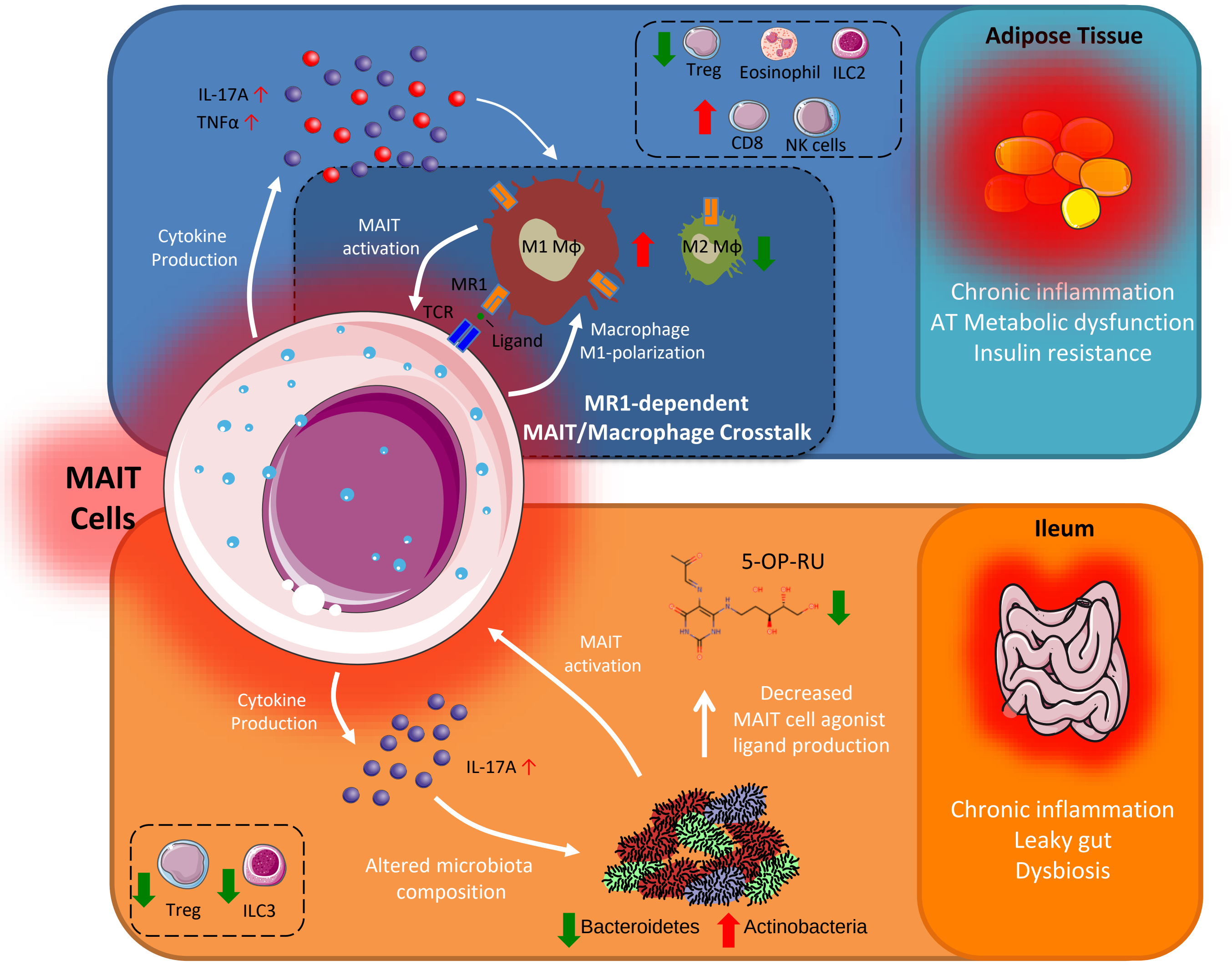

Supplementary Figure 16: Schematic view of MAIT cells role in adipose tissue and ileum inflammation and gut dysbiosis leading to chronic metabolic disorder in the context of obesity.
