# Supplementary material for: Mucosal-associated invariant T cells promote inflammation and intestinal dysbiosis leading to metabolic dysfunction during obesity
Source: Nat Commun. 2020 Jul 24;11:3755. doi: 10.1038/s41467-020-17307-0 (PMC7381641; doi:10.1038/s41467-020-17307-0)
Supplement: Supplementary file 2 — Reporting Summary [file 41467_2020_17307_MOESM2_ESM.pdf]

nature research

Corresponding author(s): DBPR NCOMMS-19-35972A

Last updated by author(s): Jun 15, 2020

Reporting Summary

Nature Research wishes to improve the reproducibility of the work that we publish. This form provides structure for consistency and transparency in reporting. For further information on Nature Research policies, see our Editorial Policies and the Editorial Policy Checklist.

Statistics

For all statistical analyses, confirm that the following items are present in the figure legend, table legend, main text, or Methods section.

n/a	Confirmed	
☐	☒	The exact sample size (*n*) for each experimental group/condition, given as a discrete number and unit of measurement
☐	☒	A statement on whether measurements were taken from distinct samples or whether the same sample was measured repeatedly
☐	☒	The statistical test(s) used AND whether they are one- or two-sided<br>*Only common tests should be described solely by name; describe more complex techniques in the Methods section.*
☒	☐	A description of all covariates tested
☐	☒	A description of any assumptions or corrections, such as tests of normality and adjustment for multiple comparisons
☐	☒	A full description of the statistical parameters including central tendency (e.g. means) or other basic estimates (e.g. regression coefficient) AND variation (e.g. standard deviation) or associated estimates of uncertainty (e.g. confidence intervals)
☐	☒	For null hypothesis testing, the test statistic (e.g. *F*, *t*, *r*) with confidence intervals, effect sizes, degrees of freedom and *P* value noted<br>*Give P values as exact values whenever suitable.*
☒	☐	For Bayesian analysis, information on the choice of priors and Markov chain Monte Carlo settings
☒	☐	For hierarchical and complex designs, identification of the appropriate level for tests and full reporting of outcomes
☒	☐	Estimates of effect sizes (e.g. Cohen's *d*, Pearson's *r*), indicating how they were calculated

Our web collection on statistics for biologists contains articles on many of the points above.

Software and code

Policy information about availability of computer code

Data collection	-Real-Time PCR data were acquired using a LightCycler (480) Roche<br>-Flow cytometry data acquisition was performed using a BD Biosciences LSRFortessa cytometer and cell sorting was performed using BD Biosciences FACSAria III
Data analysis	-Statistical analyses were performed using the GraphPad Prism software version 8.3.0<br>. Flow cytometric analyses were performed with the FlowJo analysis software V10.1 (Tree Star).<br>-ImageJ software was used for quantification of western blot signal.<br>_Mothur (V 1.40.1) software was used to remove PCR primers and sequencing errors<br>-UCHIME (V 9) software was used to remove chimera

For manuscripts utilizing custom algorithms or software that are central to the research but not yet described in published literature, software must be made available to editors and reviewers. We strongly encourage code deposition in a community repository (e.g. GitHub). See the Nature Research guidelines for submitting code & software for further information.

Data

Policy information about availability of data

All manuscripts must include a data availability statement. This statement should provide the following information, where applicable:

- Accession codes, unique identifiers, or web links for publicly available datasets
- A list of figures that have associated raw data
- A description of any restrictions on data availability

The data that support this study are available in figshare with the identifier DOI : 10.6084/m9.figshare.12490271

Field-specific reporting

Please select the one below that is the best fit for your research. If you are not sure, read the appropriate sections before making your selection.

- [x] Life sciences
- [ ] Behavioural & social sciences
- [ ] Ecological, evolutionary & environmental sciences

For a reference copy of the document with all sections, see nature.com/documents/nr-reporting-summary-flat.pdf

Life sciences study design

All studies must disclose on these points even when the disclosure is negative.

Sample size	For in vivo and in vitro experiments, sample size for each experiment has been calculated based on previous data collection published in Fan, Toubal et al. Nature Medicine 2016 and Rouxel et al. Nature Immunology 2017
Data exclusions	For mouse studies if any individual animal shows sign of discomfort or an injection failed, we had to terminate the study for this particular animal according to our ethical permit and rigor to the study.
Replication	For mouse studies, at least three successful independent experiments were performed. Experiments with tissue digestion issues were excluded due to the poor quality of the cells for FACS analysis.
Randomization	All mice were randomized in the cages at weaning (30 days of age).
Blinding	Investigators were blinded for all animal experiments.

Reporting for specific materials, systems and methods

We require information from authors about some types of materials, experimental systems and methods used in many studies. Here, indicate whether each material, system or method listed is relevant to your study. If you are not sure if a list item applies to your research, read the appropriate section before selecting a response.

Materials & experimental systems

n/a	Involved in the study	
☐	☒	Antibodies
☐	☒	Eukaryotic cell lines
☒	☐	Palaeontology and archaeology
☐	☒	Animals and other organisms
☒	☐	Human research participants
☒	☐	Clinical data
☒	☐	Dual use research of concern

Methods

n/a	Involved in the study	
☒	☐	ChIP-seq
☐	☒	Flow cytometry
☒	☐	MRI-based neuroimaging

Antibodies

Antibodies used

Cell suspensions prepared from various tissues were stained at 4°C in PBS containing 5% FCS and 0.1% sodium azide. Surface staining was performed with the following antibodies:

PerCP-Cy5.5 Rat anti-mouse CD45 (30-F11) BD Biosciences 550994
APC/Cy7 anti-mouse CD45 (30-F11) Biolegend 103116
BD Horizon™ BV711 anti-mouse TCR β Chain (H57) BD Biosciences 563135
BD Horizon™ BV421 anti-mouse TCR β Chain (H57) BD Biosciences 562839
Brilliant Violet 605 anti-mouse CD8a (53-6.7) Biolegend 100743
AlexaFluor 700 anti-mouse CD8α (53-6.7) eBioscience 56-0081-82
PE-Cyanine7 anti-mouse CD8α (53-6.7) eBioscience 25-0081-82
BD Horizon™ BV421 anti-mouse CD103 (M290) BD Biosciences 562771
Alexa Fluor 700 anti-mouse CD44 (IM7) eBioscience 56-0441-82
Brilliant Violet 785 anti-mouse CD45.1 (A20) Biolegend 110743
APC anti-mouse CD45.2 (104) Biolegend 109814
Brilliant Violet 711 anti-mouse Ly-6A/E (Sca-1) (D7) Biolegend 108131
Brilliant Violet 510 anti-mouse NK-1.1 (PK136) Biolegend 108737
Brilliant Violet 650 anti-mouse CD19 (6D5) Biolegend 115541
Brilliant Violet 510 anti-mouse CD4 (GK1.5) Biolegend 100449
Brilliant Violet 785 anti-mouse/human CD11b (M1/70) Biolegend 101243
PE/Cy5 anti-mouse CD127 (IL-7Ralpha) (A7R34) Biolegend 135016

PerCP/Cy5.5 anti-mouse CD25 (PC61) Biolegend 102029
Brilliant Violet 605 anti-mouse CD69 (HI-2F3)
Biolegend 104530
PE anti-mouse CD11c (N418)
Biolegend 117308
PerCP-eFluor 710 anti-mouse CD170 (Siglec-F) (IRNM44N) eBioscience 46-1702-80
PE-Cyanine 7anti-mouse F4/80 (BM8) eBioscience 25-4801-82
APC anti-mouse TCRγδ (GL-3) eBioscience 17-5711-82
PE anti-mouse FOXP3 (FJK-16s) eBioscience 12-5773-82
PE-eFluor 610 anti-mouse ROR(t) (B2D) eBioscience 61-6981-80
PerCP/Cy5.5 anti-mouse PLZF (9E12)
Biolegend 145807
Brilliant Violet 60 anti-mouse T-bet (4B10) Biolegend 644817
Brillant Viollet 450 anti-mouse Ki67 (SolA15) eBioscience 48569882
PE anti-mouse CD206 (C068C2) Biolegend 141706
PE-CF594 anti-mouse CX3CR1 (SA011F11) Biolegend 149014
Alexa Fluor 700 anti-mouse CD11c (N418) eBioscience 56-0114-82
Alexa Fluor 647 anti-mouse Bcl-2 (BCL/10C4) Biolegend 633509
eFluor450 anti-mouse TNF (MP6-XT22) eBioscience 48-7321-82
Alexa Fluor 700 anti-mouse IFN-γ (XMG1.2) BD Biosciences 557998
PerCP-Cy5.5 anti-mouse IL-17A (TC11-18H10) BD Biosciences 560666
BD Via-Probe™ Cell Viability Solution BD Biosciences 555815

anti-Akt (polyclonal) Cell signaling 9272S
anti-pAkt (polyclonal) Cell signaling 9331S

Alpha-Galactosylceramide-CD1d tetramer was prepared by the laboratory and coupled to streptavidin-BV421 (Biolegend). Biotinylated mouse MR1 tetramers loaded with the active ligand (5-OP-RU) were used to specifically identify MAIT cells; biotinylated MR1 tetramers loaded with the non-activating ligand acetyl-6-formyl-pterin (Ac-6-FP) were used as a negative control. MR1 tetramers were generated by A.Corbett and J.McCluskey and the NIH facility. MR1 tetramers were coupled to streptavidin-PE (BD Bioscience).

Validation

-Flow Cytometry:
PerCP-Cy5.5 Rat anti-mouse CD45 (30-F11) BD Biosciences 550994 : Ref to publication: PMID: 8974866
APC/Cy7 anti-mouse CD45 (30-F11) Biolegend 103116: Ref to publication: PMID: 21606356
BD Horizon™ BV711 anti-mouse TCR β Chain (H57) BD Biosciences 563135: Ref to publication: PMID: 8986720
BD Horizon™ BV421 anti-mouse TCR β Chain (H57) BD Biosciences 562839 Ref to publication: PMID: 8986720
Brilliant Violet 605 anti-mouse CD8a (53-6.7) Biolegend 100743 Ref to publication: PMID: 25155355
AlexaFluor 700 anti-mouse CD8α (53-6.7) eBioscience 56-0081-82 Ref to publication: PMID: 28572807
PE-Cyanine7 anti-mouse CD8α (53-6.7) eBioscience 25-0081-82 Ref to publication: PMID: 28572807
BD Horizon™ BV421 anti-mouse CD103 (M290) BD Biosciences 562771 Ref to publication: PMID: 8625986
Alexa Fluor 700 anti-mouse CD44 (IM7) eBioscience 56-0441-82 Ref to publication: PMID: 30410056
Brilliant Violet 785 anti-mouse CD45.1 (A20) Biolegend 110743 Ref to publication: PMID: 28767735
APC anti-mouse CD45.2 (104) Biolegend 109814 Ref to publication: PMID: 21300914
Brilliant Violet 711 anti-mouse Ly-6A/E (Sca-1) (D7) Biolegend 108131 Ref to publication: PMID: 31216482
Brilliant Violet 510 anti-mouse NK-1.1 (PK136) Biolegend 108737 Ref to publication: PMID: 30944615
Brilliant Violet 650 anti-mouse CD19 (6D5) Biolegend 115541 Ref to publication: PMID: 25888644
Brilliant Violet 510 anti-mouse CD4 (GK1.5) Biolegend 100449 Ref to publication: PMID: 29081531
Brilliant Violet 785 anti-mouse/human CD11b (M1/70) Biolegend 101243
PE/Cy5 anti-mouse CD127 (IL-7Ralpha) (A7R34) Biolegend 135016 Ref to publication: PMID: 28008921
PerCP/Cy5.5 anti-mouse CD25 (PC61) Biolegend 102029 Ref to publication: PMID: 26673421
Brilliant Violet 605 anti-mouse CD69 (HI-2F3) Biolegend 104530 Ref to publication: PMID: 24998253
PE anti-mouse CD11c (N418) Biolegend 117308 Ref to publication: PMID: 19342633
PerCP-eFluor 710 anti-mouse CD170 (Siglec-F) (IRNM44N) eBioscience 46-1702-80 Ref to publication: PMID: 29891135
PE-Cyanine 7anti-mouse F4/80 (BM8) eBioscience 25-4801-82 Ref to publication: PMID: 29354128
APC anti-mouse TCRγδ (GL-3) eBioscience 17-5711-82 Ref to publication: PMID: 27774091
PE anti-mouse FOXP3 (FJK-16s) eBioscience 12-5773-82 Ref to publication: PMID: 28197366
PE-eFluor 610 anti-mouse ROR(t) (B2D) eBioscience 61-6981-80 Ref to publication: PMID: 29249358
PerCP/Cy5.5 anti-mouse PLZF (9E12) Biolegend 145807 Ref to publication: PMID: 31332204
Brilliant Violet 60 anti-mouse T-bet (4B10) Biolegend 644817 Ref to publication: PMID: 25803478
Brillant Viollet 450 anti-mouse Ki67 (SolA15) eBioscience 48569882 Ref to publication: PMID: 28123877
PE anti-mouse CD206 (C068C2) Biolegend 141706 Ref to publication: PMID: 22504909
PE-CF594 anti-mouse CX3CR1 (SA011F11) Biolegend 149014 Ref to publication: PMID: 27734833
Alexa Fluor 700 anti-mouse CD11c (N418) eBioscience 56-0114-82 Ref to publication: PMID: 29163499
Alexa Fluor 647 anti-mouse Bcl-2 (BCL/10C4) Biolegend 633509 Ref to publication: PMID: 23940724
eFluor450 anti-mouse TNF (MP6-XT22) eBioscience 48-7321-82 Ref to publication: PMID: 28572807
Alexa Fluor 700 anti-mouse IFN-γ (XMG1.2) BD Biosciences 557998 Ref to publication: PMID: 1387110
PerCP-Cy5.5 anti-mouse IL-17A (TC11-18H10) BD Biosciences 560666 Ref to publication: PMID: 8877732
BD Via-Probe™ Cell Viability Solution BD Biosciences 555815 Ref to publication: PMID: 7537649

BV421-CD1d Tetramer Ref to publication: PMID: 2899126
PE-MR Tetramer Ref to publication: PMID: 2899126

-Western Blot:
anti-Akt (polyclonal) Cell signaling 9272S Ref to publication: PMID: 31970200
anti-pAkt (polyclonal) Cell signaling 9331S Ref to publication: PMID: 32186754

Eukaryotic cell lines

Policy information about cell lines

Cell line source(s)	WT3-MR1 cell line was obtained from O.Lantz (co-author of this paper)<br>Jurkat cell lines were used by J McCluskey lab in Figure S4 a and b and previously discribed in Kjer-Nielsen et al. Nature 2012
Authentication	None of the cell lines used were authenticated
Mycoplasma contamination	Yes, cell line was tested for mycoplasma and there is no contamination.
Commonly misidentified lines (See ICLAC register)	No commonly misidentified cell lines were used in the study

Animals and other organisms

Policy information about studies involving animals; ARRIVE guidelines recommended for reporting animal research

Laboratory animals	All the mice used in this study were on C57BL/6J (B6) background. V19+/- transgenic mice that contain a high frequency of MAIT cells (V19Tg), MR1-/- mice lacking MAIT cells and V19+/- C-/- B6 mice for transfer experiments. These mice have been previously described (Martin et al., 2009). To better analyze MAIT cell phenotype, mouse lines were backcrossed with Rorc(t)-GFP Tg C57BL/6J mice (Lochner et al., 2008).<br>All mice used in the studies were males and in all experiments mice of different genotypes were separated at weaning, except when mentioned co-housing. At 8 weeks of age mice from the different mouse lines (n = 3–7 per experiment) were fed with a 60 kJ% fat diet (SSNIFF ref# E15742-347) or with a 10%-fat diet (ND) (SAFE, ref#A03 SP10) for 12 weeks.<br>Animal facility temperature: 22°C and humidity :50%.
Wild animals	No wild animals were used in this study.
Field-collected samples	No samples collected on the field were used in this study.
Ethics oversight	All animal experiments were approved by the ethical committee CEEA34 (APAFIS # 4838 - 2015111715473538 and APAFIS#15383-2018060612382834) and conducted in accordance with the guidelines stated in the International Guiding Principles for Biomedical Research Involving Animals, developed by the Council for International Organizations of Medical Sciences (CIOMS). All mouse strains were bred and maintained in under specific-pathogen free conditions in the mouse facility of Cochin Institute, and at the “Centre exploration fonctionnel (CEF)” at Paris Sorbonne University.

Note that full information on the approval of the study protocol must also be provided in the manuscript.

Flow Cytometry

Plots

Confirm that:

- [x] The axis labels state the marker and fluorochrome used (e.g. CD4-FITC).
- [x] The axis scales are clearly visible. Include numbers along axes only for bottom left plot of group (a 'group' is an analysis of identical markers).
- [x] All plots are contour plots with outliers or pseudocolor plots.
- [x] A numerical value for number of cells or percentage (with statistics) is provided.

Methodology

Sample preparation	The fat pad adipose tissue was isolated from mice and digested with 5-10 mL collagenase H solution (1 mg/mL, Roche ref# 11074059001) at 37°C for 30 min with shaking (150 rpm). After digestion, adipocytes were removed by filtering through a 100m<br>nylon mesh and cell suspension were centrifuged for 5 min at 300 g to pellet the stroma-vascular fraction (SVF). SVF was washed with FACS buffer containing 5% Fetal Calf Serum (FCS, Dominique Dutscher, S1810-500) and 0.1% sodium azide in PBS.<br>Macrophages were directly analyzed from SVF, whereas other immune cells were further enriched on Percoll density gradients<br>of 40 and 80% (GE Healthcare ref# 17-0891-01). The interface between the layers was collected and suspended in PBS containing

5% FCS and 0.1% sodium azide, to retrieve immune cells. After removal of fat tissue, feces and Payer's patches, the intestine were extensively rinsed with HBSS without Ca2+ and Mg2+ containing 10 mM HEPES. Lamina propria cells were isolated using Lamina Propria Dissociation Kit (Miltenyi, 130-097-410) according to the manufacturer's instructions and the cells suspension were enriched by Percoll as for adipose tissue. Liver was perfused with RPMI 1640 medium (Gibco, 6187-010) supplemented with 5% FCS to remove circulating blood cells and then harvested. Liver was passed through 70m mesh and cells suspension
was collected after 2 min centrifugation at 48 g to avoid parenchymal cells. The supernatant was centrifuge again at 440 g and
the pellet was then resuspended in 40% percoll layered onto 80% percoll and centrifuged for 15 min at 780 g at room temperature. The immune cell fraction was collected at the interface and suspended in PBS containing 5% FCS and 0.1% sodium
azide, to retrieve immune cells.

Instrument	BD LSR Fortessa, BD FACSAria III
Software	Data acquired with the Diva software and analyzed with FlowJo
Cell population abundance	The abundance of MAIT cells is a central theme in the publication.
Gating strategy	Gating strategy is well described in the manuscript in Figure S8 and S9

☒ Tick this box to confirm that a figure exemplifying the gating strategy is provided in the Supplementary Information.
