# Supplementary material for: Mucosal-associated invariant T cells promote inflammation and intestinal dysbiosis leading to metabolic dysfunction during obesity
Source: Nat Commun. 2020 Jul 24;11:3755. doi: 10.1038/s41467-020-17307-0 (PMC7381641; doi:10.1038/s41467-020-17307-0)
Supplement: Supplementary file 3 — Source Data [file 41467_2020_17307_MOESM3_ESM.pdf]

Source Data File

Mucosal-Associated Invariant T cells promote inflammation and intestinal dysbiosis leading to metabolic dysfunction during obesity

Toubal et al.

A

OTUs	p value	
Cluster_165	0.014	Vα19$^{-/-}$ vs. Vα19$^{+/-}$
Cluster_175	0.011	
Cluster_298	0.0091	
Cluster_3	0.0019	
Cluster_309	0.0042	
Cluster_179	0.0063	
Cluster_203	0.0085	
Cluster_316	0.0002	
Cluster_323	0.012	
Cluster_342	0.0069	
Cluster_406	0.0016	
Cluster_691	0.0023	
Cluster_640	0.0096	
Cluster_602	0.016	
Cluster_635	0.006	
Cluster_423	0.0077	
Cluster_433	0.0019	
Cluster_647	0.005	

B

OTUs	p value	
Cluster_21	0.0055	MR1$^{+/-}$ vs. MR1$^{-/-}$
Cluster_451	0.0052	
Cluster_556	0.01	

C

OTUs	p value	
Cluster_556	0.0062	T-MR1$^{+/-}$ vs. T-MR1$^{-/-}$
Cluster_204	0.04	
Cluster_162	0.033	
Cluster_114	0.0087	
Cluster_319	0.045	
Cluster_1403	0.02	
Cluster_1293	0.033	
Cluster_451	0.015	
Cluster_21	0.015	

D

OTUs	p value	
Cluster_1187	0.034	T-Vα19$^{-/-}$ vs. T-Vα19$^{+/-}$
Cluster_1067	0.041	
Cluster_1011	0.027	
Cluster_1135	0.045	
Cluster_55	0.0041	
Cluster_325	0.0096	
Cluster_312	0.0006	
Cluster_1243	0.021	
Cluster_1371	0.049	
Cluster_1681	0.03	
Cluster_1883	0.018	
Cluster_2336	0.041	
Cluster_2164	0.038	
Cluster_309	0.023	
Cluster_2203	0.025	
Cluster_254	0.023	
Cluster_262	0.037	
Cluster_139	0.048	
Cluster_275	0.038	
Cluster_563	0.0005	

Source Data File: p value for OTU clusters in Figures 6 and 7

Figure 3c

pAKT and AKT Vα19+/- vs Vα19-/- (+/- insulin)

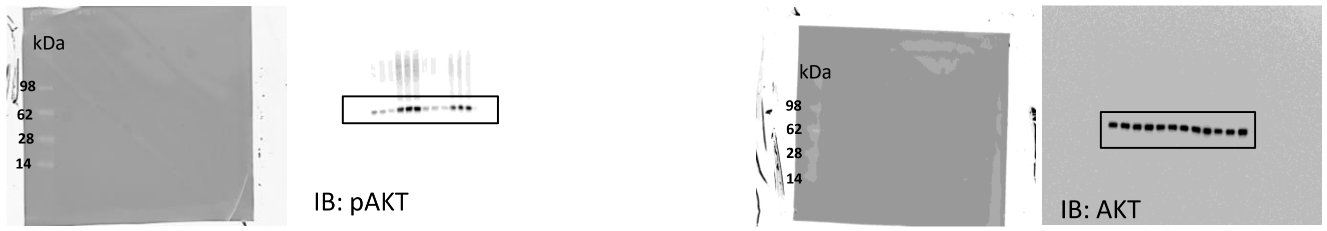

pAKT and AKT MR1+/- vs MR1-/- (+/- insulin)

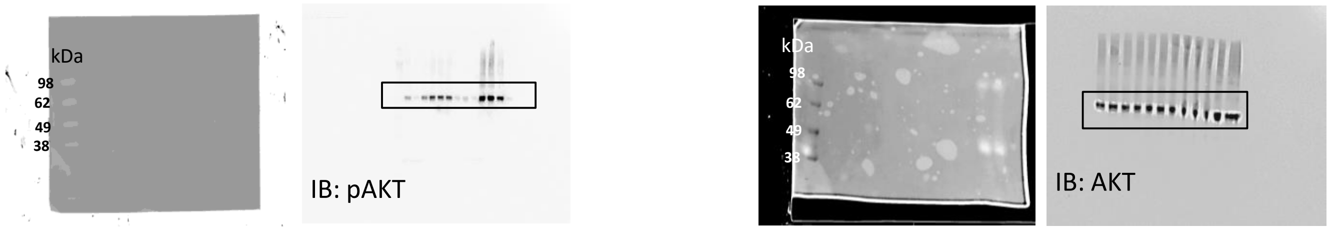

Figure S5b

Muscle pAKT and AKT Vα19+/- vs Vα19-/- (+/- insulin)

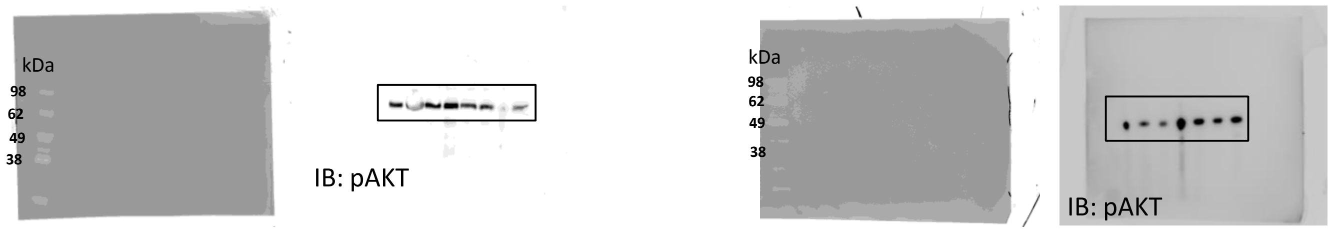

Muscle pAKT and AKT MR1+/- vs MR1-/- (+/- insulin)

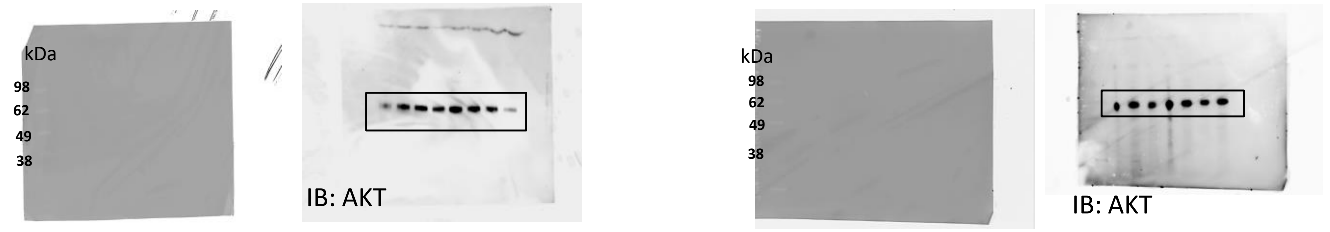

Figure S5c

Liver pAKT and AKT Vα19+/- vs Vα19-/- (+/- insulin)

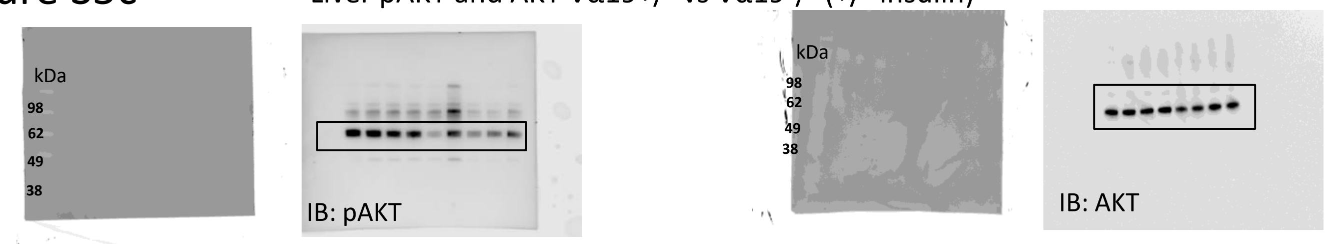

Liver pAKT and AKT MR1+/- vs MR1-/- (+/- insulin)

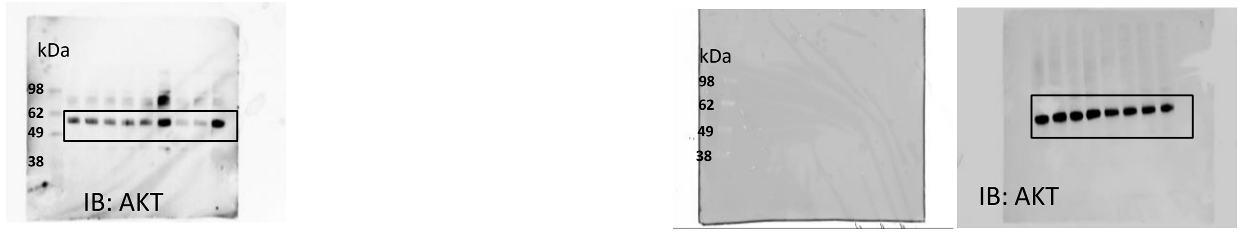

Source Data related to Figure3 and Figure S5: Uncropped Blots

Groups	Sample number
Vα19-/-	13
Vα19+/-	12
Vα19-/- microbiota transfer (T-Vα19$^{-/-}$)	12
Vα19+/- microbiota transfer (T-Vα19$^{+/-}$)	11
MR1+/-	13
MR1-/-	12
MR1+/- microbiota transfer (T-MR1$^{+/-}$)	10
MR1-/- microbiota transfer (T-MR1$^{-/-}$)	11

Source Data related to Figure 6 and Figure S13 and S14: Group content for 16s sequencing
